# Development of Purine and Pyrrolopyrimidine Scaffolds as Potent, Selective, and Brain Penetrant NUAK1 Inhibitors

**DOI:** 10.1021/acsmedchemlett.5c00467

**Published:** 2025-09-10

**Authors:** Gregory G. Aldred, Helen K. Boffey, Henriette M. G. Willems, David Winpenny, Helen Scott, Jonathan H. Clarke, Stephen P. Andrews, John Skidmore

**Affiliations:** The ALBORADA Drug Discovery Institute, University of Cambridge, Island Research Building, Cambridge Biomedical Campus, Hills Road, Cambridge CB2 0AH, United Kingdom

**Keywords:** NUAK1, Kinase selectivity, ADME properties, In vivo profile

## Abstract

NUAK1 is a protein kinase with various cellular functions including cell proliferation, migration and adhesion. NUAK1 has also been implicated in tau phosphorylation and stabilization leading to interest in this kinase as a therapeutic target for neurodegenerative disease. Herein, we describe the optimization of the CDK2 inhibitor NU6140 to the potent and selective NUAK1 inhibitor ARUK2010694, with a significantly improved mouse plasma half-life. Further development of this series also led to the discovery of ARUK2010489, a highly brain penetrant NUAK1 inhibitor (unbound brain/plasma ratio in mice (K_p,u,ub_) of 2.29) that displays no CDK2 activity, applicable for CNS pharmacological studies.

## Graphical abstract

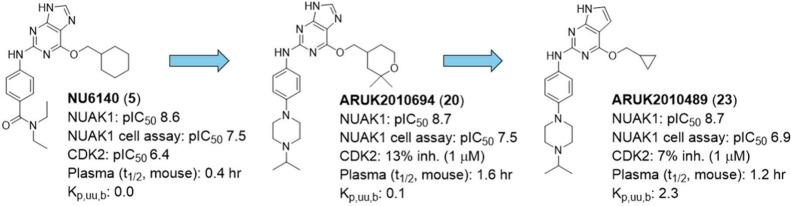

The NUAK1 (ARK5) is a member of the family of AMP-activated protein kinase (AMPK)-related serine/threonine kinases (ARKs). AMPK acts as a cellular ATP sensor and is responsible for the initiation of diverse pro-survival mechanisms in response to metabolic cell stress.^1^ The ARKs comprise 12 enzymes that share significant homology to the AMPK catalytic subunit although none have the ATP sensing function attributed to AMPK.^2,3^ The majority of kinases in this family are activated by the tumor-suppressor liver kinase B1 (LKB1/STK11) and hence have been implicated as oncogenic targets.^1^

NUAK1 and the closely related paralog NUAK2 have been shown to have developmental roles in neurulation with gene mutations resulting in the omphalocele phenotype in mice.^4,5^ NUAKs are involved in a broad range of cellular functions including cell adhesion and migration^6,7^ and are dysregulated in various cancers^8–12^ suggesting a role in tumor survival and metastasis.^13,14^ Conversely, the role of NUAK1 in cell cycle progression and senescence suggests a complex duality of pro- and anticancer functions for this target that may be dependent on cellular context.^15,16^ These potential overlapping biological activities, dependent on cell type and developmental stage, suggest that NUAKs may also be important in other diseases.^17^ NUAK1 has been shown to have a role in neuronal shaping and branching^18,19^ and is linked to neurodegenerative disease.^20^ The characteristic accumulation of aggregated, hyperphosphorylated tau protein in tauopathies such as Alzheimer’s disease (AD)^21^ is associated with increased levels of NUAK1 protein in patient post-mortem brain samples.^20^ These researchers also showed that NUAK1 was able to directly phosphorylate tau at Ser356, which led to increased levels of hyperphosphorylated tau in cells, and that 50% haploinsufficiency of NUAK1 expression was able to rescue phenotypic and behavioral deficits from a tauopathy mouse model.^20^ Furthermore, treatment with the NUAK1 inhibitor WZ4003 ^22^ (**1**) was able to reduce levels of tau phosphorylated at Ser356 in organotypic slice cultures.^23^ The overexpression of NUAK1 in both cancer and in neurodevelopmental disorders and neurodegenerative disease has led to interest in repurposing existing drugs and developing new inhibitors for NUAK1 with improved pharmacological properties.^17^

Compound **1** and the NUAK1 inhibitor HTH-01-015 ^22^ (**2**) have been used extensively in cell-based experiments^10,20,23–25^ but in our hands these compounds demonstrate low target engagement in a cell-based NanoBRET assay. Also, these compounds do not have published ADME or in vivo data (Figure 1). A search for NUAK ligands using the CHEMBL33 database identified 14 compounds with NUAK1 or NUAK2 IC_50_ values of <300 nM that had in vivo data published, but all showed higher potency toward other protein kinases (see Table S1). A recent publication reported the development of UCB9386 (**3**) from a high throughput screening hit and is the first reported NUAK1-selective inhibitor that demonstrates brain penetration.^26^ The CDK/NUAK1 inhibitor ON123300 (Naraziclib, **4**) is also brain penetrant but is more potent toward the CDK kinases than NUAK1. We have developed analogues of **4** with enhanced selectivity and cell potency toward NUAK1.^27^ While these compounds are brain penetrant, further optimization is required to enhance in vivo exposure.

**Figure 1:**
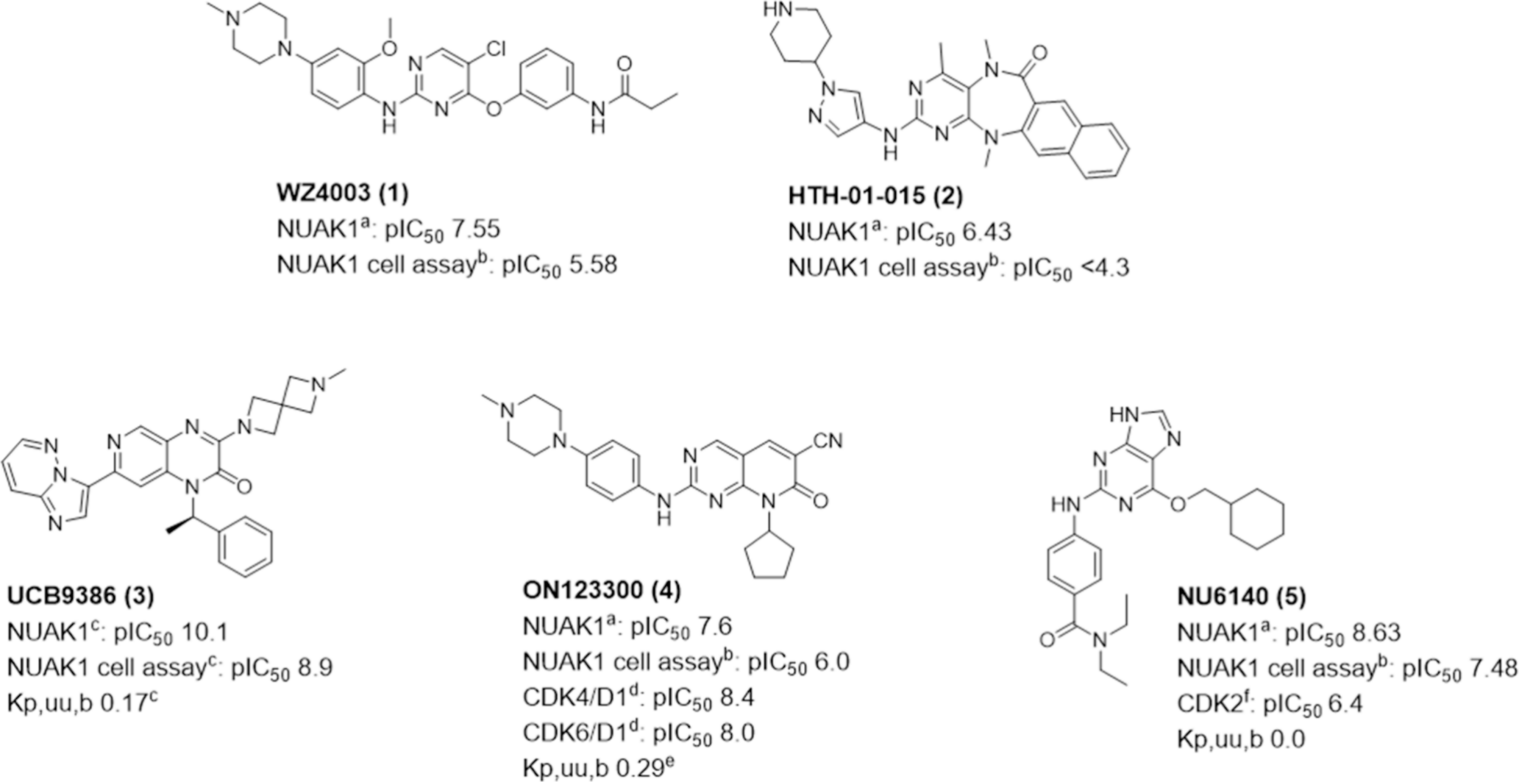
NUAK1 inhibitors ^a^Promega ADP-Glo kinase assay, ^b^cell-based NanoBRET target engagement assay, ^c^data from Poullennec et al.,^26^ ^d^data from Reddy et al.,^28^ ^e^data from Rooney et al.,^27^ ^f^data from Jorda et al.^29^

The CDK2 inhibitor NU6140 (**5**)^30,31^ (Figure 1) has also demonstrated activity against NUAK1 in a broad kinase activity screen.^32^ In our hands, **5** was a highly potent inhibitor of NUAK1 in an ADP-Glo biochemical assay and showed good levels of cellular target engagement in a NanoBRET assay. Moreover, **5** has over 100-fold greater potency toward NUAK1 than toward CDK2. However, this compound had poor metabolic stability when incubated with mouse liver microsomes (MLM *t*_1/2_ 4.7 min) and was highly effluxed in an MDCK-MDR1 model of permeability (ER 13.4), suggesting brain exposure may be limited. Thus, to deliver an effective brain penetrant compound to investigate the role of NUAK1 in the CNS, the aim was to improve the physicochemical properties of **5** to enhance metabolic stability and reduce efflux. The other key objective was to establish selectivity vs homologous kinases while maintaining NUAK1 potency. Herein, we describe the development of highly potent and selective NUAK1 inhibitors with different profiles suitable for both peripheral and CNS in vivo studies.

We chose MARK3 as the primary selectivity target due to its significant homology to NUAK1 (49.4% human kinase-domain homology). MARK3 has also been shown to phosphorylate tau and inhibition of this kinase has shown a potential link to adverse effects on blood pressure.^33^ We also screened selected compounds against the closely related kinase NUAK2 and against CDK2 as the published primary target of **5**, together with its homologues, CDK4 and CDK6.

There are currently no crystal structures of NUAK1 publicly available, hence we developed a series of homology models based on structures of the MARK kinases. Compound **5** docked well into a model based on MARK3 (Figure 2, PDB: 7P1L,^34^ see SI for details of model development) showing the key hinge binding interactions of the aminopurine core. In this model, Leu185, Lys205 and Leu209 form a hydrophobic pocket that accommodates the cyclohexyl moiety.

**Figure 2:**
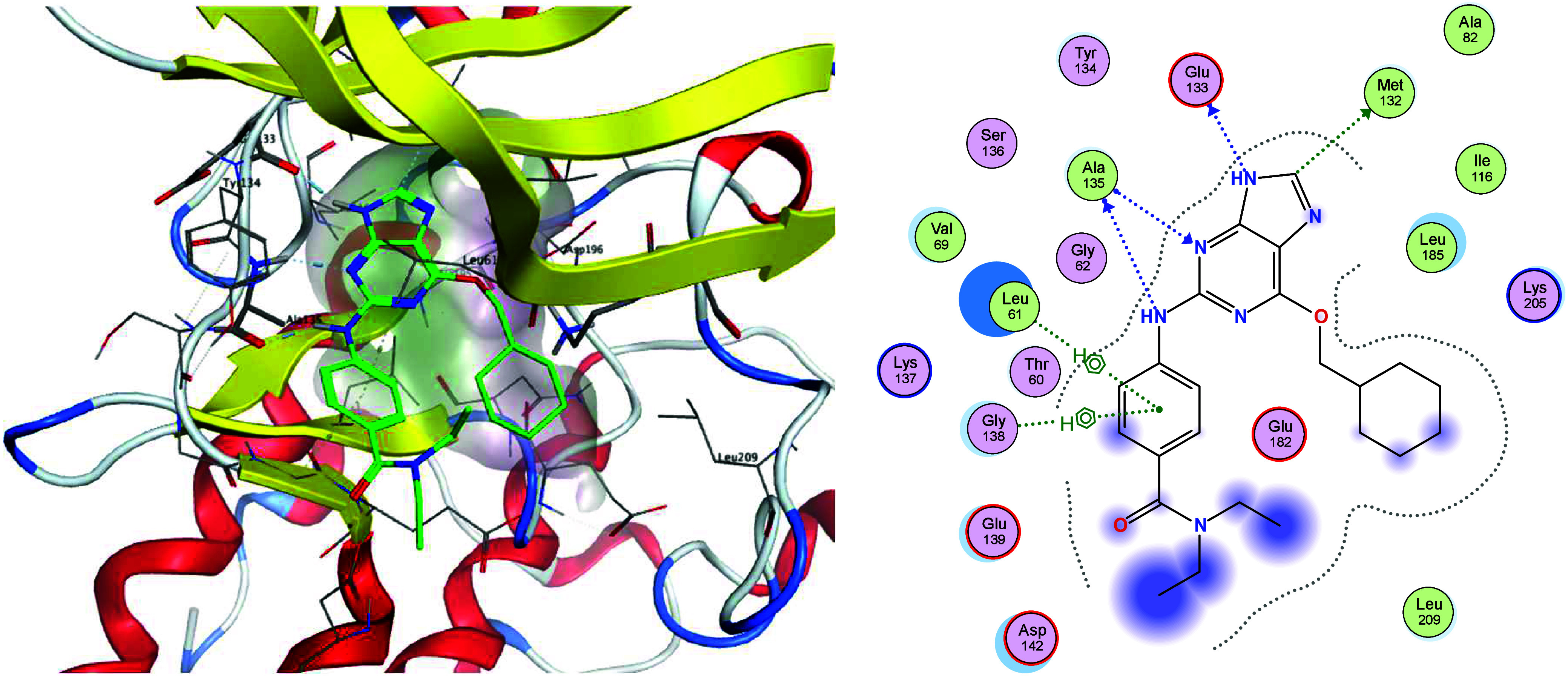
Best docking pose (GOLD) of **5** (green) in a homology model of NUAK1 based on MARK3 PDB structure 7P1L, with 2D interaction map.

With respect to NUAK1 potency, changes to the amide of **5** were well tolerated, giving compounds that remained selective against MARK3 (**6** to **9**, see Table 1). The homology model suggests that the amide of **5** does not make a specific interaction with the protein. Indeed, removal of the entire substituent R1 to leave the unsubstituted aniline **10** (NU2058 ^35^) gave only a moderate reduction in potency in the biochemical assay although a greater reduction was observed in the cell-based assay, potentially due to diminished permeability. A methoxy pyrazole group in this position was well tolerated (**11**) although also showed a drop in cell potency. However, replacement of the amide within the R1 substituent with a piperazine (analogous to **1** and **4**) improved the cell-based potency (see Table 1; **12** and **13**). These analogues potentially form a salt bridge interaction with Asp142 on the edge of the ATP-binding pocket. MARK3 selectivity is partially reduced by the presence of a piperazine, particularly when substituted in the meta position (**13**). However, an advantage of removing the amide functionality is the reduction of TPSA, which is likely to be preferential for CNS penetration,^36^ hence our focus remained on piperazine-containing analogues.

**Table 1:**
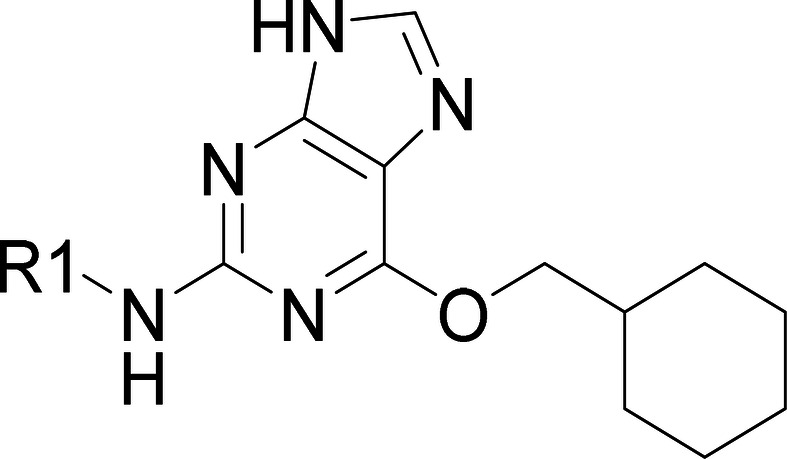

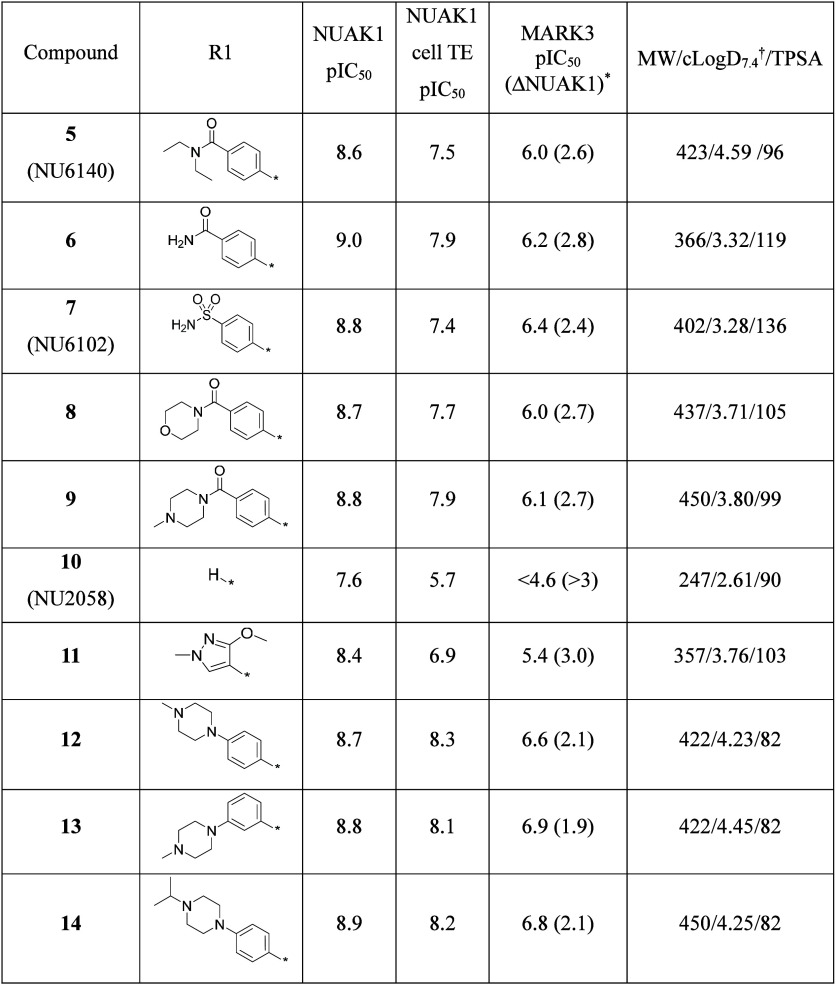
Effect of Aniline Changes on NUAK1 and MARK3 Potency^a^

a NUAK1: ADP-Glo assay; NUAK1 cell TE: cell-based NanoBRET target engagement assay; *ΔNUAK1: NUAK1 pIC_50_ minus MARK3 pIC_50_; †cLogD_7.4_ calculated using SimulationsPlus ADMET Predictor

We next explored changes to the core of the series. *N*-methylation of the imidazole ring led to reduced potency with 7-methyl substituted **15** and negligible potency with 9-methyl substituted **16** (see Figure 3). This observation supports the hypothesis that the 9-position NH is important in receptor binding, potentially to Glu133 in the hinge backbone of NUAK1. This interaction can be seen in the homology model and is analogous to that in the crystal structure of **5** in CDK2 where the equivalent nitrogen interacts with residue Glu321 (PDB: 6JGM^37^).

**Figure 3:**
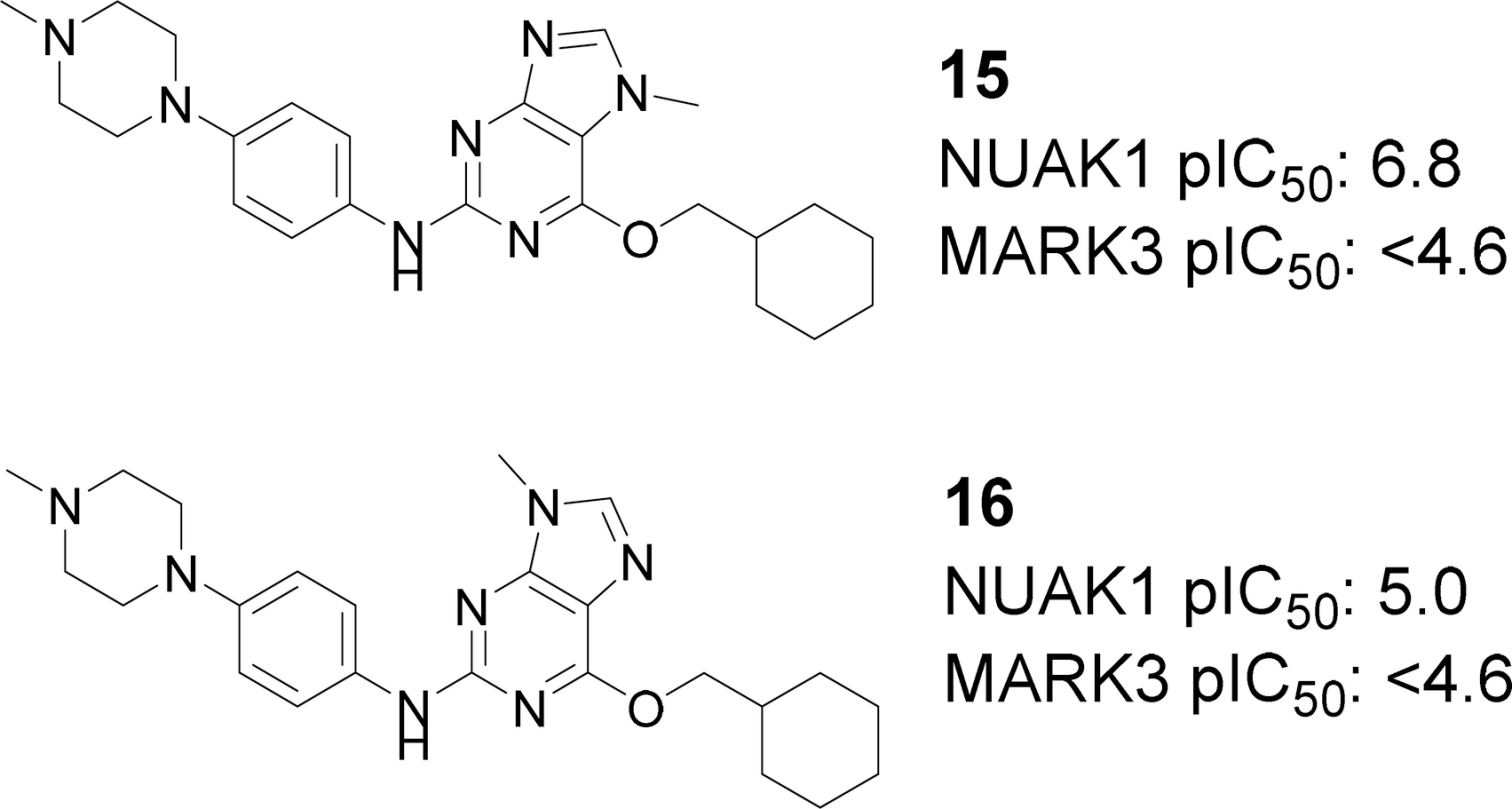
Effect of methylation of purine core on NUAK1 and MARK3 potency.

While the TPSA of piperazines **12** and **14** is low, these compounds have moderately high lipophilicity. We explored tuning the physicochemical properties through the ether substituent (R2; see Table 2). Replacement of the cyclohexyl ring with a methoxyethyl chain or smaller trifluoroethyl group retained potency in the biochemical assay and reduced lipophilicity but significantly lowered cell potency (compare **17** with **5** and **18** with **14**). Cyclic groups including cyclopropyl (**19**) and 2,2-dimethyltetrahydro-2H-pyran (**20**) reduced lipophilicity compared to **14** and were more potent than **18** in the cell-based assay.

**Table 2:**
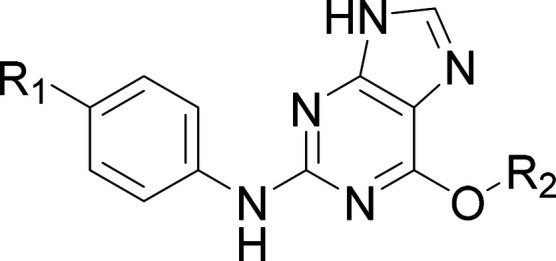

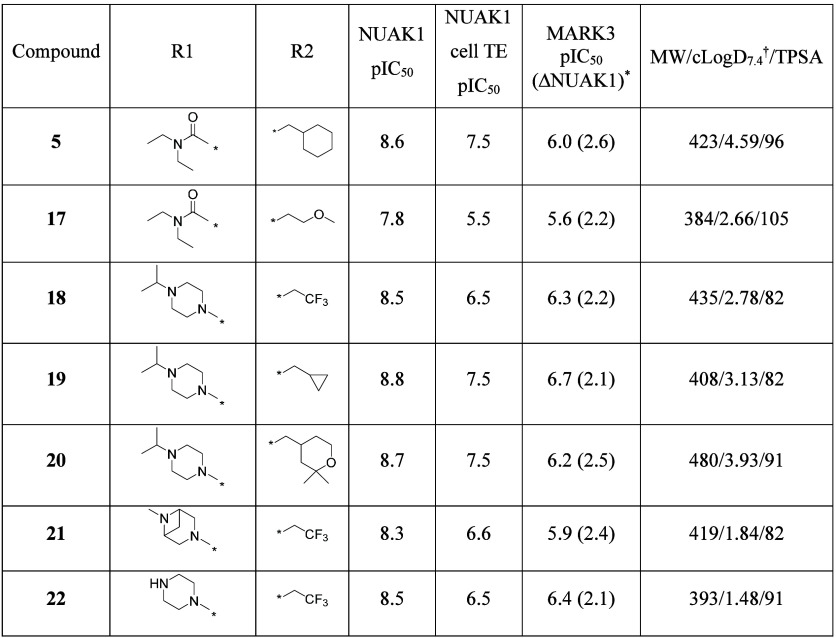
Effect of Changes to Cyclohexyl and Piperazine Groups on NUAK1 and MARK3 Potency^a^

a NUAK1: ADP-Glo assay; NUAK1 cell TE: cell-based NanoBRET target engagement assay; *ΔNUAK1: NUAK1 pIC_50_ minus MARK3 pIC_50_; †cLogD_7.4_ calculated using SimulationsPlus ADMET Predictor

Replacement of the *N*-iso-propylpiperazine with diazabicyclo[3.1.1]­heptane or unsubstituted piperazine reduced lipophilicity without loss of potency (compare **21** and **22** with **18**). However, the additional hydrogen bond donor in **22** may reduce brain permeability.^36^

The difference in potencies between the biochemical and cell-based assays appear to negatively correlate with LogD_7.4_ values (see compound tables for calculated LogD_7.4_ values), which suggests cell-membrane permeability may be a factor in the potency shifts observed.^38^ In addition to this, high intracellular concentrations of ATP relative to its K_m_ can lead to a discrepancy in potency when transitioning to a cell-based assay. Ideally, the biochemical assay would be compared to a functional cell-based assay of NUAK1 activity (e.g., measurement of phospho-MYPT levels^7^) but we were unable to establish a robust assay for this purpose.

Compounds tested against MARK1, MARK2 and MARK4 showed similar selectivity trends as toward MARK3 (see Table S2). The amide-substituted aniline analogues were highly selective toward NUAK1 whereas piperazine-substituted analogues were less so, with **20** as the exception. Interestingly, **12** was the only compound tested that was more potent toward other MARK kinases than MARK3.

Selected compounds were tested against NUAK2 (see Table S3). Cyclohexyl analogues were not significantly selective toward NUAK1 (<3-fold) but the tetrahydropyran analogue **20** was 12-fold selective.

As NU6140 is a known CDK2 inhibitor, we screened selected compounds against a panel of CDK kinases (see Table 3). The presence of the cyclohexyl in **12** and **14** partially retains CDK2 activity and appears to confer potency toward CDK4 and CDK6. However, as with NUAK2, changing the cyclohexyl to either a cyclopropyl (**19**) or notably a tetrahydropyran (**20**) leads to a reduction of CDK activity.

**Table 3:** CDK Kinase/Cyclin Activity Data, Single Point at 1 μM

Compound	NUAK1 pIC_50_	CDK2/A^a^ % inhibition at 1 μM	CDK4/D1^b^ % inhibition at 1 μM	CDK6/D1^b^ % inhibition at 1 μM
**12**	8.7	58	95	81
**14**	8.9	52	93	84
**19**	8.8	34	79	66
**20**	8.7	13	63	32

a Z′-LYTE screening assay.

b Adapta screening assay.

In a panel of 140 protein kinases, **20** inhibited only 5 kinases in addition to NUAK1 with <20% activity remaining compared to the control at 1 μM. IC_50_ values for these 5 kinases were ≥ 10-fold that for NUAK1 (see Table S4), offering a complementary selectivity profile to that of **3**.

An aim of this project was to improve the metabolic stability of **5**. The iso-propylpiperazine analogue **14** showed no improvement in mouse liver microsome half-life (MLM *t*_1/2_) compared to **5** (see Table 4), hence we investigated the metabolic liability of the cyclohexyl-methyl ether substituent. The cyclopropyl-methyl analogue **19** showed significantly higher microsomal stability compared with **14** but had low permeability and high efflux in the MDCK-MRD1 assay. Permeability and efflux were marginally improved in **20** compared with **14**, and this compound also had a promising MLM *t*_1/2_ of 41.8 min. While the trifluoroethyl analogues **21** and **22** had excellent half-lives, their cell potencies were not sufficient for progression.

**Table 4:** ADME Properties of Purine Analogues

Compound	MLM *t*_1/2_ (mins)	MDCK-MDR1 permeability P_app_ (cm s^–1^) (ER)	PPB F_u_	BPB F_u_
**5**	4.7	3.26 (13.4)	0.005	n.d.
**14**	4.3	n.d.	n.d.	n.d.
**19**	100	0.39 (132)	n.d.	n.d.
**20**	41.8	1.79 (19.7)	0.095	0.039
**21**	158	n.d.	n.d.	n.d.
**22**	221	n.d.	n.d.	n.d.

To explore the correlation of microsomal half-life to in vivo pharmacokinetics we coadministered **5** and **20** in a mouse PK study (5 mg/kg, I.P., Table 5). The plasma half-life increased from 0.43 h for **5** to 1.55 h for **20** in correspondence with the MLM *t*_1/2_, but brain exposure was negligible for both compounds, presumably due in part to high efflux.

**Table 5:** Pharmacokinetic Data for Purine Analogues (Mouse, 5 mg/kg, I.P.)

Parameter	Compound **5**	Compound **20**
Plasma *t*_1/2_ (hr)	0.4	1.6
AUC_inf_ (hr.ng/mL)	818	1015
[Brain] 30 min/2 h (ng/mL)	14/BQL	36/13
Brain/Plasma (30 min/2 h)	0.0/BQL	0.1/0.2
K_p,uu,b_ (30 min/2 h)	n.d.	0.02/0.07

In summary, purine-based compounds containing a cyclohexyl moiety show excellent cellular target engagement for NUAK1 (pIC_50_ > 8) but are metabolically unstable and not selective over the CDK kinases. Replacement of the cyclohexyl group with a 2,2-dimethyltetrahydro-2H-pyran maintains potent cell-based activity (pIC_50_ 7.5) and enhances metabolic stability and kinase selectivity, hence we propose **20** (ARUK2010694) as a tool compound for investigating the peripheral role of NUAK1 in vivo.

To investigate if these compounds could be developed further for CNS penetration, an alternative core to the purine was explored. To reduce the TPSA, a selection of pyrrolopyrimidine analogues was prepared (Table 6). Direct match pair analysis with purine-based compounds showed varying relative potencies in the cell-based assay (compare **23** with **19** and **24** with **18**). Bis-substitution of the cyclopropyl with fluorine furnished **25** which had the same biochemical potency as **23** but marginally improved cell potency (pIC_50_ 7.1). Substitution with 4-hydroxy-1-methylindole to afford **26** increased the cell potency further but significantly increased the lipophilicity compared to **23**.

**Table 6:**
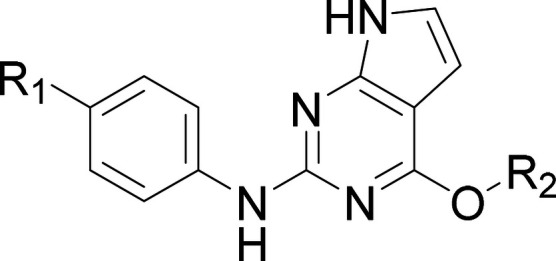

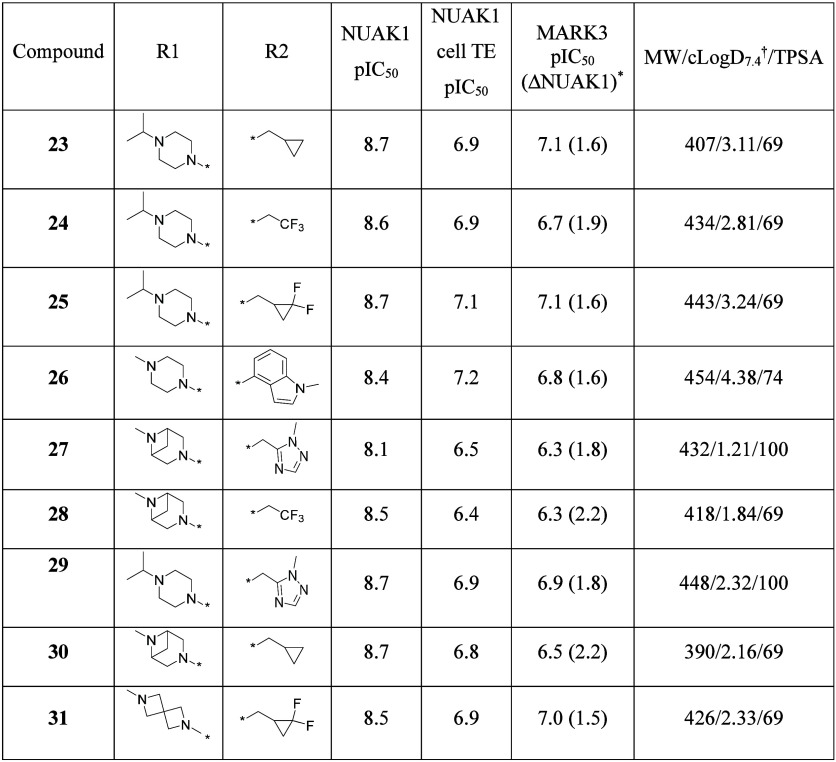
Effect of Substitution of Pyrrolopyrimidine Core on NUAK1 and MARK3 Potency^a^

a NUAK1: ADP-Glo assay; NUAK1 cell TE: cell-based NanoBRET target engagement assay; *ΔNUAK1: NUAK1 pIC_50_ minus MARK3 pIC_50_; †cLogD_7.4_ calculated using SimulationsPlus ADMET Predictor

Using the pyrrolopyrimidine core, additional piperazine replacements were explored including diazabicyclo[3.1.1]­heptane and diazaspiroheptane, resulting in compounds with lower cLogD_7.4_ values. Some diazabicyclo[3.1.1]­heptane analogues showed a slight reduction in cell potency (compare **27** and **28** with **29** and **24** respectively) whereas the cyclopropyl analogue **30** was as potent as its iso-propylpiperazine equivalent **23**. Diazaspiroheptane analogue **31** was slightly less potent than **25**.

It is notable that selectivity over MARK3 was significantly reduced in this series compared to purine-based NUAK1 inhibitors, with diazabicyclo[3.1.1]­heptane analogues showing slightly more selectivity than the iso-propylpiperazines.

Other cores were also investigated including removal of the purine N3 nitrogen to give a pyrrolopyridine (**32**, see Table 7). This transformation reduced potency (compare **32** with **28**), as did moving the nitrogen in the pyrrole ring (**33**) and replacement of the purine with an imidazo­[1,2-*b*]­pyridazine (**34**). The pyrazolopyrimidine **35** was more promising but significantly less potent than the pyrrolopyrimidine **25** in the cell-based assay.

**Table 7:**
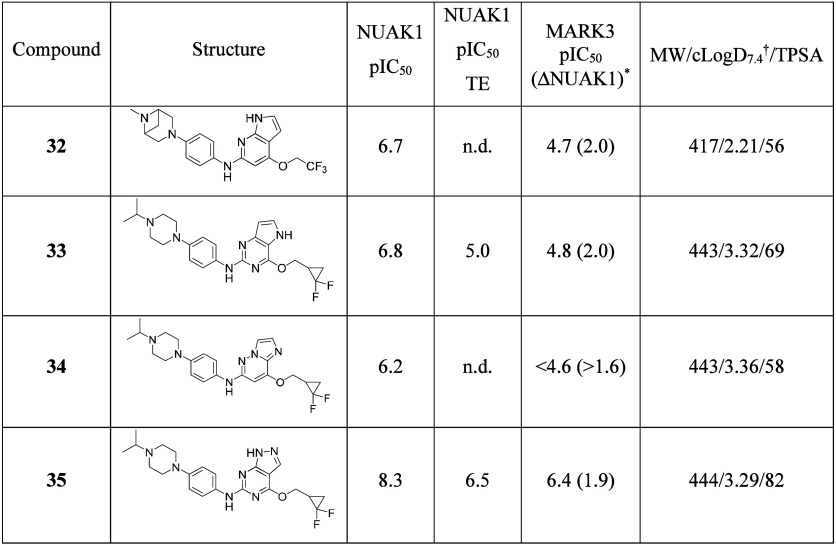
Effect of Core Changes on NUAK1 and MARK3 Potency^a^

a NUAK1: ADP-Glo assay; NUAK1 cell TE: cell-based NanoBRET target engagement assay; *ΔNUAK1: NUAK1 pIC_50_ minus MARK3 pIC_50_; †cLogD_7.4_ calculated using SimulationsPlus ADMET Predictor

As with MARK3, pyrrolopyrimidine analogues were less selective than purine analogues toward NUAK1 over MARK1, MARK2 and MARK4 (see Table S2). Within this subseries, both cyclopropyl analogues (**30** and **23**) were the most selective and all compounds tested were less active toward the other MARK kinases compared to MARK3.

In general, compounds in this series appeared to be marginally more selective over NUAK2 than those containing a purine core, especially iso-propylpiperazine analogues **23** and **25** (Table S3, both >25-fold selective).

Significantly, none of the compounds tested in the panel of CDK kinases appeared to substantially inhibit CDK2 (Table 8). It is striking to note that while the difluorocyclopropyl methyl analogues **25** and **31** showed significant activity toward both CDK4 and CDK6, the cyclopropyl analogue **23** was more selective. The triazole analogue **29** was highly selective toward NUAK1 over all three CDKs.

**Table 8:** CDK Kinase/Cyclin Activity Data, Single Point at 1 μM

Compound	NUAK1 pIC_50_	CDK2/A^a^ % inhibition at 1 μM	CDK4/D1^b^ % inhibition at 1 μM	CDK6/D1^b^ % inhibition at 1 μM
**23**	8.7	7	39	26
**25**	8.7	31	81	68
**29**	8.7	8	18	2
**31**	8.5	31	70	49

a Z′-LYTE screening assay.

b Adapta screening assay.

Compound **23** was tested in a panel of 140 protein kinases (Table S4). While these results suggested NUAK1 was the major target for this compound, **23** inhibited 23 additional kinases with <20% activity of the control, potentially due to the smaller size of the cyclopropyl ring allowing for greater promiscuity.

A comparison of ADME properties showed reduced mouse microsomal stability in the pyrrolopyrimidine series compared to purine analogues (see Table 9). However, compound **23** demonstrated higher permeability and a significantly lower rate of efflux compared to its purine analogue **19**, which was encouraging for potential CNS penetration. Protein binding was high in this subseries, but this was reduced with the triazole-containing analogue **29**. This compound also showed an excellent microsomal half-life of 159 min. The diazaspiroheptane analogue **31** had a moderate half-life of 37.7 min with a slightly higher brain free fraction than **23**.

**Table 9:** ADME Properties of Pyrrolopyrimidine Analogues

Compound	MLM *t*_1/2_ (min)	MDCK-MDR1 permeability P_app_ (cms^–1^) (ER)	PPB F_u_	BPB F_u_
**23**	22.8	4.5 (1.5)	0.007	0.002
**24**	30.3	n.d.	n.d.	0.006
**25**	25.8	n.d.	n.d.	n.d.
**26**	8.3	n.d.	n.d.	n.d.
**27**	53.4	n.d.	n.d.	n.d.
**28**	31.7	n.d.	n.d.	0.007
**29**	159	0.67 (54)	0.158	0.055
**30**	16.3	n.d.	n.d.	n.d.
**31**	37.7	4.62 (22)	0.018	0.008

Interestingly, replacement of the iso-propylpiperazine with 6-methyl-3,6-diazabicyclo[3.1.1]­heptane resulted in less metabolically stable compounds (compare **23** and **29** with **30** and **27** respectively), which is in contrast to previous observations where this alteration improved stability.^27^

Compounds **23**, **29** and **31** were selected for an in vivo PK study (5 mg/kg, I.P. cassette, see Table 10). Of the 3 compounds studied, **29** demonstrated the longest plasma half-life of 1.5 h, which was lower than the microsomal stability predicted, but negligible brain exposure. **31** showed a similar plasma exposure to **29** but enhanced brain penetration (K_p,uu,b_ 0.51 (2 h)). Compound **23** showed the highest brain to plasma ratio of the compounds screened with significant brain exposure at 2 h (K_p,uu,b_ 2.29). In addition to **20**, **29** offers promising properties as a peripheral in vivo tool compound, and we suggest **23** (ARUK2010489) as a probe for the study of NUAK1 inhibition in the CNS.

**Table 10:** Pharmacokinetic Data for Pyrrolopyrimidine Analogues (Mouse, 5 mg/kg, I.P.)

Parameter	Compound **23**	Compound **29**	Compound **31**
Plasma *t*_1/2_ (hr)	1.2	1.5	1.2
AUC_inf_ (hr.ng/mL)	988	1698	1775
[Brain] at 30 min (ng/mL)	3917	54	405
[Brain] at 2 h (ng/mL)	1068	27	414
Brain/Plasma (30 min/2 h)	7.6/9.4	0.1/0.2	0.6/1.1
K_p,uu,b_ (30 min/2 h)	2.06/2.29	0.02/0.06	0.27/0.51

In summary, mining the literature for NUAK1 inhibitors identified the CDK2 inhibitor NU6140 as a potential starting point for developing a selective, brain penetrant NUAK1 inhibitor. Initial ADME studies suggested this compound was metabolically liable in mice and not predicted to be CNS penetrant. Optimisation of **5** resulted in compounds that are highly potent toward NUAK1 both in a biochemical assay and an assay of cellular target engagement and highly selective over the CDK kinases and a panel of 140 protein kinases. In vivo plasma half-lives have been significantly increased, and compounds such as ARUK2010694 (**20**) are potent and selective tool molecules suitable for peripheral in vivo studies. In contrast, replacement of the purine with a pyrrolpyrimidine core has provided highly brain penetrant NUAK1 inhibitors, including ARUK2010489 (**23**), suitable for in vivo CNS studies.

## Supplementary Material



## Notes

Additional assay data, biological assay procedures, in vivo experiment details, synthesis and characterization of all compounds (PDF)

## Notes

No unexpected safety issues were noted from this work. Novel compounds were assumed toxic as a precaution.

## Abbreviations

ADME: Absorption, distribution, metabolism and excretion
ADP: Adenosine diphosphate
AMP: Adenosine monophosphate
ATP: Adenosine triphosphate
AUC: Area under the curve
BPB: Brain protein binding
BQL: Below quantifiable limit
CNS: Central nervous system
ER: Efflux ratio
F_u_: Fraction unbound
K_m_: Michaelis constant
K_p,uu,b_: Unbound partition coefficient (brain to plasma)
MDCK-MDR1: Madin-Darby canine kidney-multidrug resistance mutation 1
MLM: Mouse liver microsomes
MW: Molecular weight
ND: Not determined
P_app_: Apparent permeability coefficient
PDB: Protein database
PPB: Plasma protein binding
TE: Target engagement
TPSA: Total polar surface area
