# Supplementary material for: Development of Purine and Pyrrolopyrimidine Scaffolds as Potent, Selective, and Brain Penetrant NUAK1 Inhibitors
Source: ACS Med Chem Lett. 2025 Sep 10;16(11):2264–72. doi: 10.1021/acsmedchemlett.5c00467 (PMC12621045; doi:10.1021/acsmedchemlett.5c00467)
Supplement: Supplementary file 1 [file ml5c00467_si_001.pdf]

Development of Purine and Pyrrolopyrimidine Scaffolds as Potent, Selective and Brain Penetrant NUAK1 Inhibitors

Gregory G. Aldred, Helen K. Boffey*, Henriette M. G. Willems, David Winpenny, Helen Scott, Jonathan H. Clarke, Stephen P. Andrews, John Skidmore*

Corresponding authors

Helen Boffey; Email: hkb32@cam.ac.uk

John Skidmore; Email: js930@cam.ac.uk

Affiliations

The ALBORADA Drug Discovery Institute, University of Cambridge, Island Research Building, Cambridge Biomedical Campus, Hills Road, Cambridge, CB2 0AH, United Kingdom

Supplementary Information

Table of Contents

	Page
Abbreviations	2
Supplementary Tables	3
Table S1: Reported NUAK1/2 inhibitors with in vivo data	3
Table S2: MARK kinase activity data	8
Table S3: NUAK2 kinase activity data	9
Table S4: Kinase panel data	10
Biological experimental procedures	15
ADMET and PK experimental methods	17
Homology modelling and docking	18
Synthetic chemistry methods	18
Synthetic schemes	19
Table 1 compounds	20
Table 2 compounds	26
Table 6 compounds	32
Table 7 compounds	45
NMR spectra and LC-MS traces of selected compounds	52
References	66

Abbreviations

ADMET: Absorption, distribution, metabolism, excretion and toxicity, aq: aqueous solution, BPB: Brain protein binding, dba: dibenzylideneacetone, DCE: 1,2-dichloroethane, DCM: dichloromethane, eq: equivalents, DMSO: dimethylsulfoxide, ER: efflux ratio, ESI: electrospray ionisation, F_u: Fraction unbound, HLM: Human liver microsomes, HPLC: high-performance liquid chromatography, HRMS: High resolution mass spectrometry, h: hours, IPA: iso-propanol, MDCK-MDR1: Madin-Darby canine kidney-multidrug resistance mutation 1, min: minutes, MS: mass spectrometry, MLM: Mouse liver microsomes, NMR: nuclear magnetic resonance, P_{app}: Apparent permeability coefficient, PPB: Plasma protein binding, *R*-BINAP: (*R*)-(+)-2,2′-Bis(diphenylphosphino)-1,1′-binaphthalene, RMSD: root mean square deviation, rt: room temperature, sat.: saturated, SCX: strong cation exchange, UPLC: ultra high-performance liquid chromatography. XantPhos: (9,9-dimethyl-9*H*-xanthene-4,5-diyl)bis(diphenylphosphane).

Supplementary table

Table S1. Reported NUAK1/2 inhibitors with in vivo data

ChEMBL ID	Name	NUAK1 ChEMBL pIC_{50}	NUAK1 ADP-Glo pIC_{50} *	Primary target (pIC_{50})	# targets with higher potency	Compared with	CL ($mL.min^{1}.kg^{-1}$)	CL species and route	AUC $ng.hr.mL^{-1}$	AUC dose, species and route	Reference
CHEMBL509032 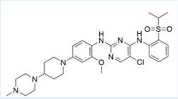	NVP-TAE684	8.92		ULK1 (9.1) BMPR1B (9.1)	9	NUAK2	17.1	mouse i.v.	14430	10 mg/kg mouse p.o.	10.1016/j.bmcl.2012.01.084; 10.1021/acs.jmedchem.0c00533
CHEMBL3884319 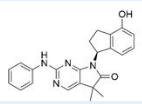	No name	8.3		MARK3 (8.9) FLT3 (8.1)	1	NUAK1	341	rat i.v.			10.1016/j.bmcl.2016.08.068
CHEMBL482967 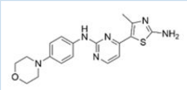	CYC-116	8.15		AAK1 (8.1) BMP2K (8.1) CDK17 (8.1)	3	NUAK2			5011	10 mg/kg rat p.o.	10.1021/jm901913s; 10.1126/science.aan4368
CHEMBL3326006 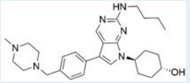	UNC2025	8.1	7.76	MERTK (8.9) FLT3 (8.8)	6	NUAK1	9.2	mouse i.v.	4385	3 mg/kg mouse i.v.	10.1021/jm500749d; 10.1021/acs.jmedchem.8b01229
CHEMBL4569508 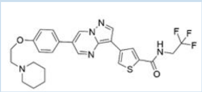	No name	7.82		MARK3 (9.0) CDK2 (7.9)	4	NUAK1	32	rat i.v.			10.1016/j.bmcl.2016.02.003

ChEMBL ID	Name	NUAK1 ChEMBL pIC_{50}	NUAK1 ADP-Glo pIC_{50} *	Primary target (pIC_{50})	# targets with higher potency	Compared with	CL ($mL.min^{1}.kg^{-1}$)	CL species and route	AUC $ng.hr.mL^{-1}$	AUC dose, species and route	Reference
CHEMBL4550702<br>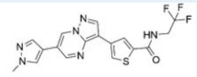	No name	7.72	NUAK1 (7.66) MARK3 (8.51)	MARK3 (8.2) DYRK1A (8.0)	3	NUAK1	7.4	rat i.v.			10.1016/j.bmcl.2016.02.003
CHEMBL502835<br>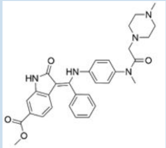	Nintedanib	7.68		BMP2K (8.7) FLT3 (8.4)	34	NUAK2	173	mouse i.v.	2746	50 mg/kg mouse p.o.	10.1021/jm900431g; 10.1038/nbt.1990
CHEMBL402548<br>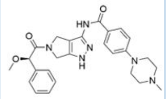	Danusertib	7.6		AURKA (8.2) ABL1 (7.9)	2	NUAK2	60	mouse i.v.	3085	10 mg/kg mouse i.v.	10.1021/jm060897w;
CHEMBL5193702<br>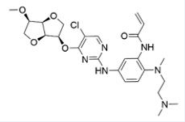	No name	7.6		JAK3 (8.5)	1	NUAK1			537	5 mg/kg rat i.v.	10.1021/acs.jmedchem.2c00922
CHEMBL191003<br>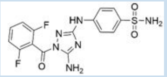	JNJ-7706621	7.58	7.65	CDK2 (8.7) CDK1 (8.2)	7	NUAK2			4496	3 mg/kg rat p.o.	10.1016/j.bmcl.2006.04.071; 10.1038/nbt1358
CHEMBL2064666<br>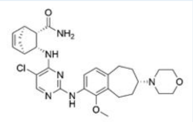	No name	7.58		ALK (8.2) RPS6KA2 (8.2)	4	NUAK1	40	dog i.v.	38187	100 mg/kg rat p.o.	10.1021/jm201550q

ChEMBL ID	Name	NUAK1 ChEMBL pIC_{50}	NUAK1 ADP-Glo pIC_{50} *	Primary target (pIC_{50})	# targets with higher potency	Compared with	CL $(mL.min^{1}.kg^{-1})$	CL species and route	AUC $ng.hr.mL^{-1}$	AUC dose, species and route	Reference
CHEMBL3545311 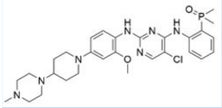	Brigatinib	7.33		ROS1 (8.7)<br>FLT3 (8.7)	5	NUAK1	7.7	rat i.v.	2268	3 mg/kg<br>rat i.v.	10.1021/acs.jmedchem.6b00306;
CHEMBL535 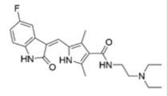	Sunitinib	7.32	7.32	FLT1 (8.5)<br>STK17A (8.5)	53	NUAK1	13	rat i.v.	23994	15 mg/kg<br>rat p.o.	10.1124/dmd.111.042853;
CHEMBL494089 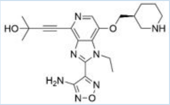	GSK-690693	7.07		PRKCH (8.7)<br>PRKG2 (8.5)	19	NUAK2	56.7	rat i.v.	3250	10.6 mg/kg<br>rat i.v.	10.1021/jm8004527; 10.1038/nbt.1990

* Data generated at ALBORADA Drug Discovery Institute using ADP-Glo assay

Method for extraction of Table S1 data

ChEMBL33 was mined for compounds with NUAK (1 or 2) activity with pChEMBL value > 6.5 (see NUAK query.sql below) using a local copy of ChEMBL33 in a postgres16 installation.

The CHEMBL identifiers of the extracted NUAK ligands were used in a second search, where ChEMBL33 was searched for compounds in the NUAK set that have clearance (standard_type = CL) or AUC data (see below for 'in_vivo_query.sql'). 16 compounds were identified that have either clearance or AUC data or both, and a NUAK pIC50 > 7 and were not staurosporin analogues. For these 16 compounds, ChEMBL33 was searched for all target data available in nM or % inhibition format. The resulting data file (app 7100 rows) was processed in Knime to aggregate this data to 1 row per compound.

NUAK_ query.sql

```
select distinct md.chembl_id as compound_chembl_id, act.molregno, mh.parent_molregno,
cs.canonical_smiles, act.standard_type, act.standard_relation, act.standard_value,
act.standard_units, act.pchembl_value, act.activity_comment, act.data_validity_comment,
td.pref_name, td.chembl_id as target_chembl_id, ass.assay_type, ass.description,
compsyn.component_synonym, cs.standard_inchi_key, ass.confidence_score, ass.assay_organism,
docs.doi, docs.pubmed_id

from activities act, assays ass, target_dictionary td, compound_structures cs, docs,
molecule_hierarchy mh, component_synonyms compsyn, molecule_dictionary md,
target_components tc, compound_records cr, protein_classification pc, component_class cc,
component_sequences cse

where act.molregno=cr.molregno and cr.molregno=md.molregno and md.molregno=mh.molregno
and md.molregno=cs.molregno and act.assay_id=ass.assay_id and ass.tid=td.tid and tc.tid=td.tid and
compsyn.component_id=tc.component_id and docs.doc_id=act.doc_id and pc.protein_class_id =
cc.protein_class_id and cse.component_id = tc.component_id and cc.component_id =
cse.component_id and

compsyn.syn_type='GENE_SYMBOL' and (act.standard_units = 'nM' OR act.standard_units = '%') and
td.organism in ('Rattus norvegicus', 'Homo sapiens', 'Mus musculus')  and
compsyn.component_synonym like 'NUAK%' and act.pchembl_value > 7
```

In vivo query.sql

```
select distinct md.chembl_id as compound_chembl_id, act.molregno, mh.parent_molregno,
cs.canonical_smiles, act.standard_type, act.standard_relation, act.standard_value,
act.standard_units, act.pchembl_value, act.activity_comment, act.data_validity_comment,
ass.assay_type, ass.description, cs.standard_inchi_key, ass.confidence_score, ass.assay_organism,
docs.doi, docs.pubmed_id
from activities act, assays ass, compound_structures cs, docs, molecule_hierarchy mh,
molecule_dictionary md, compound_records cr
where act.molregno=cr.molregno and cr.molregno=md.molregno and md.molregno=mh.molregno
and md.molregno=cs.molregno and act.assay_id=ass.assay_id
and docs.doc_id=act.doc_id
and act.standard_type like 'CL'
and md.chembl_id in ('CHEMBL1229592','CHEMBL1287853')
```

The same search was carried out with ‘AUC’ instead of ‘CL’. Only 2 CHEMBL identifiers are shown for illustration. Add a comma-separated list of identifiers of interest.

To retrieve all data for the 16 compounds with in vivo data, replace the last 3 lines of the NUAK_query.sql (above) with the lines below, adding the relevant CHEMBL identifiers:

```
and compsyn.syn_type='GENE_SYMBOL' and (act.standard_units = 'nM' OR act.standard_units = '%')
and td.organism in ('Rattus norvegicus', 'Homo sapiens', 'Mus musculus')  and md.chembl_id in
('CHEMBL1892019', 'CHEMBL9387', 'CHEMBL112', 'CHEMBL72365’);
```

Table S2. MARK kinase activity data (ADP-Glo assay)

Compound	NUAK1 pIC_{50}	MARK1 pIC_{50} (ΔNUAK1)*	MARK2 pIC_{50} (ΔNUAK1)*	MARK3 pIC_{50} (ΔNUAK1)*	MARK4 pIC_{50} (ΔNUAK1)*
**5**	8.6	5.6 (3.0)	5.3 (3.3)	6 (2.6)	5.7 (2.9)
**6**	9	6.1 (2.9)	6.0 (3.0)	6.2 (2.8)	6.0 (3.0)
**7**	8.8	5.8 (3.0)	5.8 (3.0)	6.4 (2.4)	5.7 (3.1)
**9**	8.8	5.9 (2.9)	5.8 (3.0)	6.1 (2.7)	5.7 (3.1)
**12**	8.7	7.3 (1.4)	7.0 (1.7)	6.6 (2.1)	7.3 (1.4)
**13**	8.8	6.5 (2.3)	6.4 (2.4)	6.9 (1.9)	6.3 (2.5)
**14**	8.9	6.4 (2.5)	6.3 (2.6)	6.8 (2.1)	6.2 (2.7)
**19**	8.8	5.9 (2.9)	5.9 (2.9)	6.7 (2.1)	6.4 (2.4)
**20**	8.7	6.0 (2.7)	5.8 (2.9)	6.2 (2.5)	5.5 (3.2)
**23**	8.7	6.2 (2.5)	6.1 (2.6)	7.1 (1.6)	6.1 (2.6)
**24**	8.6	6.3 (2.3)	6.3 (2.3)	6.7 (1.9)	6.1 (2.5)
**25**	8.7	6.4 (2.3)	6.5 (2.2)	7.1 (1.6)	6.8 (1.9)
**26**	8.4	6.3 (2.1)	6.4 (2.0)	6.8 (1.6)	6.2 (2.2)
**27**	8.1	5.9 (2.2)	5.7 (2.4)	6.3 (1.8)	5.7 (2.4)
**29**	8.7	6.5 (2.2)	6.5 (2.2)	6.9 (1.8)	6.3 (2.4)
**30**	8.7	3.1 (5.6)	6.1 (2.6)	6.5 (2.2)	6.0 (2.7)
**31**	8.5	6.5 (2.0)	6.5 (2.0)	7.0 (1.5)	6.3 (2.2)

*ΔNUAK1: NUAK1 pIC_{50} minus MARK pIC_{50}

Table S3. NUAK2 kinase activity data (ADP-Glo assay)

**Compound**	**NUAK1 pIC_{50}**	**NUAK2 pIC_{50} (ΔNUAK1)***
**5**	8.6	8.3 (0.3)
**8**	8.7	8.5 (0.2)
**12**	8.7	8.3 (0.4)
**13**	8.8	8.9 (-0.1)
**14**	8.9	9.0 (-0.1)
**18**	8.5	7.8 (0.7)
**19**	8.8	8.5 (0.3)
**20**	8.7	7.6 (0.6)
**21**	8.3	7.7 (0.6)
**23**	8.7	7.3 (1.4)
**25**	8.7	7.2 (1.5)
**26**	8.4	7.2 (1.2)
**31**	8.5	7.7 (0.8)
**35**	8.3	7.2 (1.3)

*ΔNUAK1: NUAK1 pIC_{50} minus NUAK2 pIC_{50}

Table S4. Kinase selectivity screening for **20** and **23** at 1 μM against a general kinase panel of 140 targets in radiometric filter binding assay using ^{33}P-γ–ATP at the MRC PPU International Centre for Kinase Profiling, University of Dundee. pIC_{50} values were determined for kinases with <20% activity remaining at 1 μM, for compound **20** only.

	**Compound 20**			**Compound 23**		
**Kinase**	**% activity remaining**	**s.d.**	**pIC_{50}**	**% activity remaining**	**s.d.**	**pIC_{50}**
ABL	34	1		10	0	
AMPK (hum)	25	1		6	1	6.5*
ASK1	120	0		59	10	
Aurora A	40	1		31	4	
Aurora B	90	12		20	2	
BRK	58	13		51	3	
BRSK1	75	10		108	11	
BRSK2	117	8		90	0	
BTK	68	7		40	1	
CAMK1	95	13		51	8	
CAMKKb	70	4		36	4	
CDK2-Cyclin A	88	5		37	2	
CDK9-Cyclin T1	94	1		110	10	
CHK1	55	0		22	5	
CHK2	73	0		75	8	
CK1γ2	49	6		16	4	
CK1δ	101	0		24	5	
CK2	116	2		50	2	
CLK2	34	5		20	2	
CSK	82	2		97	9	
DAPK1	69	7		18	0	
DDR2	29	1		39	6	
DYRK1A	92	0		53	3	
DYRK2	106	10		65	5	
DYRK3	103	13		61	6	
EF2K	101	5		113	4	
EIF2AK3	115	12		39	2	

EPH-A2	88	5		87	13	
EPH-A4	109	4		126	15	
EPH-B1	73	2		102	4	
EPH-B2	73	5		106	7	
EPH-B3	88	10		89	13	
EPH-B4	72	7		93	18	
ERK1	97	2		104	0	
ERK2	98	9		93	9	
ERK5	93	3		82	7	
ERK8	32	4		52	0	
FGF-R1	58	0		17	3	
GCK	61	1		34	8	
GSK3b	84	0		84	4	
HER4	46	3		51	7	
HIPK1	95	14		63	6	
HIPK2	50	0		22	2	
HIPK3	115	8		98	1	
IGF-1R	109	6		6	0	
IKKb	97	7		89	5	
IKKe	49	1		69	10	
IR	77	2		25	11	
IRAK1	78	1		75	1	
IRAK4	100	13		67	9	
IRR	83	8		48	14	
JAK3	22	4		23	7	
JNK1	38	3		23	4	
JNK2	55	4		34	6	
JNK3	51	8		58	10	
Lck	54	9		72	9	
LKB1	96	1		90	3	
MAP4K3	71	5		11	0	
MAP4K5	50	0		11	0	
MAPKAP-K2	102	11		104	7	
MAPKAP-K3	81	8		50	5	
MARK1	45	7	6.0*	16	1	6.2*
MARK2	44	6	5.8*	23	0	6.1*

MARK3	46	10	6.2*	6	1	7.1*
MARK4	59	9	5.5*	26	6	6.1*
MEKK1	79	9		88	14	
MELK	24	1		27	4	
MINK1	71	1		23	1	
MKK1	73	14		95	10	
MKK2	88	2		99	15	
MKK6	106	9		119	2	
MLK1	18	2	6.9	65	9	
MLK3	33	10		92	1	
MNK1	101	5		106	8	
MNK2	80	3		83	5	
MPSK1	97	3		77	6	
MSK1	80	4		69	6	
MST2	71	6		45	15	
MST3	111	4		56	14	
MST4	55	12		111	7	
NEK2a	78	6		73	2	
NEK6	103	3		85	19	
**NUAK1**	6	1	8.7*	3	1	8.7*
OSR1	82	4		44	7	
p38a MAPK	101	3		109	5	
p38b MAPK	82	8		111	6	
p38d MAPK	74	6		91	9	
p38g MAPK	97	1		97	2	
PAK2	90	4		95	15	
PAK4	52	6		8	2	
PAK5	59	4		34	6	
PAK6	89	15		56	6	
PDGFRA	37	2		96	6	
PDK1	42	2		66	2	
PHK	45	1		16	0	
PIM1	85	1		94	12	
PIM2	107	3		103	10	
PIM3	104	5		113	7	
PINK	78	2		126	8	

PKA	92	1		86	6	
PKBa	97	6		87	1	
PKBb	102	6		99	9	
PKCa	109	14		108	4	
PKCz	106	5		123	17	
PKCγ	88	7		81	1	
PKD1	31	3		6	0	
PLK1	107	14		94	13	
PRAK	98	6		108	10	
PRK2	57	8		43	0	
RIPK2	48	12		77	7	
ROCK 2	36	4		25	1	
RSK1	82	2		12	2	
RSK2	96	4		37	1	
S6K1	90	0		51	9	
SGK1	126	10		113	6	
SIK2	19	0	6.9	11	1	
SIK3	64	7		80	4	
SmMLCK	91	4		37	9	
Src	25	3		15	1	
SRPK1	114	5		103	1	
STK33	96	2		51	7	
SYK	59	11		9	3	
TAK1	17	2	6.6	4	1	
TAO1	94	3		46	2	
TBK1	27	5		46	7	
TESK1	45	1		68	4	
TGFBR1	95	2		114	4	
TIE2	78	7		84	6	
TLK1	83	0		120	17	
TrkA	44	1		29	4	
TSSK1	24	2		14	1	
TTBK1	96	1		96	18	
TTBK2	103	4		105	14	
TTK	55	1		83	1	
ULK1	21	3		16	2	

ULK2	13	5	7.7	21	1	
VEG-FR	112	13		7	2	
WNK1	90	7		77	4	
YES1	7	0	7.5	4	5	
ZAP70	95	0		78	4	

* Data generated at ALBORADA Drug Discovery Institute using ADP-Glo assay

Biological experimental procedures

ADP-Glo biochemical assays

Protein kinase activity in the presence of inhibitor compounds was determined by ADP-Glo assay (Promega), as previously described.[1,2] Compounds were serialised using a Labcyte Echo 520 and transferred to 384 well plates (Greiner 784201) followed by the addition 5 µL of substrate containing peptide and ATP at K_m in buffer (see **Table S5**). 5 µL of purified protein was added (ng/well; see **Table S5**) and the plate was incubated for 60 minutes at room temperature. 3 µL of assay mixture was transferred using an Integra Viaflo384 into a white low volume plate (Greiner 781904) prior to the addition of 3 µL of ADP-Glo Reagent™ for a further 40-minute incubation. After incubation with 6 µL of Kinase Detection Reagent for 30 minutes, plate luminescence was read (Pherastar FSX, BMG Labtech).

Buffer A

33 mM HEPES pH7.4, 0.1% CHAPS, 20 mM $MgCl_2$ and 16.7 µM EGTA

Buffer B

30 mM HEPES pH7.4, 3 mM $MgCl_2$, 3 mM $MnCl_2$, 1.2 mM DTT, 0.01% CHAPS

Table S5. Conditions for protein kinase ADP-Glo assays

Protein	ATP conc.	ATP Km	Peptide name	Peptide conc.	Peptide supplier	Enzyme amount /well	Enzyme supplier	Assay vol.	Buffer	Min. n[a]
NUAK1	25 µM	31 µM	Sakamototide	50 µM	Merck	6.7 ng	MRC PPU	10 µL	A	6
MARK1	20 µM	ND	CHKTide	100 µM	MRC PPU	1.2 ng	MRC PPU	10 µL	A	2
MARK2	20 µM	ND	CHKTide	100 µM	MRC PPU	4 ng	MRC PPU	10 µL	A	2
MARK3	10 µM	ND	CHKTide	200 µM	MRC PPU	2.65 ng	MRC PPU	10 µL	A	6
MARK4	50 µM	ND	CHKTide	100 µM	MRC PPU	2.65 ng	MRC PPU	10 µL	A	2
NUAK2	20 µM	21 µM	Sakamototide	100 µM	Merck	1.15 ng	MRC PPU	3 µL	A	2

[a]Min. n: Minimum number of technical replicates per compound

NanoBRET target engagement assay

The binding of compounds to NUAK1 in intact cells was assessed using an NanoBRET^{TE} Intracellular Kinase Assay (Promega N2501). HEK293 cells transiently transfected with NanoLuc-NUAK1 Fusion Vector (Promega NV1831) were incubated with 10 nL K5 tracer (provided in the kit, Promega N2501) and 40 nL of the test compounds in 100% DMSO in a white low volume non-binding 384 assay plate (Grenier Bio One 784904) for 90 min at 37 °C. After incubation, followed by cooling for 15 min at room temperature, 4 µL of the Intracellular TE Nano-Glo Substrate/Inhibitor (Promega N2160) was added to each well. The plate was read using a Pherastar FSX plate reader (BMG Labtech). Minimum number of 2 technical replicates per compound.

Data Analysis:

Activity pIC_{50} values and standard error of the mean (SEM) were estimated using a 4-parameter fit (Dotmatics). The SEM for all reported data was 0.2 or lower. See Experimental Procedures for minimum number of replicates per compound. Each experiment included a Standard compound whose pIC_{50} value was compared to a quality control chart. If the Standard pIC_{50} value fell outside the range of pIC_{50} +/-3 standard deviation, then the experiment was invalidated.

CDK2/cyclin A: Z′-LYTE™ screening assay performed by Thermo Fisher Scientific: The 2X CDK2/cyclin A / Ser/Thr 12 mixture is prepared in 50 mM HEPES pH 7.5, 0.01% BRIJ-35, 10 mM $MgCl_2$, 1 mM EGTA. The final 10 µL Kinase Reaction consists of 1.22 - 10.3 ng CDK2/cyclin A and 2 µM Ser/Thr 12 in 50 mM HEPES pH 7.5, 0.01% BRIJ-35, 10 mM $MgCl_2$, 1 mM EGTA. After the 1 hour Kinase Reaction incubation, 5 µL of a 1:4096 dilution of Development Reagent A is added.

CDK4/cyclin D1: Adapta™ screening assay was performed by Thermo Fisher Scientific. The 2X CDK4/cyclin D1 / Rb Substrate mixture is prepared in 50 mM HEPES pH 7.5, 0.01% BRIJ-35, 10 mM $MnCl_2$, 1 mM EGTA, 2 mM DTT, 0.02% NaN_3. The final 10 µL Kinase Reaction consists of 7.5 - 30 ng CDK4/cyclin D1 and 1 µM Rb Substrate in 32.5 mM HEPES pH 7.5, 0.005% BRIJ-35, 5 mM $MnCl_2$, 0.5 mM EGTA, 1 mM DTT, 0.01% NaN3. After the 1 hour Kinase Reaction incubation, 5 µL of Detection Mix is added.

CDK6/cyclin D1: Adapta™ screening assay was performed by Thermo Fisher Scientific. The 2X CDK6/cyclin D1 / Rb Substrate mixture is prepared in 50 mM HEPES pH 7.5, 0.01% BRIJ-35, 10 mM $MnCl_2$, 1 mM EGTA, 2 mM DTT, 0.02% NaN3. The final 10 µL Kinase Reaction consists of 1.75 - 7 ng CDK6/cyclin D1 and 1 µM Rb Substrate in 32.5 mM HEPES pH 7.5, 0.005% BRIJ-35, 5 mM $MnCl_2$, 0.5 mM EGTA, 1 mM DTT, 0.01% NaN_3. After the 1 hour Kinase Reaction incubation, 5 µL of

Detection Mix is added.

All single point assay determinations carried out in duplicate.

ADMET and PK experimental methods

Microsomal stability: analysis was performed by Cyprotex Discovery. Briefly, test compounds in DMSO were incubated at a concentration of 1 µM (0.25% DMSO final) with mouse hepatic microsomes (0.5 mg protein/mL) in the presence of NADPH (1 mM) at 37 °C. Aliquots were taken at time intervals (0, 5, 15, 30 and 45 min) and stopped by transferring into acetonitrile, then analysed using generic LC-MS/MS conditions for compound remaining, allowing the determination of the half-life for the compound.

MDR1-MDCK Permeability (bi-directional): was performed by Cyprotex Discovery. Briefly, compounds were administered at 10 µM (1% DMSO final) to the apical or basolateral side of a confluent monolayer of MDR1- MDCK cells, then incubated at 37 °C for 60 minutes before appearance on the opposite side of the monolayer was determined LC-MS/MS. The efflux ratio (ER) is calculated from the ratio of B-A to A-B permeabilities.

Plasma protein binding: was performed by ChemPartner Co. Ltd. Briefly, compounds in DMSO (1 µM, 0.2% DMSO final) were added to mouse plasma or brain tissue and dialysed against buffer for 5 hrs at 37 °C. The compound concentration in each of the plasma and buffer compartments was determined by mass spectrometry and used to calculate the percentage compound bound.

Pharmacokinetic analysis: was performed by ChemPartner Co. Ltd. Male CD-1 mice (N=3 per timepoint) were intraperitoneally dosed at 5 mg/kg, as a cassette of five compounds, formulated in 10% DMSO, 10% Solutol HS 15, 80% (50 mM citrate buffer pH3.0) at 0.5 mg/mL. At the designated time points a blood sample was collected from the facial vein into K2EDTA tubes, and plasma separated by centrifugation. Mice were euthanised by CO_2 inhalation and brains were dissected and homogenised. Compound levels were quantified by LC-MS/MS and PK parameters estimated by a non-compartmental model using WinNonlin 8.2.

Homology modelling and docking

A BLAST search with the kinase domain sequence of human NUAK1 (residues 55-306) identified MARK3 as a protein with a homologous kinase domain for which a crystal structure was available. MOE (www.ccg.com) was used to align the NUAK1 kinase domain to human MARK3 structure pdb:7P1L. 25 homology models were built, based on this alignment, sampling 3 side chain conformations and including the 7P1L ligand. The final model was minimized to an RMSD gradient of 0.5. NU6140 was docked flexibly to this homology model with GOLD (version 2020.2.0). An

interaction motif was used to guide the docking. Two interactions out of Glu79O, Ala81O and Ala81N were required. Docking settings were default, except that ring flip was switched on and only 1 solution was collected. The PLP scoring function was used. The 71PL ligand was used to define the binding site. The top scoring pose was selected. This was further minimized in a flexible binding site in MOE (with QuickPrep, AMBER:EHT forcefield, binding site not tethered, atoms further than 8Å from binding site fixed), resulting in the pose shown in **Figure 2** of the main manuscript.

Synthetic chemistry methods

All screening compounds are >95% pure by HPLC analysis.

Reagents and solvents were of commercially available reagent grade quality and used without further purification. **5** (NU6140) was purchased from Cambridge Bioscience (CAY17271), Sigma Aldrich (SML1323) or Tocris (3301), **7** (NU6102) was purchased from Insight Biotechnology (SC-222082), **10** (NU2058) was purchased from Cambridge BioScience (CAY21415). 2-Chloro-6-(cyclohexylmethoxy)-9*H*-purine, 4-(4-isopropylpiperazin-1-yl)aniline and 4-(6-methyl-3,6-diazabicyclo[3.1.1]heptan-3-yl)aniline were prepared as previously described.[1,2] Reactions requiring anhydrous conditions were carried out in oven dried glassware under an atmosphere of N_2. Reactions were monitored by thin-layer chromatography (TLC) on silica gel 60 F_{254} aluminium or glass supported sheets, or by liquid chromatography-mass spectrometry (LC-MS). Flash column chromatography was carried out on a Biotage Isolera One system using normal phase (SiO_2) or reverse phase (C18) cartridges. Compounds were loaded in solution or adsorbed onto Celite® 545 or ISOLUTE® HM-N and eluted using a linear gradient of the specified solvents. Purification by C18 reverse phase HPLC was carried using an Agilent 1260 Infinity machine and a Waters XBridge BEH C18 OBD column (130 Å, 5 µm, 30 mm × 100 mm) with a linear gradient of H_2O (with 0.1% NH_3) and MeCN (with 0.1% NH_3). LCMS analysis was performed on a Waters Aquity HClass UPLC system with a Aquity QDa for mass detection. High-resolution mass spectra (HRMS) were measured on a Waters Vion IMS QTof spectrometer. NMR spectra were recorded on a Bruker Avance III (^{1}H = 300 MHz, ^{19}F = 282 MHz), a Bruker Avance III (^{1}H = 400 MHz) or a Bruker Avance III with Dual ^{13}C/^{1}H Cryoprobe (^{1}H = 500 MHz, ^{13}C = 126 MHz) spectrometer using the requisite solvent as a reference for internal deuterium lock. The chemical shift data for each signal are given as δ chemical shift (multiplicity, *J* values in Hz, integration) in units of parts per million (ppm) relative to tetramethylsilane (TMS) where δH (TMS) = 0.00 ppm. The multiplicity of each signal is indicated by: s (singlet), d (doublet), t (triplet), q (quartet), quint (quintet), sex (sextet), or m (multiplet). Signals from exchangeable protons are not always detected. UPLC analysis of final compounds was performed on a Waters Aquity HClass UPLC system (methods

A-E) and HPLC analysis was performed on an Agilent 1260, infinity-II HPLC system (method F), data is reported as method name, retention time, UV % purity. The method parameters are as follows:

Method	Column	Additive	Flow rate	Gradient (time, %MeCN in H_2O)
A	BEH C18 (130 Å, 1.7 µm, 2.1 mm × 50 mm)	1 mM NH_3	0.6 mL/min	0 min, 5%; 0.8 min, 5%; 3.3 min, 95%; 4.3 min, 95%; 4.5 min, 5%; 5.5 min, 5%.
B	HSS C18 (100 Å, 1.8 µm, 2.1 mm × 50 mm)	0.1% HCO_2H	0.6 mL/min	0 min, 5%; 0.8 min, 5%; 3.3 min, 95%; 4.3 min, 95%; 4.5 min, 5%; 5.5 min, 5%.
C	BEH C18 (130 Å, 1.7 µm, 2.1 mm × 50 mm)	1 mM NH_3	0.6 mL/min	0 min, 5%; 0.8 min, 5%; 8.3 min, 95%; 9.3 min, 95%; 9.5 min, 5%; 10.5 min, 5%.
D	HSS C18 (100 Å, 1.8 µm, 2.1 mm × 50 mm)	0.1% HCO_2H	0.6 mL/min	0 min, 5%; 0.8 min, 5%; 8.3 min, 95%; 9.3 min, 95%; 9.5 min, 5%; 10.5 min, 5%.
E	C18 (1. 9 µm, 2.1 mm × 50 mm)	0.1% HCO_2H	0.6 - 1 mL/ min	0 min, 3%; 0.2 min, 3%; 2.7 min, 98%; 3.0 min, 100%; 3.5 min, 100%; 3.51 min, 3%; 4.0 min, 3%.
F	Sunfire C18 (3.5 µm, 4.6 mm × 150 mm)	0.1% TFA	1 mL/min	0 min, 10%; 7 min, 90%; 9 min, 100%; 14 min, 100%; 14.01 min, 10%; 17 min, 10%.

Synthetic schemes

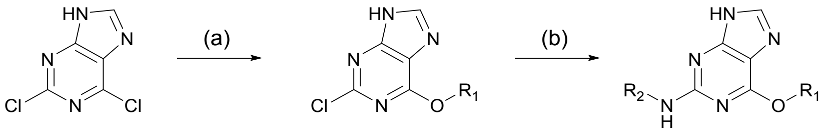

Scheme 1: *Reagents and conditions*: (a) R_1OH, NaH, THF, 0 °C, 16 h. (b) R_2NH_2, TFA, *sec*-BuOH, 140 °C, µW.

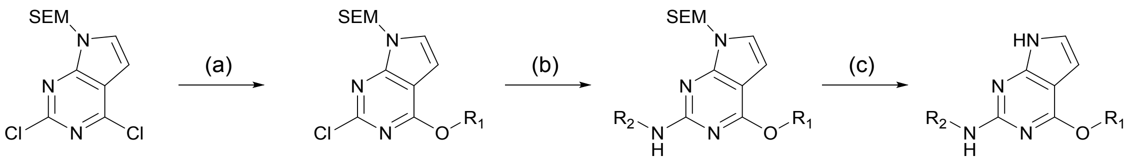

36

Scheme 2: *Reagents and conditions*: (a) R_1OH, NaH, THF, 0 °C, 16 h. (b) R_2NH_2, $Pd(dba)_2$•$CHCl_3$, Xantphos, Cs_2CO_3, 1,4-dioxane, 120 °C, 16 h. (c) TBAF, 1,2-ethylenediamine, 1,4-dioxane, 60 °C, 16 h.

General procedure A:

Requisite purine (1 eq) and aniline (1.5 eq) were sealed in a MW vial and taken up in 2-butanol (0.2 M) and trifluoroacetic acid (1 eq). The reaction mixture was then heated at 140 °C in the microwave for the indicated time. The reaction was quenched with sat. aq $NaHCO_3$ and extracted with EtOAc (×3). The combined organic layers were dried (Na_2SO_4), filtered and concentrated under reduced pressure. The crude material was then purified by the stated method to yield title compound.

General procedure B:

To a stirred solution of requisite aniline and chloro-pyrrolopyrimidine in 1,4-dioxane (0.1 M) was added Cs_2CO_3 (3 eq) and the reaction mixture purged with N_2 for 15 min, followed by addition of $Pd(dba)_2$ (0.4 eq) and Xantphos (0.4 eq). The reaction mixture was stirred at 120 °C for 16 h before cooling to RT and pouring into water. The aqueous layer was extracted with EtOAc (×2) and the combined organic layers were dried (Na_2SO_4), filtered and concentrated under reduced pressure. The crude material was then purified by silica gel flash column chromatography to yield title compound.

General procedure C:

To a cooled (0 °C) and stirred solution of (trimethylsilyl)ethoxy)methyl protected compound in 1,4-dioxane (0.035 M) was added 1,2-ethyenediamine (5 eq) and TBAF (1.0 M solution in THF, 5 eq). Following addition, the reaction was heated to 60 °C for 16 h. The reaction was then cooled, quenched with water and extracted with Et_2O (×3). The combined organic layers were washed with sat. aq NH_4Cl (×3), dried (Na_2SO_4), filtered and concentrated under reduced pressure. The crude material was then purified by reverse phase column chromatography (0.1% NH_3 in MeCN/Water; 30-90%) to yield title compound.

Table 1 Compounds

4-((6-(Cyclohexylmethoxy)-9*H*-purin-2-yl)amino)benzamide (6)

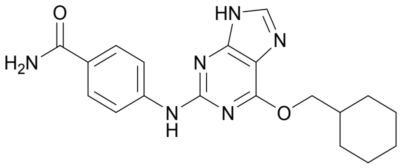

2-Chloro-6-(cyclohexylmethoxy)-9*H*-purine (60.0 mg, 0.220 mmol) and 4-aminobenzamide (61.3 mg, 0.450 mmol) were reacted according to general procedure A for 6 h. Initial purification by silica gel flash column chromatography (EtOAc/petroleum ether; 5-100%) was followed by further purification by preparatory HPLC (0.1% NH_3 in MeCN/Water; 5-95%) to yield 4-((6-(cyclohexylmethoxy)-9*H*-purin-2-yl)amino)benzamide **(6)** (5.0 mg, 0.014 mmol, 6% yield) as a white lyophilised solid. MS (ESI+) *m/z* calcd for $C_{19}H_{23}N_6O_2^+$ $[M + H]^+$ 367.2, found 367.4. UPLC analysis (method D), 4.18 min,

>98% purity. ^{1}H NMR (300 MHz, DMSO-d_6) δ 9.59 (s, 1H), 8.04 (s, 1H), 7.92 – 7.84 (m, 2H), 7.83 – 7.75 (m, 3H), 7.14 (s, 1H), 4.35 (d, *J* = 6.3 Hz, 2H), 1.93 – 1.61 (m, 6H), 1.36 – 1.00 (m, 6H).

(4-((6-(Cyclohexylmethoxy)-9*H*-purin-2-yl)amino)phenyl)(morpholino)methanone (8)

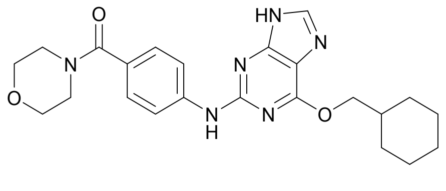

2-Chloro-6-(cyclohexylmethoxy)-9*H*-purine (100 mg, 0.190 mmol) and (4-aminophenyl)morpholin-4-yl-methanone (58.0 mg, 0.280 mmol) were reacted according to general procedure A for 1 h. The crude material was purified by preparatory HPLC (0.1% NH_3 in MeCN/Water; 5-95%) to yield (4-((6-(cyclohexylmethoxy)-9*H*-purin-2-yl)amino)phenyl)(morpholino)methanone **(8)** (30 mg, 0.070 mmol, 37% yield). MS (ESI+) *m/z* calcd for $C_{23}H_{29}N_6O^+$ $[M + H]^+$ 437.2, found 437.4. UPLC analysis (method C), 4.63 min, 96% purity. ^{1}H NMR (300 MHz, $CDCl_3$) δ 7.81 (s, 1H), 7.69 (d, *J* = 8.6 Hz, 2H), 7.48 (br s, 1H), 7.42 (d, *J* = 8.5 Hz, 2H), 4.32 (d, *J* = 6.2 Hz, 2H), 3.71 (s, 8H), 1.94 – 1.81 (m, 3H), 1.80 – 1.62 (m, 3H), 1.36 – 1.18 (m, 3H), 1.17 – 0.98 (m, 2H). *Purine N-H unresolved.*

6-(Cyclohexylmethoxy)-*N*-(3-(4-methylpiperazin-1-yl)phenyl)-9*H*-purin-2-amine (9)

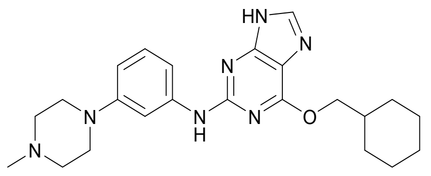

2-Chloro-6-(cyclohexylmethoxy)-9*H*-purine (100 mg, 0.190 mmol) and 3-(4-methylpiperazin-1-yl)anilin (53.8 mg, 0.280 mmol) were reacted according to general procedure A for 1 h. The crude material was purified by preparatory HPLC (0.1% NH_3 in MeCN/Water; 5-95%) to yield 6-(cyclohexyl-methoxy)-*N*-[3-(4-methylpiperazin-1-yl)phenyl]-9*H*-purin-2-amine **(9)** (15 mg, 0.036 mmol, 19% yield). MS (ESI+) *m/z* calcd for $C_{23}H_{32}N_7O^+$ $[M + H]^+$ 422.2, found 422.5. UPLC analysis (method C), 5.78 min, >98% purity. ^{1}H NMR (300 MHz, $CDCl_3$) δ 7.24 – 7.11 (m, 3H), 7.04 – 6.97 (m, 2H), 6.69 – 6.61 (m, 1H), 4.32 (d, *J* = 6.2 Hz, 2H), 3.18 (t, *J* = 5.0 Hz, 4H), 2.51 (t, *J* = 5.0 Hz, 4H), 2.32 (s, 3H), 1.97 – 1.81 (m, 3H), 1.78 – 1.62 (m, 3H), 1.36 – 1.17 (m, 3H), 1.17 – 0.98 (m, 2H). *Purine N-H unresolved.*

6-(Cyclohexylmethoxy)-*N*-(3-methoxy-1-methyl-1*H*-pyrazol-4-yl)-9*H*-purin-2-amine (11)

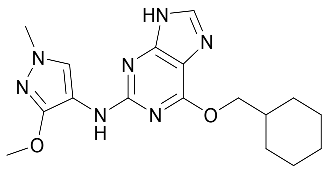

2-Chloro-6-(cyclohexylmethoxy)-9*H*-purine (100 mg, 0.190 mmol) and 3-methoxy-1-methyl-1*H*-pyrazole-4-amine hydrochloride (46.0 mg, 0.280 mmol) were reacted according to general procedure A for 1 h. The crude material was purified by preparatory HPLC (0.1% NH_3 in MeCN/Water; 5-95%) to yield 6-(cyclohexylmethoxy)-*N*-(3-methoxy-1-methyl-1*H*-pyrazol-4-yl)-9*H*-purin-2-amine **(11)** (19.6 mg, 0.055 mmol, 29% yield). MS (ESI+) *m/z* calcd for $C_{17}H_{24}N_7O_2^+$ $[M + H]^+$ 358.2, found 358.4. UPLC analysis (method D), 4.33 min, >98% purity. ^1H NMR (300 MHz, $CDCl_3$) δ 10.93 (s, 1H), 7.63 (s, 1H), 7.47 (s, 1H), 6.41 (s, 1H), 4.31 (d, *J* = 6.3 Hz, 2H), 3.96 (s, 3H), 3.71 (s, 3H), 1.95 – 1.86 (m, 3H), 1.80 – 1.70 (m, 3H), 1.33 – 1.21 (m, 3H), 1.15 – 0.99 (m, 2H).

6-(Cyclohexylmethoxy)-*N*-(4-(4-methylpiperazin-1-yl)phenyl)-9*H*-purin-2-amine (12)

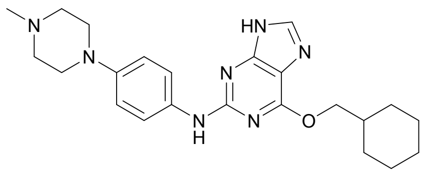

2-Chloro-6-(cyclohexylmethoxy)-9*H*-purine (50.0 mg, 0.190 mmol) and 4-(4-methylpiperazino)aniline (26.9 mg, 0.141 mmol) were reacted according to general procedure A for 1 h. The crude material was purified by preparatory HPLC (0.1% NH_3 in MeCN/Water; 5-95%) to yield 6-(cyclohexylmethoxy)-*N*-(4-(4-methylpiperazin-1-yl)phenyl)-9*H*-purin-2-amine **(12)** (14 mg, 0.033 mmol, 35%) as a white solid. MS (ESI+) *m/z* calcd for $C_{23}H_{32}N_7O^+$ $[M + H]^+$ 422.3, found 422.4. UPLC analysis (method D), 3.76 min, >98% purity. ^1H NMR (300 MHz, $CDCl_3$) δ 7.50 (s, 1H), 7.41 (d, *J* = 8.9 Hz, 2H), 6.88 (d, *J* = 8.9 Hz, 2H), 6.70 (s, 1H), 4.25 (d, *J* = 6.3 Hz, 2H), 3.17 – 3.05 (m, 4H), 2.58 – 2.45 (m, 4H), 2.30 (s, 3H), 1.89 – 1.80 (m, 3H), 1.74 – 1.62 (m, 3H), 1.27 – 1.14 (m, 3H), 0.79 – 0.70 (m, 2H). *Purine N-H unresolved.*

6-(Cyclohexylmethoxy)-*N*-(3-(4-methylpiperazin-1-yl)phenyl)-9*H*-purin-2-amine (13)

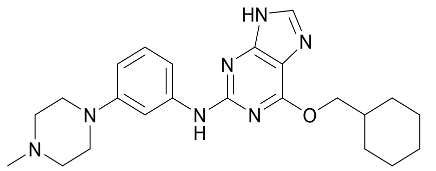

2-Chloro-6-(cyclohexylmethoxy)-9*H*-purine (100 mg, 0.190 mmol) and 3-(4-methylpiperazin-1-yl)anilin (53.8 mg, 0.280 mmol) were reacted according to general procedure A for 1 h. The crude material was purified by preparatory HPLC (0.1% NH_3 in MeCN/Water; 5-95%) to yield 6-(cyclohexyl-methoxy)-*N*-[3-(4-methylpiperazin-1-yl)phenyl]-9*H*-purin-2-amine **(13)** (15 mg, 0.036 mol, 19% yield). MS (ESI+) *m/z* calcd for $C_{23}H_{32}N_7O^+$ $[M + H]^+$ 422.2, found 422.5. UPLC analysis (method C), 5.78 min, > 98% purity. ^{1}H NMR (300 MHz, $CDCl_3$) δ 7.24 – 7.11 (m, 3H), 7.04 – 6.97 (m, 2H), 6.69 – 6.61 (m, 1H), 4.32 (d, *J* = 6.2 Hz, 2H), 3.18 (t, *J* = 5.0 Hz, 4H), 2.51 (t, *J* = 5.0 Hz, 4H), 2.32 (s, 3H), 1.97 – 1.81 (m, 3H), 1.78 – 1.62 (m, 3H), 1.36 – 1.17 (m, 3H), 1.17 – 0.98 (m, 2H). *Purine N-H unresolved.*

6-(Cyclohexylmethoxy)-*N*-(4-(4-isopropylpiperazin-1-yl)phenyl)-9*H*-purin-2-amine (14)

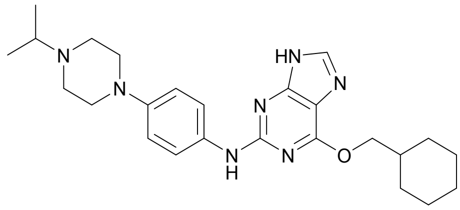

2-Chloro-6-(cyclohexylmethoxy)-7-methyl-purine (50 mg, 0.094 mmol) and 4-(4-isopropyl-piperazin-1-yl)aniline (26.9 mg, 0.120 mmol) were reacted according to general procedure A for 5 h. The crude material was purified by preparatory HPLC (0.1% NH_3 in MeCN/Water; 5-95%) to yield 6-(cyclohexylmethoxy)-*N*-(4-(4-isopropylpiperazin-1-yl)phenyl)-9*H*-purin-2-amine **(14)** (20 mg, 0.044 mmol, 47 %) as a white solid. MS (ESI+) *m/z* calcd for $C_{25}H_{36}N_7O^+$ $[M + H]^+$ 450.3, found 450.0. UPLC analysis (method D), 3.80 min, >98% purity. ^{1}H NMR (300 MHz, DMSO-d_6) δ 12.69 (s, 1H), 8.99 (s, 1H), 7.91 (s, 1H), 7.60 (d, *J* = 8.6 Hz, 2H), 6.85 (d, *J* = 9.1 Hz, 2H), 4.29 (d, *J* = 6.3 Hz, 2H), 3.09 – 2.96 (m, 4H), 2.65 (p, *J* = 6.5 Hz, 1H), 2.60 – 2.53 (m, 4H), 1.96 – 1.77 (m, 3H), 1.77 – 1.59 (m, 3H), 1.36 – 1.16 (m, 3H), 1.16 – 1.03 (m, 2H), 1.00 (d, *J* = 6.5 Hz, 6H).

2-Chloro-6-(cyclohexylmethoxy)-7-methyl-7*H*-purine (37)

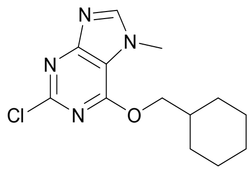

To a stirred suspension of sodium hydride (98.5 mg, 2.46 mmol) in THF (10 mL) at 0 °C was added a solution of cyclohexylmethanol (169 mg, 1.48 mmol) in THF (2.0 mL) and the reaction mixture stirred for 5 min until gas evolution ceased. A solution of 2,6-dichloro-7-methyl-7*H*-purine (200 mg, 0.990 mol) in THF (15 mL) was then added and the reaction mixture stirred at 0 °C, for 10 min, before warming to RT for 4 h. After this time the reaction was quenched with sat. aq $NaHCO_3$ and diluted with EtOAc. The organic layer was separated, dried (Na_2SO_4), filtered and concentrated under reduced pressure to yield 2-chloro-6-(cyclohexylmethoxy)-7-methyl-7*H*-purine **(37)** (250 mg, 0.890 mmol, 90% yield). MS (ESI+) *m/z* calcd for $C_{13}H_{18}ClN_4O^+$ $[M + H]^+$ 281.1, found 281.2. UPLC analysis (method A), 3.20 min, >98% purity. ^1H NMR (300 MHz, $CDCl_3$) δ 7.97 (s, 1H), 4.41 (d, *J* = 6.1 Hz, 2H), 4.05 (s, 3H), 1.98 – 1.70 (m, 6H), 1.42 – 1.06 (m, 4H), 0.96 – 0.80 (m, 1H).

6-(Cyclohexylmethoxy)-7-methyl-*N*-(4-(4-methylpiperazin-1-yl)phenyl)-7*H*-purin-2-amine (15)

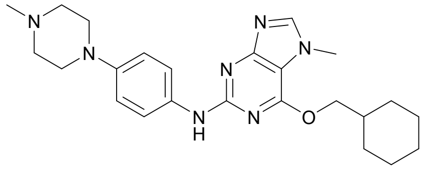

2-Chloro-6-(cyclohexylmethoxy)-7-methyl-7*H*-purine **(37)** (30.0 mg, 0.110 mmol) and 4-(4-methyl-piperazino)aniline (30.7 mg, 0.160 mmol) were reacted according to general procedure A for 3 h. The crude material was partial purified by silica gel flash column chromatography (MeOH/DCM; 0-20%) followed by further purification by preparatory HPLC (0.1% NH_3 in MeCN/Water; 5-95%) to yield 6-(cyclohexylmethoxy)-7-methyl-*N*-(4-(4-methylpiperazin-1-yl)phenyl)-7*H*-purin-2-amine **(15)** (5.0 mg, 0.011 mmol, 11%) as a white solid. MS (ESI+) *m/z* calcd for $C_{24}H_{34}N_7O^+$ $[M + H]^+$ 436.3, found 436.1. UPLC analysis (method D), 4.69 min, >95% purity. ^1H NMR (300 MHz, CDCl3) δ 7.75 (s, 1H), 7.65 (d, *J* = 9.0 Hz, 2H), 7.08 (s, 1H), 6.91 (d, *J* = 9.0 Hz, 2H), 4.30 (d, *J* = 6.0 Hz, 2H), 3.95 (s, 3H), 3.40 (s, 4H), 3.29 (s, 4H), 2.83 (s, 3H), 1.91 – 1.80 (m, 3H), 1.79 – 1.67 (m, 3H), 1.36 – 1.21 (m, 3H), 1.19 – 1.07 (m, 2H).

2-Chloro-6-(cyclohexylmethoxy)-9-methyl-9*H*-purine (38)

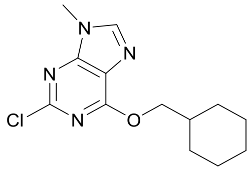

To a stirred suspension of sodium hydride (98.5 mg, 2.46 mmol) in THF (10 mL) at 0 °C was added a solution of cyclohexylmethanol (169 mg, 1.48 mmol) in THF (2.0 mL) and the reaction mixture stirred for 5 min until gas evolution ceased. A solution of 2,6-dichloro-9-methyl-9*H*-purine (200 mg, 0.990 mmol) in THF (15 mL) was then added and the reaction stirred at 0 °C for 10 min, before warming to RT for 4 h. After this time the reaction mixture was quenched with sat. aq $NaHCO_3$ and diluted with EtOAc. The organic layer was separated, dried (Na_2SO_4), filtered and concentrated under reduced pressure to yield 2-chloro-6-(cyclohexylmethoxy)-9-methyl-9*H*-purine **(38)** (200 mg, 0.712 mmol, 72%). Material carried forward without further purification. MS (ESI+) *m/z* calcd for $C_{13}H_{18}ClN_4O^+$ $[M + H]^+$ 281.1, found 281.2. UPLC analysis (method A), 3.30 min, 73% purity. ^{1}H NMR (300 MHz, $CDCl_3$) δ 7.85 (s, 1H), 4.40 (d, *J* = 6.2 Hz, 2H), 3.85 (s, 3H), 1.99 – 1.84 (m, 3H), 1.81 – 1.66 (m, 3H), 1.37 – 1.02 (m, 4H), 0.92 – 0.80 (m, 1H).

6-(Cyclohexylmethoxy)-9-methyl-*N*-(4-(4-methylpiperazin-1-yl)phenyl)-9*H*-purin-2-amine (16)

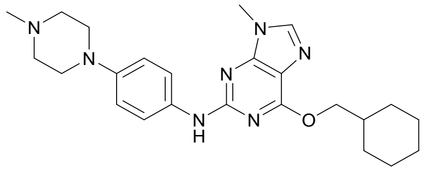

2-Chloro-6-(cyclohexylmethoxy)-9-methyl-9*H*-purine **(38)** (30.0 mg, 0.110 mmol) and 4-(4-methyl-piperazino)aniline (30.7 mg, 0.160 mmol) were reacted according to general procedure A for 3 h. The crude material was purified by preparatory HPLC (0.1% NH_3 in MeCN/Water; 30-70%) to yield 6-(cyclohexylmethoxy)-9-methyl-*N*-(4-(4-methylpiperazin-1-yl)phenyl)-9*H*-purin-2-amine **(16)** (5.0 mg, 0.011 mmol, 11%) as a white solid. MS (ESI+) *m/z* calcd for $C_{24}H_{34}N_7O^+$ $[M + H]^+$ 436.3, found 436.1. UPLC analysis (method D), 4.31 min, >95% purity. ^{1}H NMR (300 MHz, $CDCl_3$) δ 7.64 – 7.54 (m, 3H), 6.95 (d, *J* = 9.0 Hz, 2H), 6.86 (s, 1H), 4.32 (d, *J* = 6.3 Hz, 2H), 3.74 (s, 3H), 3.24 (t, *J* = 5.0 Hz, 4H), 2.72 (br s, 4H), 2.44 (s, 3H), 1.98 – 1.86 (m, 3H), 1.81 – 1.69 (m, 3H), 1.36 – 1.19 (m, 3H), 1.16 – 1.01 (m, 2H).

Table 2 Compounds

***N,N*-Diethyl-4-((6-(2-methoxyethoxy)-9*H*-purin-2-yl)amino)benzamide (17)**

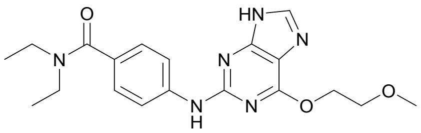

2-Chloro-6-(2-methoxyethoxy)-9*H*-purine (50.0 mg, 0.220 mmol) and 4-amino-*N,N*-diethylbenzamide (100 mg, 0.600 mmol) were reacted according to general procedure A for 4 h. The crude material was purified by preparatory HPLC (0.1% NH_3 in MeCN/Water; 10-50%) to yield *N,N*-diethyl-4-((6-(2-methoxyethoxy)-9*H*-purin-2-yl)amino)benzamide **(17)** (34 mg, 0.088 mmol, 40%) as a white solid. MS (ESI+) *m/z* calcd for $C_{19}H_{25}N_6O_3^+$ $[M + H]^+$ 385.2, found 385.4. UPLC analysis (method D), 3.61 min, >98% purity. ^{1}H NMR (300 MHz, $CDCl_3$) δ 7.82 (s, 1H), 7.65 (d, *J* = 8.5 Hz, 2H), 7.49 (s, 1H), 7.37 (d, *J* = 8.5 Hz, 2H), 4.64 (t, *J* = 4.8 Hz, 2H), 3.81 (t, *J* = 4.8 Hz, 2H), 3.66 – 3.28 (m, 4H), 3.44 (s, 3H), 1.20 (s, 6H). *Purine N-H unresolved.*

2-Chloro-6-(2,2,2-trifluoroethoxy)-9*H*-purine (39)

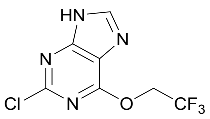

To a stirred suspension sodium hydride (132 mg, 3.31 mmol) in THF (5.0 mL) at 0 °C was added a solution of 2,2,2-trifluoroethanol (0.14 mL, 1.98 mmol) in THF (3.0 mL) until gas evolution ceased. A solution of 2,6-dichloropurine (250 mg, 1.32 mmol) in THF (5.0 mL) was then added and the reaction mixture stirred at 0 °C for 10 min, before warming to RT and stirring for 16 h. After this time the reaction was quenched with water and diluted with EtOAc. The organic layer was separated and the aqueous layer extracted with EtAOc (×2) before the combined organic extracts were washed with brine, dried (Na_2SO_4), filtered and concentrated under reduced pressure to yield 2-chloro-6-(2,2,2-trifluoroethoxy)-9*H*-purine **(39)** (372 mg, 1.29 mmol, 98% yield). MS (ESI+) *m/z* calcd for $C_7H_5ClF_3N_4O^+$ $[M + H]^+$ 253.0, found 253.1. UPLC analysis (method B), 2.50 min, 95% purity. ^{1}H NMR (300 MHz, DMSO-d_6) δ 8.17 (s, 1H), 5.22 (q, *J* = 9.0 Hz, 2H). *Purine N-H unresolved.* ^{19}F NMR (282 MHz, DMSO-d_6) δ –72.24 (s).

***N*-(4-(4-Isopropylpiperazin-1-yl)phenyl)-6-(2,2,2-trifluoroethoxy)-9*H*-purin-2-amine (18)**

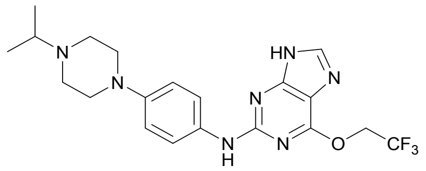

2-Chloro-6-(2,2,2-trifluoroethoxy)-9*H*-purine **(39)** (50.0 mg, 0.100 mmol) and 4-(4-isopropyl-piperazin-1-yl)aniline (32.6 mg, 0.150 mmol) were reacted according to general procedure A for 3 h. The crude material was placed on an SCX-II cartridge, washed with DCM and MeOH before eluting with methanolic ammonia. The basic fraction was concentrated under reduced pressure and purified by preparatory HPLC (0.1% NH_3 in MeCN/Water; 30-70%) to yield *N*-(4-(4-isopropylpiperazin-1-yl)phenyl)-6-(2,2,2-trifluoroethoxy)-9*H*-purin-2-amine **(18)** (8.0 mg, 0.018 mmol, 19%) as a white solid. MS (ESI+) *m/z* calcd for $C_{20}H_{25}F_3N_7O^+$ $[M + H]^+$ 436.2, found 436.2. UPLC analysis (method D), 3.14 min, 95% purity. ^1H NMR (300 MHz, DMSO-d_6) δ 9.19 (s, 1H), 8.03 (s, 1H), 7.57 (d, J = 8.9 Hz, 2H), 6.88 (d, J = 9.0 Hz, 2H), 5.21 (q, J = 9.1 Hz, 2H), 3.08 – 3.01 (m, 4H), 2.65 (q, J = 6.5 Hz, 1H), 2.61 – 2.53 (m, 4H), 1.00 (d, J = 6.5 Hz, 6H). *Purine N-H unresolved.* ^{19}F NMR (282 MHz, DMSO-d_6) δ –72.22 (s).

2,6-Dichloro-9-((2-(trimethylsilyl)ethoxy)methyl)-9*H*-purine (40a) and 2,6-dichloro-7-((2-(trimethylsilyl)ethoxy)methyl)-7*H*-purine (40b)

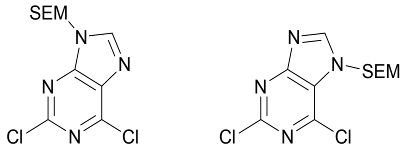

To a cooled (0 °C) and stirred solution of 2,6-dichloropurine (200 mg, 1.06 mmol) in THF (10.5 mL) was added sodium hydride (50.8 mg, 2.12 mmol) and the reaction mixture stirred for 10 min. After this time 2-(chloromethoxyethyl)trimethylsilane (211 mg, 1.27 mmol) was added and the reaction stirred at RT for 24 h. The reaction mixture was diluted with EtAOc and the organic phase washed with water, followed by brine, dried (Na_2SO_4), filtered and concentrated under reduced pressure. The crude material was purified by silica gel flash column chromatography (EtOAc/petroleum ether; 0-100%) and carried forward as a mixture of SEM protected regioisomers **(40a and 40b)** (268 mg, 0.840 mmol, 79% yield). MS (ESI+) *m/z* calcd for $C_{11}H_{17}Cl_2N_4OSi^+$ $[M + H]^+$ 319.0, found 319.0. UPLC analysis (method A), 3.29 min (minor isomer, 33%), 3.39 min (major isomer, 67%).

2-Chloro-6-(cyclopropylmethoxy)-9-((2-(trimethylsilyl)ethoxy)methyl)-9*H*-purine (41a) and 2-chloro-6-(cyclopropylmethoxy)-7-((2-(trimethylsilyl)ethoxy)methyl)-7*H*-purine (41b)

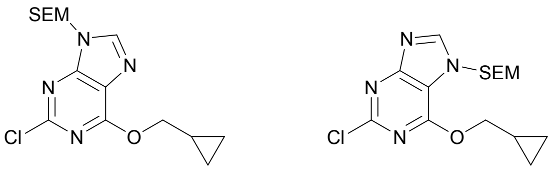

To a cooled (0 °C) and stirred solution of cyclopropylmethanol (88.1 mg, 1.22 mmol) in THF (8.0 mL) was added sodium hydride (58.6 mg, 2.44 mmol) and the suspension stirred for 10 min. A mixture of SEM protected 2,6-dichloropurine isomers (260 mg, 0.810 mmol) in THF (1.0 mL) was then added and the reaction mixture stirred at RT for 30 min. After this time the reaction was quenched with the addition of water, extracted with EtOAc and the organic phase separated. The organic phase was washed with brine, dried (Na_2SO_4), filtered and concentrated under reduced pressure. The crude material was purified by silica gel flash column chromatography (EtOAc/petroleum ether; 2-80%) to yield a mixture of 2-chloro-6-(cyclopropylmethoxy)-9-((2-(trimethylsilyl)ethoxy)methyl)-9*H*-purine **(41a)** and 2-chloro-6-(cyclopropylmethoxy)-7-((2-(trimethylsilyl)ethoxy)methyl)-7*H*-purine **(41b)** (170 mg, 0.479 mmol, 59% yield) as a colourless solid. MS (ESI+) *m/z* calcd for $C_{15}H_{24}ClN_4O_2Si^+$ $[M + H]^+$ 355.1, found 355.1. UPLC analysis (method A), 3.60 min, >95% purity. (No clear splitting on LCMS)

6-(Cyclopropylmethoxy)-*N*-(4-(4-isopropylpiperazin-1-yl)phenyl)-9-((2-(trimethylsilyl)ethoxy)methyl)-9*H*-purin-2-amine (42)

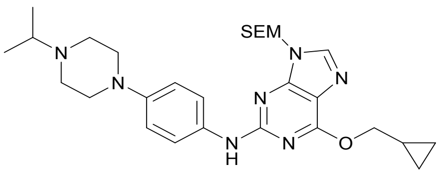

A mixture of 2-chloro-6-(cyclopropylmethoxy)-9-((2-(trimethylsilyl)ethoxy)methyl)-9*H*-purine and 2-chloro-6-(cyclopropylmethoxy)-7-((2-(trimethylsilyl)ethoxy)methyl)-7*H*-purine (160 mg, 0.450 mmol) and 4-(4-isopropylpiperazin-1-yl)aniline (124 mg, 0.564 mmol) were reacted together according to general procedure B in the MW at 120 °C for 1 h. The reaction mixture was placed on an SCX-II cartridge, washed with DCM and methanol before eluting with methanolic ammonia. The basic fraction was concentrated under reduced pressure and purified by silica gel flash column chromatography (MeOH/DCM; 0-10%) to yield 6-(Cyclopropylmethoxy)-*N*-(4-(4-isopropylpiperazin-1-yl)phenyl)-9-((2-(trimethylsilyl)ethoxy)methyl)-9*H*-purin-2-amine **(42)** (110 mg, 0.205 mmol, 45% yield) as an orange oil. MS (ESI+) *m/z* calcd for $C_{28}H_{44}F_3N_7O_2Si^+$ $[M + H]^+$ 537.3, found 537.5. UPLC analysis (method D), 3.07 min, 95% purity. ^1H NMR (300 MHz, $CDCl_3$) δ 7.77 (s, 1H), 7.58 (d, *J* = 8.8 Hz, 2H), 6.94 (d, *J* = 9.0 Hz, 2H), 6.88 (s, 1H), 5.50 (s, 2H), 4.37 (d, *J* = 7.3 Hz, 2H), 3.85 – 3.68 (m, 4H), 3.65

– 3.57 (m, 2H), 1.91 – 1.78 (m, 4H), 1.50 – 1.11 (m, 8H), 0.98 – 0.89 (m, 2H), 0.70 – 0.57 (m, 2H), 0.46 – 0.36 (m, 2H), –0.06 (s, 9H).

6-(Cyclopropylmethoxy)-*N*-(4-(4-isopropylpiperazin-1-yl)phenyl)-9*H*-purin-2-amine (19)

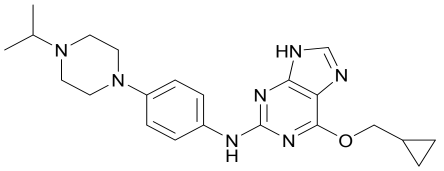

6-(Cyclopropylmethoxy)-*N*-(4-(4-isopropylpiperazin-1-yl)phenyl)-9-((2-(trimethylsilyl)ethoxy) methyl)-9*H*-purin-2-amine (**42)** (104 mg, 0.190 mmol) was reacted according to general procedure C. The reaction mixture was then placed on an SCX-II cartridge, washed with DCM and methanol before eluting with methanolic ammonia. The basic fraction was concentrated under reduced pressure and purified by preparatory HPLC (0.1% NH_3 in MeCN/Water; 30-70%) followed by further purification by silica gel flash column chromatography (MeOH/DCM; 0-20%). Resultant material was taken up in EtOAc and washed with aq NH_4Cl, the organic layer dried (Na_2SO_4), filtered and concentrated under reduced pressure to yield 6-(cyclopropylmethoxy)-*N*-(4-(4-isopropylpiperazin-1-yl)phenyl)-9*H*-purin-2-amine **(19)** (26 mg, 0.064 mmol, 33%) as a white solid. MS (ESI+) *m/z* calcd for $C_{22}H_{30}N_7O^+$ $[M + H]^+$ 408.2, found 408.4. UPLC analysis (method D), 2.19 min, >98% purity. ^1H NMR (300 MHz, DMSO-d_6) δ 12.69 (s, 1H), 9.00 (s, 1H), 7.91 (s, 1H), 7.59 (d, *J* = 9.0 Hz, 2H), 6.86 (d, *J* = 9.1 Hz, 2H), 4.32 (d, *J* = 7.3 Hz, 2H), 3.06 – 2.99 (m, 4H), 2.72 – 2.61 (m, 1H), 2.61 – 2.55 (m, 4H), 1.41 – 1.29 (m, 1H), 1.01 (d, *J* = 6.5 Hz, 6H), 0.64 – 0.54 (m, 2H), 0.44 – 0.32 (m, 2H).

2-Chloro-6-((2,2-dimethyltetrahydro-2*H*-pyran-4-yl)methoxy)-9*H*-purine (43)

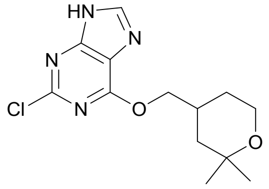

To a stirred solution of (2,2-dimethyltetrahydro-2*H*-pyran-4-yl)methanol (57.2 mg, 0.397 mmol) in THF (0.66 mL) was added sodium hydride (25.0 mg, 1.06 mmol) and the reaction mixture stirred for 5 min. After this time a solution of 2,6-dichloropurine (50.0 mg, 0.265 mmol) in THF (0.66 mL) was added and the reaction mixture stirred for 16 h at RT. The reaction was then quenched with the addition of water and extracted with EtOAc (×2). The organic layer was washed with brine, dried (Na_2SO_4), filtered and concentrated under reduced pressure to yield 2-chloro-6-((2,2-dimethyltetrahydro-2*H*-

pyran-4-yl)methoxy)-9*H*-purine **(43)** (78.0 mg, 0.263 mmol, 99% yield). Material was carried forward without further purification. MS (ESI+) *m/z* calcd for $C_{13}H_{18}ClN_4O_2^+$ $[M + H]^+$ 297.1, found 297.0. UPLC analysis (method B), 2.58 min, >98% purity.

6-((2,2-Dimethyltetrahydro-2*H*-pyran-4-yl)methoxy)-*N*-(4-(4-isopropylpiperazin-1-yl)phenyl)-9*H*-purin-2-amine (20)

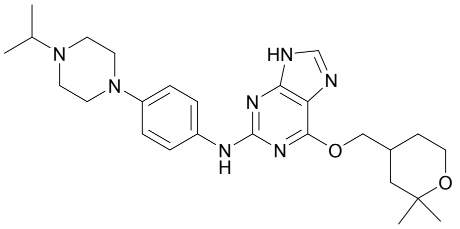

2-Chloro-6-((2,2-dimethyltetrahydro-2*H*-pyran-4-yl)methoxy)-9*H*-purine **(43)** (78.0 mg, 0.130 mmol) and 4-(4-isopropylpiperazin-1-yl)aniline (34.6 mg, 0.160 mmol) were reacted according to general procedure A for 3.5 h. The crude material was initially purified by preparatory HPLC (0.1% NH_3 in MeCN/Water; 30-70%) to yield the desired product as the TFA salt. The salt was taken up in DCM, placed on an SCX-II column and eluted with methanolic ammonia to desalt and yield 6-((2,2-dimethyltetrahydro-2*H*-pyran-4-yl)methoxy)-*N*-(4-(4-isopropylpiperazin-1-yl)phenyl)-9*H*-purin-2-amine **(20)** (46 mg, 0.096 mmol, 73%) as a white solid. MS (ESI+) *m/z* calcd for $C_{26}H_{38}N_7O_2^+$ $[M + H]^+$ 480.3, found 480.3. UPLC analysis (method D), 3.05 min, >98% purity. HRMS (ESI+) *m/z* calcd for $C_{26}H_{38}N_7O_2^+$ $[M + H]^+$ 480.3081, found 480.3089. ^1H NMR (300 MHz, DMSO-d_6) δ 9.01 (s, 1H), 7.91 (s, 1H), 7.59 (d, J = 8.6 Hz, 2H), 6.85 (d, J = 9.1 Hz, 2H), 4.30 (d, J = 6.4 Hz, 2H), 3.73 – 3.51 (m, 2H), 3.09 – 2.97 (m, 4H), 2.73 – 2.62 (m, 1H), 2.61 – 2.53 (m, 4H), 2.37 – 2.18 (m, 1H), 1.68 (s, 1H), 1.63 (s, 1H), 1.27 – 1.17 (m, 1H), 1.16 (s, 3H), 1.15 (s, 3H), 1.13 – 1.07 (m, 1H), 1.00 (d, J = 6.5 Hz, 6H). *Purine N-H unresolved.* ^{13}C NMR (126 MHz, MeOD-d_4) δ 156.8, 146.3, 138.0*, 134.0, 120.4, 116.8, 71.7, 70.9, 60.7, 54.7, 49.9, 48.5, 39.4, 31.0, 30.5, 29.0, 20.8, 17.3. *3 carbon signals not visible due to prototautomerism and peak broadening.*[3] **from HSQC, very broad 100 Hz.*

N-(4-(6-Methyl-3,6-diazabicyclo[3.1.1]heptan-3-yl)phenyl)-6-(2,2,2-trifluoroethoxy)-9H-purin-2-amine (21)

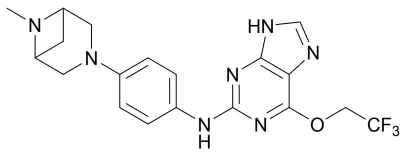

2-Chloro-6-(2,2,2-trifluoroethoxy)-9*H*-purine **(39)** (50.0 mg, 0.100 mmol), 4-(6-methyl-3,6-diazabicyclo[3.1.1]heptan-3-yl)aniline (24.2 mg, 0.120 mmol), *R*-BINAP (9.2 mg, 0.015 mmol) and sodium *tert*-butoxide (19.0 mg, 0.198 mmol) were placed in a MW vial, taken up in 1,4-dioxane (1.0 mL) and degassed with N_2 for 5 min. Tris(dibenzylideneacetone)dipalladium(0) chloroform adduct (10.3 mg, 0.010 mmol) was added, the vial was sealed and further degassed with N_2 for 5 min. The reaction mixture was heated at 120 °C for 1 h under µW irradiation before the crude material was placed through an SCX column, washed with DCM and methanol before eluting with methanolic ammonia. The basic fraction was concentrated under reduced pressure and purified by preparatory HPLC (0.1% NH_3 in MeCN/Water; 5-95%) to yield *N*-[4-(6-methyl-3,6-diazabicyclo[3.1.1]heptan-3-yl)phenyl]-6-(2,2,2-trifluoroethoxy)-9*H*-purin-2-amine **(21)** (5.0 mg, 0.012 mmol, 12% yield) as a white solid. MS (ESI+) *m/z* calcd for $C_{19}H_{21}F_3N_7O^+$ $[M + H]^+$ 420.2, found 420.0. UPLC analysis (method D), 3.28 min, >98% purity. ^{1}H NMR (300 MHz, $CDCl_3$) δ 7.47 (d, *J* = 8.9 Hz, 2H), 7.33 (s, 1H), 6.79 – 6.73 (m, 3H), 4.95 (q, *J* = 8.4 Hz, 2H), 3.90 (d, *J* = 5.9 Hz, 2H), 3.62 (d, *J* = 11.0 Hz, 2H), 3.42 (d, *J* = 11.3 Hz, 2H), 2.84 – 2.67 (m, 1H), 2.27 (s, 3H), 1.73 (d, *J* = 8.8 Hz, 1H). *Purine N-H unresolved.* ^{19}F NMR (282 MHz, $CDCl_3$) δ –73.29 (s).

N-(4-(Piperazin-1-yl)phenyl)-6-(2,2,2-trifluoroethoxy)-9H-purin-2-amine (22)

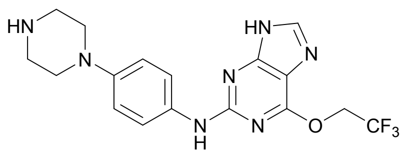

2-Chloro-6-(2,2,2-trifluoroethoxy)-9*H*-purine **(39)** (50.0 mg, 0.100 mmol), 4-(4-aminophenyl) piperazine-1-carboxylic acid tert-butyl ester (41.2 mg, 0.150 mmol) and *p*-toluenesulfonic acid monohydrate (37.7 mg, 0.200 mmol) were taken up in sec-butanol (1.0 mL) and sealed in a microwave vial. The reaction was heated at 120 °C for 1 h under µW irradiation before the crude material was purified by preparatory HPLC (0.1% NH_3 in MeCN/Water; 5-95%) to yield *N*-(4-(piperazin-1-yl)phenyl)-6-(2,2,2-trifluoroethoxy)-9*H*-purin-2-amine **(22)** (5.0 mg, 0.013 mmol, 13% yield) as a white solid. MS (ESI+) *m/z* calcd for $C_{17}H_{19}F_3N_7O^+$ $[M + H]^+$ 394.2, found 394.2. UPLC analysis (method D), 2.69 min, 95% purity. ^{1}H NMR (300 MHz, DMSO-d_6) δ 9.11 (s, 1H), 7.98 (s, 1H), 7.58

(d, J = 9.0 Hz, 2H), 6.86 (d, J = 9.0 Hz, 2H), 5.21 (q, J = 9.1 Hz, 2H), 3.06 – 2.92 (m, 4H), 2.86 – 2.78 (m, 4H). *Purine and piperazine N-Hs unresolved.*

Table 6 Compounds

2,4-Dichloro-7-((2-(trimethylsilyl)ethoxy)methyl)-7*H*-pyrrolo[2,3-*d*]pyrimidine (36)

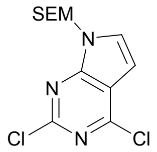

To a cooled (0 °C) and stirred solution of 2,4-dichloro-7*H*-pyrrolo[2,3-*d*]pyrimidine (1.00 g, 5.32 mmol) in THF (10 mL) was added sodium hydride (213 mg, 5.32 mmol) portion wise, followed by 2-(chloromethoxyethyl)trimethyl silane (1.04 mL, 5.85 mmol). The reaction mixture was allowed to warm to RT and stirred for 4 h. After this time the reaction was quenched with water and extracted with Et_2OAc (×2). The combined organic layers were dried (Na_2SO_4), filtered and concentrated under reduced pressure. The crude material was purified by silica gel flash column chromatography (EtOAc/petroleum ether; 0-100%) to yield 2,4-dichloro-7-((2-(trimethylsilyl)ethoxy)methyl)-7*H*-pyrrolo[2,3-*d*]pyrimidine **(36)** (1.53 g, 4.81 mmol, 91% yield) as a colourless oil. MS (ESI+) *m/z* calcd for $C_{12}H_{18}Cl_2N_3OSi^+$ $[M + H]^+$ 318.1, found 318.1. UPLC analysis (method A), 3.69 min, >98% purity. ^1H NMR (300 MHz, $CDCl_3$) δ 7.37 (d, J = 3.7 Hz, 1H), 6.65 (d, J = 3.7 Hz, 1H), 5.60 (s, 2H), 3.63 – 3.44 (m, 2H), 0.98 – 0.81 (m, 2H), –0.05 (s, 9H).

2-Chloro-4-(cyclopropylmethoxy)-7-((2-(trimethylsilyl)ethoxy)methyl)-7*H*-pyrrolo[2,3-*d*]pyrimidine (44)

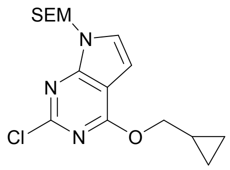

To a cooled (0 °C) and stirred solution of cyclopropylmethanol (34.0 mg, 0.473 mmol) in THF (15 mL) was added sodium hydride (50.0 mg, 1.26 mmol) portion wise and the reaction mixture was stirred at RT for 10 min before a solution of 2,4-dichloro-7-((2-(trimethylsilyl)ethoxy)methyl)-7*H*-pyrrolo[2,3-*d*]pyrimidine **(36)** (150 mg, 0.473 mmol) in THF (3.0 mL) was added drop wise at 0 °C. The resulting reaction mixture was stirred at 60 °C for 16 h before being poured into ice water (50 mL) and extracted with EtOAc (×2). The combined organic layers were dried (Na_2SO_4), filtered and concentrated under

reduced pressure. The crude product was purified by silica gel flash column chromatography (MeOH/DCM; 0-10%) to yield 2-chloro-4-(cyclopropylmethoxy)-7-((2-(trimethylsilyl)ethoxy) methyl)-7*H*-pyrrolo[2,3-*d*]pyrimidine **(44)** (80 mg, 0.23 mmol, 48%). Material carried forward without further purification. MS (ESI+) *m/z* calcd for $C_{16}H_{25}ClN_3O_2Si^+$ [M + H]$^+$, 354.1 found 354.5. UPLC analysis (method E), 3.14 min, 86% purity.

4-(Cyclopropylmethoxy)-*N*-(4-(4-isopropylpiperazin-1-yl)phenyl)-7-((2-(trimethylsilyl)ethoxy) methyl)-7*H*-pyrrolo[2,3-*d*]pyrimidin-2-amine (45)

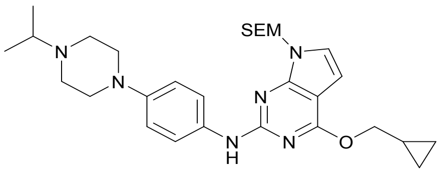

4-(4-Isopropylpiperazin-1-yl)aniline (59 mg, 0.27 mmol) and 2-chloro-4-(cyclopropylmethoxy)-7-((2-(trimethylsilyl)ethoxy)methyl)-7*H*-pyrrolo[2,3-*d*]pyrimidine **(44)** (120 mg, 0.330 mmol) were reacted together according to general procedure B. The crude product was purified by silica gel flash column chromatography (MeOH/DCM; 0-10%) to yield 4-(cyclopropylmethoxy)-*N*-(4-(4-isopropyl-piperazin-1-yl)phenyl)-7-((2-(trimethylsilyl)ethoxy)methyl)-7*H*-pyrrolo[2,3-*d*]pyrimidin-2-amine **(45)** (50 mg, 0.23 mmol, 59%) as yellow solid. MS (ESI+) *m/z* calcd for $C_{29}H_{45}N_6O_2Si^+$ [M + H]$^+$, 537.3 found 537.3. UPLC analysis (method A), 4.50 min.

4-(Cyclopropylmethoxy)-*N*-(4-(4-isopropylpiperazin-1-yl)phenyl)-7*H*-pyrrolo[2,3-*d*]pyrimidin-2-amine (23)

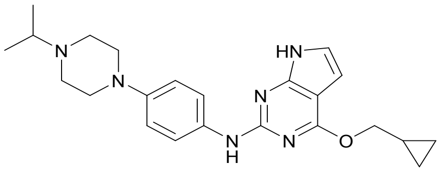

4-(Cyclopropylmethoxy)-*N*-(4-(4-isopropylpiperazin-1-yl)phenyl)-7-((2-(trimethylsilyl)ethoxy) methyl)-7*H*-pyrrolo[2,3-*d*]pyrimidin-2-amine **(45)** (65.0 mg, 0.121 mmol) was reacted and purified according to general procedure C to yield 4-(cyclopropylmethoxy)-*N*-(4-(4-isopropylpiperazin-1-yl)phenyl)-7*H*-pyrrolo[2,3-*d*]pyrimidin-2-amine **(23)** (20 mg, 0.049 mmol, 41%) as an off white solid. MS (ESI+) *m/z* calcd for $C_{23}H_{31}N_6O^+$ [M + H]$^+$ 407.3, found 407.3. UPLC analysis (method D), 4.22 min. HPLC analysis (method F), 4.91 min >98% purity. HRMS (ESI+) *m/z* calcd for $C_{23}H_{31}N_6O^+$ [M + H]$^+$ 407. 2554 Found 407.2571. ^{1}H NMR (500 MHz, DMSO-d$_6$) δ 11.31 (s, 1H), 8.76 (s, 1H),

7.63 (d, J = 9.1 Hz, 2H), 6.92 (dd, J = 3.4, 2.2 Hz, 1H), 6.84 (d, J = 9.1 Hz, 2H), 6.27 (dd, J = 3.4, 1.9 Hz, 1H), 4.28 (d, J = 7.2 Hz, 2H), 3.12 – 2.94 (m, 4H), 2.73 – 2.60 (m, 1H), 2.60 – 2.53 (m, 4H), 1.39 – 1.27 (m, 1H), 1.01 (d, J = 6.5 Hz, 6H), 0.62 – 0.54 (m, 2H), 0.40 – 0.33 (m, 2H). ^{13}C NMR (126 MHz, DMSO-d_6) δ 162.7, 156.0, 154.6, 146.1, 134.3, 120.6, 120.0, 116.4, 98.7, 98.4, 70.4, 54.1, 50.1, 48.6, 18.7, 10.6, 3.7.

2-Chloro-4-(2,2,2-trifluoroethoxy)-7-((2-(trimethylsilyl)ethoxy)methyl)-7*H*-pyrrolo[2,3-*d*]pyrimidine (46)

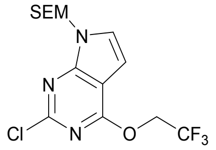

To a cooled (0 °C) and stirred solution of 2,2,2-trifluoroethanol (60.0 mg, 0.630 mmol) in THF (20 mL) was added sodium hydride (50.0 mg, 1.26 mmol) portion wise. Reaction mixture was stirred at RT for 10 min before a solution of 2,4-dichloro-7-((2-(trimethylsilyl)ethoxy)methyl)-7*H*-pyrrolo[2,3-*d*]pyrimidine **(36)** (200 mg, 0.630 mmol) in THF (5 mL) was added dropwise. The resulting reaction mixture was stirred at 60 °C for 16 h before being poured into ice water (50 mL) and extracted with EtOAc (×2). The combined organic layers were dried (Na_2SO_4), filtered and concentrated under reduced pressure. The crude product was purified by silica gel flash column chromatography (MeOH/DCM; 0-10%) to yield 2-chloro-4-(2,2,2-trifluoroethoxy)-7-((2-(trimethylsilyl)ethoxy)methyl)-7*H*-pyrrolo[2,3-*d*]pyrimidine **(46)** (160 mg, 0.420 mmol, 67%) as white solid. MS (ESI+) *m/z* calcd for $C_{14}H_{20}ClF_3N_3O_2Si^+$ $[M + H]^+$ 382.1, found 382.6. UPLC analysis (method E), 3.06 min.

***N*-(4-(4-Isopropylpiperazin-1-yl)phenyl)-4-(2,2,2-trifluoroethoxy)-7-((2-(trimethylsilyl)ethoxy)methyl)-7*H*-pyrrolo[2,3-*d*]pyrimidin-2-amine (47)**

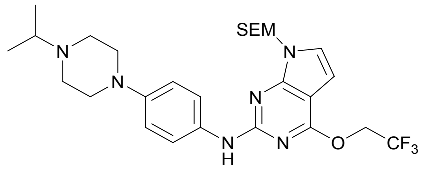

4-(4-Isopropylpiperazin-1-yl)aniline (67 mg, 0.31 mmol) and 2-chloro-4-(cyclopropylmethoxy)-7-((2-(trimethylsilyl)ethoxy)methyl)-7*H*-pyrrolo[2,3-*d*]pyrimidine (**46)** (150 mg, 0.390 mmol) were reacted together according to general procedure B. The crude product was purified by silica gel flash column chromatography (MeOH/DCM; 0-10%) to yield *N*-(4-(4-isopropylpiperazin-1-yl)phenyl)-4-(2,2,2-trifluoroethoxy)-7-((2-(trimethylsilyl)ethoxy)methyl)-7*H*-pyrrolo[2,3-*d*]pyrimidin-2-amine **(47)** (70

mg, 0.12 mmol, 39%) as a yellow solid. Material carried forward without further purification. MS (ESI+) *m/z* calcd for $C_{27}H_{40}F_3N_6O_2Si^+$ [M + H]+, 565.3 found 283.5 [M/2 + H]+. UPLC analysis (method E), 2.28 min. 80% purity.

N-(4-(4-Isopropylpiperazin-1-yl)phenyl)-4-(2,2,2-trifluoroethoxy)-7H-pyrrolo[2,3-d]pyrimidin-2-amine (24)

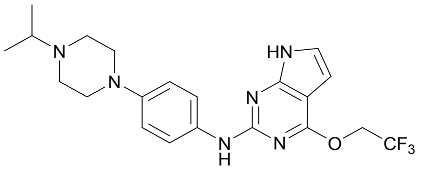

N-(4-(4-Isopropylpiperazin-1-yl)phenyl)-4-(2,2,2-trifluoroethoxy)-7-((2-(trimethylsilyl)ethoxy) methyl)-7*H*-pyrrolo[2,3-*d*]pyrimidin-2-amine **(47)** (60 mg, 0.10 mmol) was reacted and purified according to general procedure C to yield *N*-(4-(4-isopropylpiperazin-1-yl)phenyl)-4-(2,2,2-trifluoroethoxy)-7*H*-pyrrolo[2,3-*d*]pyrimidin-2-amine **(24)** (20 mg, 0.046 mmol, 46%) as an off white solid. MS (ESI+) *m/z* calcd for $C_{21}H_{26}F_3N_6O^+$ [M + H]+, 435.2 found 218.5 [(M/2) + H]+. UPLC analysis (method E), 1.56 min. HPLC analysis (method F), 5.13 min, >96% purity. ^{1}H NMR (400 MHz, DMSO-d_6) δ 11.53 (s, 1H), 8.99 (s, 1H), 7.61 (d, J = 8.8 Hz, 2H), 7.02 (s, 1H), 6.87 (d, J = 8.8 Hz, 2H), 6.31 (s, 1H), 5.21 – 5.14 (m, 2H), 3.10 – 3.04 (m, 4H), 2.57 – 2.50 (m, 5H), 1.00 (d, J = 6.8 Hz, 6H).

2-Chloro-4-((2,2-difluorocyclopropyl)methoxy)-7-((2-(trimethylsilyl)ethoxy)methyl)-7H-pyrrolo[2,3-d]pyrimidine (48)

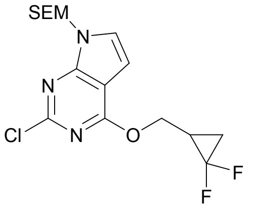

To a cooled (0 °C) and stirred suspension of sodium hydride (41.5 mg, 1.04 mmol) in THF (2.0 mL) was added (2,2-difluorocyclopropyl)methanol (56.0 mg, 0.520 mmol). The mixture was stirred for 5 min before 2,4-dichloro-7-((2-(trimethylsilyl)ethoxy)methyl)-7*H*-pyrrolo[2,3-*d*]pyrimidine **(36)** (150 mg, 0.470 mmol) in THF (2.0 mL) was added and further stirred for 1 h. The reaction mixture was quenched with the addition of water and extracted with EtOAc (×2). The combined organic layers were washed with brine, dried (Na_2SO_4), filtered and concentrated under reduced pressure to yield 2-chloro-4-((2,2-difluorocyclopropyl)methoxy)-7-((2-(trimethylsilyl)ethoxy)methyl)-7*H*-pyrrolo[2,3-*d*] pyrimidine **(48)** (160 mg, 0.410 mmol, 87% yield) as a colourless oil. MS (ESI+) *m/z* calcd for

$C_{16}H_{23}ClF_2N_3O_2Si^+$ [M + H]$^+$ 390.1, found 390.2. UPLC analysis (method A), 3.78 min, >98% purity. ^{1}H NMR (300 MHz, $CDCl_3$) δ 7.17 (d, *J* = 3.6 Hz, 1H), 6.60 (d, *J* = 3.6 Hz, 1H), 5.57 (s, 2H), 4.74 – 4.60 (m, 1H), 4.58 – 4.46 (m, 1H), 3.58 – 3.47 (m, 2H), 2.33 – 2.10 (m, 1H), 1.68 – 1.58 (m, 1H), 1.43 – 1.27 (m, 1H), 0.98 – 0.86 (m, 2H), –0.06 (s, 9H).

4-((2,2-Difluorocyclopropyl)methoxy)-*N*-(4-(4-isopropylpiperazin-1-yl)phenyl)-7*H*-pyrrolo[2,3-*d*]pyrimidin-2-amine (25)

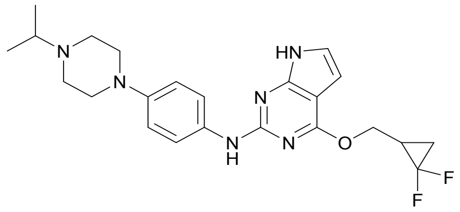

2-Chloro-4-((2,2-difluorocyclopropyl)methoxy)-7-((2-(trimethylsilyl)ethoxy)methyl)-7*H*-pyrrolo[2,3-*d*]pyrimidine **(48)** (80.0 mg, 0.205 mmol), 4-(4-isopropylpiperazin-1-yl)aniline (54.0 mg, 0.246 mmol), sodium tert-butoxide (40.0 mg, 0.416 mmol), tris(dibenzylideneacetone)dipalladium(0) chloroform adduct (10.5 mg, 0.010 mmol) and *R*-BINAP (13 mg, 0.021 mmol) were sealed in a microwave vial. The mixture was taken up in 1,4-dioxane (2.0 mL) and degassed with N_2 for 5 min before heating under µW irradiation at 125 °C for 1 h. The reaction was quenched with sat. aq $NaHCO_3$ and extracted with EtOAc (×3). The combined organic layers were washed with brine, dried (Na_2SO_4), filtered and concentrated under reduced pressure. The crude product was purified by silica gel flash column chromatography (EtOAc/petroleum ether; 1-100%). The resultant orange oil was then reacted according to general procedure C and the crude material taken up in methanol, placed onto an SCX-II cartridge, washed with methanol and eluted with methanolic ammonia. The basic fraction was concentrated under reduced pressure and purified by silica gel flash column chromatography (20% MeOH in DCM/DCM; 0-100%) to yield 4-((2,2-Difluorocyclopropyl)methoxy)-*N*-(4-(4-isopropylpiperazin-1-yl)phenyl)-7*H*-pyrrolo[2,3-*d*]pyrimidin-2-amine **(25)** (33 mg, 0.075 mmol, 37% yield) as an off white solid. MS (ESI+) *m/z* calcd for $C_{23}H_{29}F_2N_6O^+$ [M + H]$^+$ 443.2, found 443.3. UPLC analysis (method D), 3.94 min, >98% purity. ^{1}H NMR (300 MHz, DMSO-d$_6$) δ 11.38 (s, 1H), 8.87 (s, 1H), 7.64 (d, *J* = 8.6 Hz, 2H), 6.95 (dd, *J* = 3.5, 2.2 Hz, 1H), 6.87 (d, *J* = 8.6 Hz, 2H), 6.28 (dd, *J* = 3.5, 1.9 Hz, 1H), 4.68 – 4.55 (m, 1H), 4.45 – 4.35 (m, 1H), 3.03 (br s, 4H), 2.59 (br s, 4H), 2.46 – 2.27 (m, 1H), 1.85 – 1.67 (m, 1H), 1.66 – 1.46 (m, 1H), 1.39 – 1.23 (m, 1H), 1.03 (bs, 6H). ^{19}F NMR (282 MHz, DMSO-d$_6$) δ –127.39 (d, *J* = 156.3 Hz), –141.52 (d, *J* = 156.0 Hz).

2-Chloro-4-((1-methyl-1*H*-indol-4-yl)oxy)-7-((2-(trimethylsilyl)ethoxy)methyl)-7*H*-pyrrolo[2,3-*d*]pyrimidine (49)

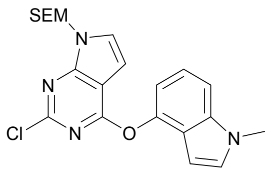

To a stirred solution of 1-methyl-1*H*-indol-4-ol (50.0 mg, 0.340 mmol) in DMF (2.0 mL) was added potassium carbonate (103 mg, 0.747 mmol), followed by 2,4-dichloro-7-((2-(trimethylsilyl)ethoxy)methyl)-7*H*-pyrrolo[2,3-*d*]pyrimidine **(36)** (108 mg, 0.340 mmol) and the reaction mixture heated at 60 °C for 16 h. The reaction mixture was cooled, quenched with sat. aq $NaHCO_3$ and extracted with EtOAc (×2). The combined organic layers were washed with brine, dried (Na_2SO_4), filtered and concentrated under reduced pressure. The crude material was purified by silica gel flash column chromatography (EtOAc/petroleum ether; 0-40%) to yield 2-chloro-4-((1-methyl-1*H*-indol-4-yl)oxy)-7-((2-(trimethylsilyl)ethoxy)methyl)-7*H*-pyrrolo[2,3-*d*]pyrimidine **(49)** (86 mg, 0.20 mmol, 59%) as a colourless oil. MS (ESI+) *m/z* calcd for $C_{21}H_{26}ClN_4O_2Si^+$ $[M + H]^+$ 429.1, found 429.4. UPLC analysis (method B), 3.82 min, 97% purity. ^1H NMR (300 MHz, $CDCl_3$) δ 7.31 – 7.27 (m, 2H), 7.10 (d, *J* = 3.7 Hz, 1H), 7.06 – 7.01 (m, 2H), 6.33 (dd, *J* = 3.2, 0.6 Hz, 1H), 6.01 (d, *J* = 3.7 Hz, 1H), 5.59 (s, 2H), 3.86 (s, 3H), 3.63 – 3.49 (m, 2H), 1.00 – 0.86 (m, 2H), –0.02 (s, 9H).

4-((1-Methyl-1*H*-indol-4-yl)oxy)-*N*-(4-(4-methylpiperazin-1-yl)phenyl)-7-((2-(trimethylsilyl) ethoxy)methyl)-7*H*-pyrrolo[2,3-*d*]pyrimidin-2-amine (50)

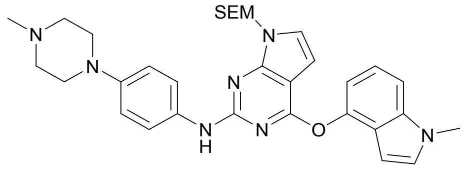

2-Chloro-4-((1-methyl-1*H*-indol-4-yl)oxy)-7-((2-(trimethylsilyl)ethoxy)methyl)-7*H*-pyrrolo[2,3-*d*]pyrimidine **(49)** (130 mg, 0.303 mmol), 4-(4-methylpiperazino)aniline (86.9 mg, 0.455 mmol), sodium tert-butoxide (58.3 mg, 0.606 mmol) and *R*-BINAP (18.9 mg, 0.030 mmol) were placed in a MW vial and taken up in 1,4-dioxane (3.0 mL). The resulting mixture was degassed with N_2 for 10 min before adding tris(dibenzylideneacetone)dipalladium (0) chloroform adduct (15.7 mg, 0.020 mmol), the vial sealed, degassed again with N_2 for 5 min and the reaction mixture heated at 125 °C for 1.25 h under µW irradiation. After this time sat. aq $NaHCO_3$ was added to the reaction mixture and extracted with EtOAc (×2) and the combined organic layers washed with brine, dried (Na_2SO_4), filtered and concentrated under reduced pressure. The crude material was purified by silica gel flash column chromatography (MeOH (0.1% Et_3N)/DCM; 0-5%) to yield 4-((1-methyl-1*H*-indol-4-yl)oxy)-*N*-(4-(4-methylpiperazin-1-yl)phenyl)-7-((2-(trimethylsilyl)ethoxy)methyl)-7*H*-pyrrolo[2,3-*d*]pyrimidin-2-amine **(50)** (115 mg, 0.197 mmol, 65% yield) as a brown oil. MS (ESI+) *m/z* calcd for $C_{32}H_{42}N_7O_2Si^+$

[M + H]$^+$ 584.3, found 584.5. UPLC analysis (method B), 3.24 min, >95% purity. ^{1}H NMR (300 MHz, $CDCl_3$) δ 7.44 (d, *J* = 9.0 Hz, 2H), 7.31 – 7.29 (m, 2H), 7.09 – 6.98 (m, 2H), 6.91 (d, *J* = 6.3 Hz, 1H), 6.82 (d, *J* = 9.0 Hz, 2H), 6.76 (s, 1H), 6.37 (d, *J* = 3.1 Hz, 1H), 6.17 (d, *J* = 3.6 Hz, 1H), 5.54 (s, 2H), 3.86 (s, 3H), 3.67 – 3.54 (m, 2H), 3.21 – 3.07 (m, 4H), 2.71 – 2.54 (m, 4H), 2.39 (s, 3H), 1.03 – 0.87 (m, 2H), –0.05 (s, 9H).

4-((1-Methyl-1*H*-indol-4-yl)oxy)-*N*-(4-(4-methylpiperazin-1-yl)phenyl)-7*H*-pyrrolo[2,3-*d*] pyrimidin-2-amine (26)

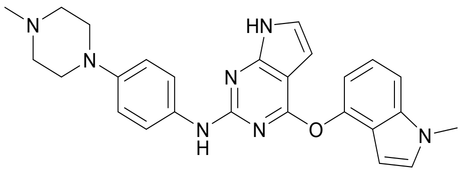

4-((1-Methyl-1*H*-indol-4-yl)oxy)-*N*-(4-(4-methylpiperazin-1-yl)phenyl)-7-((2-(trimethylsilyl)ethoxy) methyl)-7*H*-pyrrolo[2,3-*d*]pyrimidin-2-amine **(50)** was taken up in 4M HCl in 1,4-dioxane (2.0 mL, 0.086 mmol) and water (0.5 mL) and the reaction mixture was stirred for 3.5 h at RT. After this time sat. aq $NaHCO_3$ was added and the resulting solution extracted with EtOAc (×3). The combined organic layers were washed with brine and concentrated under reduced pressure. The resultant brown residue was taken up in 7M ammonia in MeOH (2.0 mL) and stirred for 16 h. The reaction mixture was concentrated under reduced pressure and purified by silica gel flash column chromatography (MeOH (0.1% Et_3N)/DCM; 0-10%) to yield 4-((1-Methyl-1*H*-indol-4-yl)oxy)-*N*-(4-(4-methylpiperazin-1-yl)phenyl)-7*H*-pyrrolo[2,3-*d*]pyrimidin-2-amine **(26)** (24 mg, 0.052 mmol, 62% yield) as a white solid. MS (ESI+) *m/z* calcd for $C_{26}H_{28}N_7O^+$ [M + H]$^+$ 454.2, found 454.4. UPLC analysis (method D), 4.09 min, 97% purity. ^{1}H NMR (300 MHz, $CDCl_3$) δ 8.83 (s, 1H), 7.37 – 7.30 (m, 2H), 7.28 – 7.24 (m, 2H), 7.04 – 7.00 (m, 1H), 6.99 (d, *J* = 3.3 Hz, 1H), 6.84 – 6.76 (m, 2H), 6.68 (s, 1H), 6.66 (dd, *J* = 3.6, 2.1 Hz, 1H), 6.36 (dd, *J* = 3.2, 0.6 Hz, 1H), 6.09 (dd, *J* = 3.6, 2.1 Hz, 1H), 3.83 (s, 3H), 3.17 – 3.08 (m, 4H), 2.66 – 2.57 (m, 4H), 2.37 (s, 3H).

2-Chloro-4-((1-methyl-1*H*-1,2,4-triazol-5-yl)methoxy)-7-((2-(trimethylsilyl)ethoxy)methyl)-7*H*-pyrrolo[2,3-*d*]pyrimidine (51)

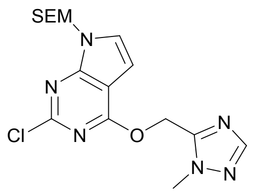

To a cooled (0 °C) and stirred solution of (1-methyl-1*H*-1,2,4-triazol-5-yl)methanol (179 mg, 1.58 mmol) in THF (50 mL) was added sodium hydride (125 mg, 3.15 mmol) portion wise. The reaction mixture was stirred at RT for 10 min before a solution of 2,4-dichloro-7-((2-(trimethylsilyl)ethoxy) methyl)-7*H*-pyrrolo[2,3-*d*]pyrimidine **(36)** (500 mg, 1.58 mmol) in THF (5 mL) was added dropwise. The resulting reaction mixture was stirred at 60 °C for 16 h before being poured into ice water (250 mL) and extracted with EtOAc (×2). The combined organic layers were dried (Na_2SO_4), filtered and concentrated under reduced pressure. The crude product was purified by silica gel flash column chromatography (MeOH/DCM; 0-10%) to yield 2-chloro-4-((1-methyl-1*H*-1,2,4-triazol-5-yl) methoxy)-7-((2-(trimethylsilyl)ethoxy)methyl)-7*H*-pyrrolo[2,3-*d*]pyrimidine **(51)** (300 mg, 0.759 mmol, 48% yield) as a white solid. MS (ESI+) *m/z* calcd for $C_{16}H_{24}ClN_6O_2Si^+$ $[M + H]^+$, 395.1 found 395.1. UPLC analysis (method E), 3.33 min. ^1H NMR (300 MHz, $CDCl_3$) δ 8.06 (s, 1H), 7.13 (d, *J* = 3.6 Hz, 1H), 6.59 (d, *J* = 3.6 Hz, 1H), 5.64 (s, 2H), 5.56 (s, 2H), 3.95 (s, 3H), 3.58 – 3.43 (m, 2H), 0.99 – 0.81 (m, 2H), –0.06 (s, 9H).

4-((1-Methyl-1*H*-1,2,4-triazol-5-yl)methoxy)-*N*-(4-(6-methyl-3,6-diazabicyclo[3.1.1]heptan-3-yl)phenyl)-7-((2-(trimethylsilyl)ethoxy)methyl)-7*H*-pyrrolo[2,3-*d*]pyrimidin-2-amine (52)

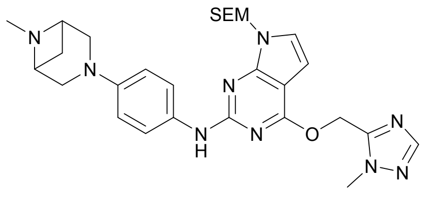

4-(6-Methyl-3,6-diazabicyclo[3.1.1]heptan-3-yl)aniline (40.0 mg, 0.190 mmol) and 2-chloro-4-((1-methyl-1*H*-1,2,4-triazol-5-yl)methoxy)-7-((2-(trimethylsilyl)ethoxy)methyl)-7*H*-pyrrolo[2,3-*d*] pyrimidine **(51)** (77.0 mg, 0.190 mmol) were reacted together according to general procedure B. The crude product was purified by silica gel flash column chromatography (MeOH/DCM; 0-10%) to yield 4-((1-methyl-1*H*-1,2,4-triazol-5-yl)methoxy)-*N*-(4-(6-methyl-3,6-diazabicyclo[3.1.1]heptan-3-yl) phenyl)-7-((2-(trimethylsilyl)ethoxy)methyl)-7*H*-pyrrolo[2,3-*d*]pyrimidin-2-amine **(52)** (70.0 mg, 0.120 mmol, 63% yield) as a yellow solid. Material carried forward without further purification. MS (ESI+) *m/z* calcd for $C_{28}H_{40}N_9O_2Si^+$ $[M + H]^+$, 562.3 found 562.7. UPLC analysis (method E), 2.06 min.

4-((1-Methyl-1*H*-1,2,4-triazol-5-yl)methoxy)-*N*-(4-(6-methyl-3,6-diazabicyclo[3.1.1]heptan-3-yl)phenyl)-7*H*-pyrrolo[2,3-*d*]pyrimidin-2-amine (27)

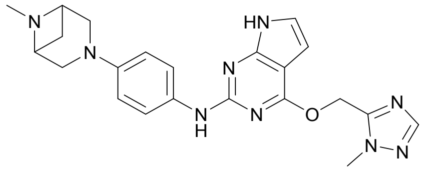

To a solution of 4-((1-methyl-1*H*-1,2,4-triazol-5-yl)methoxy)-*N*-(4-(6-methyl-3,6-diazabicyclo[3.1.1] heptan-3-yl)phenyl)-7-((2-(trimethylsilyl)ethoxy)methyl)-7*H*-pyrrolo[2,3-*d*]pyrimidin-2-amine **(52)** (70.0 mg, 0.120 mmol) in DCM (1.0 mL) was added 4M HCl in 1,4-dioxane (1.5 mL) and the reaction mixture stirred at RT for 2 h. The reaction mixture was concentrated under reduced pressure before being taken up 7M ammonia in methanol (2.0 mL). The reaction mixture was then further stirred at RT for 16 h before being concentrated under reduced pressure. The crude material was purified by reverse phase column chromatography (0.1% NH_3 in MeCN/Water; 40-60%) to yield 4-((1-methyl-1*H*-1,2,4-triazol-5-yl)methoxy)-*N*-(4-(6-methyl-3,6-diazabicyclo[3.1.1]heptan-3-yl)phenyl)-7*H*-pyrrolo[2,3-*d*]pyrimidin-2-amine **(27)** (10 mg, 0.023 mmol, 19%) as a brown solid. MS (ESI+) *m/z* calcd for $C_{22}H_{26}N_9O^+$ $[M + H]^+$, 432.2 found 432.3. UPLC analysis (method E), 1.15 min. HPLC analysis (method E), 3.93 min, >98% purity. ^1H NMR (400 MHz, DMSO-d_6) δ 11.33 (s, 1H), 8.75 (s, 1H), 8.49 (s, 1H), 7.63 (d, J = 8.4 Hz, 2H), 6.92 (s, 1H), 6.66 (d, J = 8.8 Hz, 2H), 6.22 (s, 1H), 5.49 (s, 2H), 3.88 (s, 3H), 3.59 – 3.65 (m, 2H), 3.48 – 3.43 (m, 2H), 3.35 – 3.28 (m, 2H), 3.21 – 3.17 (m, 1H), 2.04 (s, 3H), 1.55 (d, J = 7.6 Hz, 1H).

***N*-(4-(6-Methyl-3,6-diazabicyclo[3.1.1]heptan-3-yl)phenyl)-4-(2,2,2-trifluoroethoxy)-7-((2-(trimethylsilyl)ethoxy)methyl)-7*H*-pyrrolo[2,3-*d*]pyrimidin-2-amine (53)**

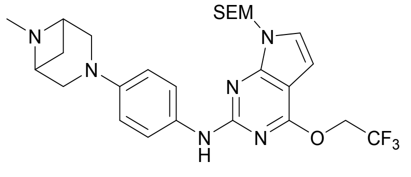

4-(6-Methyl-3,6-diazabicyclo[3.1.1]heptan-3-yl)aniline (70.0 mg, 0.350 mmol) and 2-chloro-4-(2,2,2-trifluoroethoxy)-7-((2-(trimethylsilyl)ethoxy)methyl)-7*H*-pyrrolo[2,3-*d*]pyrimidine **(46)** (170 mg, 0.440 mmol) were reacted together according to general procedure B. The crude product was purified by silica gel flash column chromatography (MeOH/DCM; 0-10%) to yield *N*-(4-(6-methyl-3,6-diazabicyclo[3.1.1]heptan-3-yl)phenyl)-4-(2,2,2-trifluoroethoxy)-7-((2-(trimethylsilyl)ethoxy) methyl)-7*H*-pyrrolo[2,3-*d*]pyrimidin-2-amine **(53)** (90 mg, 0.16 mmol, 48%) as yellow solid. MS (ESI+) *m/z* calcd for $C_{26}H_{36}F_3N_6O_2Si^+$ $[M + H]^+$, 549.3 found 275.7 $[M/2 + H]^+$. UPLC analysis (method E), 2.34 min, 93% purity.

***N*-(4-(6-Methyl-3,6-diazabicyclo[3.1.1]heptan-3-yl)phenyl)-4-(2,2,2-trifluoroethoxy)-7*H*-pyrrolo[2,3-*d*]pyrimidin-2-amine (28)**

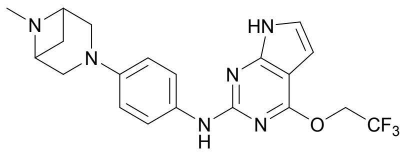

To a stirred solution of *N*-(4-(6-methyl-3,6-diazabicyclo[3.1.1]heptan-3-yl)phenyl)-4-(2,2,2-trifluoroethoxy)-7-((2-(trimethylsilyl)ethoxy)methyl)-7*H*-pyrrolo[2,3-*d*]pyrimidin-2-amine **(46)** (50 mg, 0.090 mmol) in DCM (1.0 mL) was added 4M HCl in 1,4-dioxane (1.5 mL) and the reaction mixture stirred at RT for 2 h. The reaction mixture was concentrated under reduced pressure before being taken up in 7M ammonia in methanol (1.0 mL) and further stirred at RT for 16 h. After this time the reaction mixture was concentrated under reduced pressure and the crude product was purified by reverse phase column chromatography (0.1% NH_3 in MeCN/Water; 40-60%) to yield *N*-(4-(6-methyl-3,6-diazabicyclo[3.1.1]heptan-3-yl)phenyl)-4-(2,2,2-trifluoroethoxy)-7*H*-pyrrolo[2,3-*d*]pyrimidin-2-amine **(28)** (10 mg, 0.024 mmol, 27%) as an off white solid. MS (ESI+) *m/z* calcd for $C_{20}H_{22}F_3N_6O_2^+$ $[M + H]^+$, 419.2 found 419.1. UPLC analysis (method E), 1.81 min. HPLC analysis (method F), 5.09 min, >98% purity. ^1H NMR (400 MHz, DMSO-d$_6$) δ 11.47 (s, 1H), 8.86 (s, 1H), 7.60 (d, *J* = 8.6 Hz, 2H), 6.99 (s, 1H), 6.68 (d, *J* = 8.8 Hz, 2H), 6.30 (s, 1H), 5.21 – 5.14 (m, 2H), 3.62 (s, 2H), 3.47 – 3.34 (m, 2H), 3.34 – 3.23 (s, 2H), 2.51 – 2.40 (m, 1H), 2.03 (s, 3H), 1.61 – 1.52 (m, 1H)

***N*-(4-(4-Isopropylpiperazin-1-yl)phenyl)-4-((1-methyl-1*H*-1,2,4-triazol-5-yl)methoxy)-7-((2-(trimethylsilyl)ethoxy)methyl)-7*H*-pyrrolo[2,3-*d*]pyrimidin-2-amine (54)**

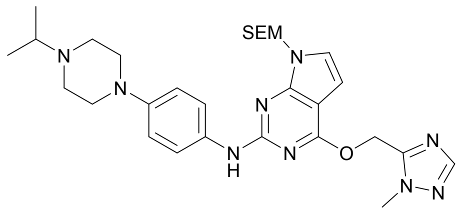

4-(4-Isopropylpiperazin-1-yl)aniline (150 mg, 0.380 mmol) and 2-chloro-4-((1-methyl-1*H*-1,2,4-triazol-5-yl)methoxy)-7-((2-(trimethylsilyl)ethoxy)methyl)-7*H*-pyrrolo[2,3-*d*]pyrimidine **(51)** (60.0 mg, 0.300 mmol) were reacted together according to general procedure B. The crude product was purified by silica gel flash column chromatography (MeOH/DCM; 0-10%) to yield *N*-(4-(4-isopropylpiperazin-1-yl)phenyl)-4-((1-methyl-1*H*-1,2,4-triazol-5-yl)methoxy)-7-((2-(trimethylsilyl) ethoxy)methyl)-7*H*-pyrrolo[2,3-*d*]pyrimidin-2-amine **(54)** (70.0 mg, 0.120 mmol) as a yellow solid. The material was carried forward without further purification. MS (ESI+) *m/z* calcd for $C_{29}H_{44}N_9O_2Si^+$ $[M + H]^+$ 578.3, found 578.4. UPLC analysis (method E), 1.73 min.

N-(4-(4-Isopropylpiperazin-1-yl)phenyl)-4-((1-methyl-1H-1,2,4-triazol-5-yl)methoxy)-7H-pyrrolo[2,3-d]pyrimidin-2-amine Formate (29)

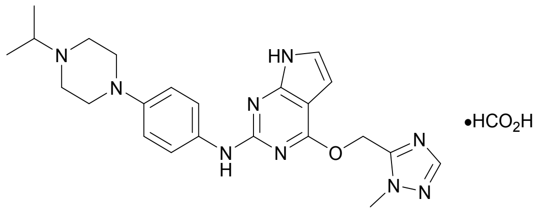

N-(4-(4-Isopropylpiperazin-1-yl)phenyl)-4-((1-methyl-1*H*-1,2,4-triazol-5-yl)methoxy)-7-((2-(trimethylsilyl)ethoxy)methyl)-7*H*-pyrrolo[2,3-*d*]pyrimidin-2-amine **(54)** (60.0 mg, 0.100 mmol) was reacted according to general procedure C and purified by preparatory HPLC (0.1% formic acid in MeCN/Water; 16-98%) to yield *N*-(4-(4-isopropylpiperazin-1-yl)phenyl)-4-((1-methyl-1*H*-1,2,4-triazol-5-yl)methoxy)-7*H*-pyrrolo[2,3-*d*]pyrimidin-2-amine formate **(29)** (10 mg, 0.022 mmol, 22%) as a pale yellow solid.[4] MS (ESI+) *m/z* calcd for $C_{23}H_{30}N_9O^+$ [M + H]$^+$ 448.3, found 448.4. UPLC analysis (method E), 1.22 min. HPLC analysis (method F), 3.81 min, >98% purity. HRMS (ESI+) *m/z* calcd for $C_{23}H_{30}N_9O^+$ [M + H]$^+$ 448.2568, found 448.2571. ^{1}H NMR (500 MHz, DMSO-d_6) δ 11.37 (s, 1H), 8.86 (s, 1H), 8.48 (s, 1H), 8.21 (s, 1H), 7.64 (d, *J* = 9.0 Hz, 2H), 6.94 (dd, *J* = 3.4, 2.3 Hz, 1H), 6.85 (d, *J* = 9.1 Hz, 2H), 6.23 (dd, *J* = 3.4, 1.9 Hz, 1H), 5.50 (s, 2H), 3.87 (s, 3H), 3.09 – 2.99 (m, 4H), 2.72 – 2.63 (m, 1H), 2.64 – 2.55 (m, 4H), 1.01 (d, *J* = 6.5 Hz, 6H). ^{13}C NMR (126 MHz, DMSO-d_6) δ 164.2, 162.0, 159.7, 155.8, 154.9, 146.1, 145.9, 134.1, 120.9, 120.1, 116.4, 98.6, 98.2, 60.4, 54.1, 50.1, 48.6, 36.3, 18.7.

4-(Cyclopropylmethoxy)-N-(4-(6-methyl-3,6-diazabicyclo[3.1.1]heptan-3-yl)phenyl)-7-((2-(trimethylsilyl)ethoxy)methyl)-7H-pyrrolo[2,3-d]pyrimidin-2-amine (55)

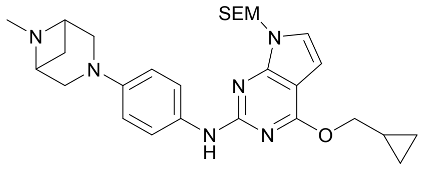

4-(6-Methyl-3,6-diazabicyclo[3.1.1]heptan-3-yl)aniline (100 mg, 0.490 mmol) and 2-chloro-4-(cyclopropylmethoxy)-7-((2-(trimethylsilyl)ethoxy)methyl)-7*H*-pyrrolo[2,3-*d*]pyrimidine **(44)** (140 mg, 0.390 mmol) were reacted together according to general procedure B. The crude product was purified by silica gel flash column chromatography (MeOH/DCM; 0-10%) to yield 4-(cyclopropylmethoxy)-*N*-(4-(6-methyl-3,6-diazabicyclo[3.1.1]heptan-3-yl)phenyl)-7-((2-(trimethyl-silyl)ethoxy)methyl)-7*H*-pyrrolo[2,3-*d*]pyrimidin-2-amine **(55)** (120 mg, 0.230 mmol, 59%) as a yellow solid. Material carried forward without further purification. MS (ESI+) *m/z* calcd for $C_{28}H_{41}N_6O_2Si^+$ [M + H]$^+$, 521.3 found 521.7. UPLC analysis (method E), 3.10 min.

4-(Cyclopropylmethoxy)-*N*-(4-(6-methyl-3,6-diazabicyclo[3.1.1]heptan-3-yl)phenyl)-7*H*-pyrrolo[2,3-*d*]pyrimidin-2-amine (30)

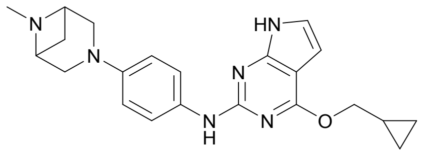

4-(Cyclopropylmethoxy)-*N*-(4-(6-methyl-3,6-diazabicyclo[3.1.1]heptan-3-yl)phenyl)-7-((2-(trimethylsilyl)ethoxy)methyl)-7*H*-pyrrolo[2,3-*d*]pyrimidin-2-amine **(55)** (100 mg, 0.190 mmol) was reacted and purified according to general procedure C to yield 4-(cyclopropylmethoxy)-*N*-(4-(6-methyl-3,6-diazabicyclo[3.1.1]heptan-3-yl)phenyl)-7*H*-pyrrolo[2,3-*d*]pyrimidin-2-amine **(30)** (20 mg, 0.051 mmol, 27%) as an off white solid. MS (ESI+) *m/z* calcd for $C_{22}H_{27}N_6O^+$ [M + H]$^+$, 391.2 found 391.7. UPLC analysis (method E), 1.43 min. HPLC analysis (Method F), 4.42min, >96% purity. ^{1}H NMR (400 MHz, DMSO-d$_6$) δ 11.28 (s, 1H), 8.67 (s, 1H), 7.63 (d, *J* = 8.8 Hz, 2H), 6.90 (s, 1H), 6.66 (d, *J* = 8.4 Hz, 2H), 6.26 (s, 1H), 4.28 (d, *J* = 7.2 Hz, 2H), 3.65 (br s, 2H), 3.49 – 3.30 (m, 4H), (2 protons overlap with solvent peak) 2.10 – 2.02 (s, 3H), 1.64 – 1.58 (m, 1H), 1.37 – 1.34 (m, 2H), 0.58 (d, *J* = 6.8 Hz, 2H), 0.36 (d, *J* = 4.4 Hz, 2H).

***tert*-Butyl 6-(4-((4-((2,2-difluorocyclopropyl)methoxy)-7-((2-(trimethylsilyl)ethoxy)methyl)-7*H*-pyrrolo[2,3-*d*]pyrimidin-2-yl)amino)phenyl)-2,6-diazaspiro[3.3]heptane-2-carboxylate (56)**

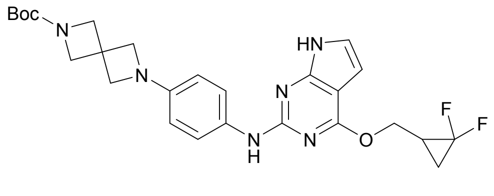

A mixture of palladium (II) acetate (8.5 mg, 0.040 mmol), *tert*-butyl 6-(4-aminophenyl)-2,6-diazaspiro[3.3]heptane-2-carboxylate (118 mg, 0.409 mmol), Xantphos (21.5 mg, 0.040 mmol), tripotassium phosphate (158 mg, 0.740 mmol) and 2-chloro-4-((2,2-difluorocyclopropyl)methoxy)-7-((2-(trimethylsilyl)ethoxy)methyl)-7*H*-pyrrolo[2,3-*d*]pyrimidine **(48)** (145 mg, 0.372 mmol) in 1,4-dioxane (4.0 mL) was sealed in a MW, degassed with nitrogen for 5 min and the reaction mixture heated at 80 °C for 1.5 h. After this time the reaction mixture was cooled and placed on an SCX-II cartridge, washed with DCM and MeOH and eluted with methanolic ammonia. The basic fraction was concentrated under reduced pressure and purified by silica gel flash column chromatography (EtOAc/petroleum ether; 5-100%) to yield *tert*-butyl 6-(4-((4-((2,2-difluorocyclopropyl)methoxy)-7-((2-(trimethylsilyl)ethoxy)methyl)-7*H*-pyrrolo[2,3-*d*]pyrimidin-2-yl)amino)phenyl)-2,6-diazaspiro [3.3]heptane-2-carboxylate **(56)** (103 mg, 0.160 mmol, 43% yield). Material carried forward to the next

step without further purification. MS (ESI+) *m/z* calcd for $C_{32}H_{44}F_2N_6O_4Si^+$ [M + H]$^+$ 643.3, found 643.3. UPLC analysis (method B), 3.96 min, >86% purity.

tert-Butyl 6-(4-((4-((2,2-difluorocyclopropyl)methoxy)-7*H*-pyrrolo[2,3-*d*]pyrimidin-2-yl)amino)phenyl)-2,6-diazaspiro[3.3]heptane-2-carboxylate (57)

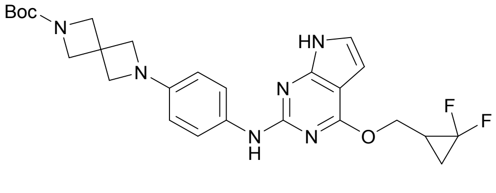

tert-Butyl 6-(4-((4-((2,2-difluorocyclopropyl)methoxy)-7-((2-(trimethylsilyl)ethoxy)methyl)-7*H*-pyrrolo[2,3-*d*]pyrimidin-2-yl)amino)phenyl)-2,6-diazaspiro[3.3]heptane-2-carboxylate **(56)** (100 mg, 0.156 mmol) was reacted and purified according to general procedure C to yield *tert*-butyl 6-(4-((4-((2,2-difluorocyclopropyl)methoxy)-7*H*-pyrrolo[2,3-*d*]pyrimidin-2-yl)amino)phenyl)-2,6-diazaspiro [3.3]heptane-2-carboxylate **(57)** (63.0 mg, 0.123 mmol, 79%) as an off white solid. MS (ESI+) *m/z* calcd for $C_{26}H_{31}F_2N_6O_3^+$ [M + H]$^+$ 513.2, found 513.3. UPLC analysis (method B), 3.34 min, >98% purity. ^{1}H NMR (300 MHz, MeOD-d$_4$) δ 7.58 – 7.46 (m, 2H), 6.87 (d, *J* = 3.6 Hz, 1H), 6.59 – 6.47 (m, 2H), 6.34 (d, *J* = 3.5 Hz, 1H), 4.70 – 4.56 (m, 1H), 4.50 – 4.41 (m, 1H), 4.13 – 4.09 (m, 4H), 3.96 – 3.93 (m, 4H), 2.37 – 2.22 (m, 1H), 1.71 – 1.52 (m, 1H), 1.47 (s, 9H), 1.44 – 1.35 (m, 1H). *Pyrrolo and anilino N-H protons unresolved.*

4-((2,2-Difluorocyclopropyl)methoxy)-*N*-(4-(6-methyl-2,6-diazaspiro[3.3]heptan-2-yl)phenyl)-7*H*-pyrrolo[2,3-*d*]pyrimidin-2-amine (31)

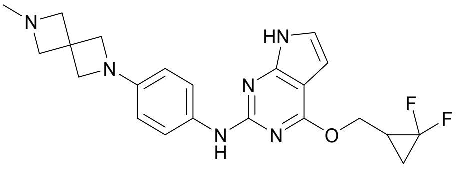

To a solution of *tert*-butyl 6-(4-((4-((2,2-difluorocyclopropyl)methoxy)-7*H*-pyrrolo[2,3-*d*]pyrimidin-2-yl)amino)phenyl)-2,6-diazaspiro[3.3]heptane-2-carboxylate **(57)** (63.0 mg, 0.120 mmol) in DCM (2.0 mL) was added trifluoroacetic acid (0.77 mL, 10.0 mmol) and the reaction mixture stirred at RT for 1 h. After this time the solvent was removed under reduced pressure and co-evaporated with toluene to give a pale-yellow residue. The residue was taken up in DCE (2.0 mL) and MeOH (1.0 mL) and to this was added acetic acid (0.04 mL, 0.740 mmol), formaldehyde (37% aq solution, 0.14 mL, 1.85 mmol) and sodium triacetoxyborohydride (182 mg, 0.861 mmol) and the reaction mixture was stirred at RT for 3 h. After this time the reaction mixture was diluted with sat. aq $NaHCO_3$ and extracted

with DCM (×3). The combined organic layers were washed with brine, dried (Na_2SO_4), filtered and concentrated under reduced pressure. The crude material was purified by preparatory HPLC (0.1% NH_3 in MeCN/Water; 40-80%) to yield 4-((2,2-difluorocyclopropyl)methoxy)-*N*-(4-(6-methyl-2,6-diazaspiro[3.3]heptan-2-yl)phenyl)-7*H*-pyrrolo[2,3-*d*]pyrimidin-2-amine **(31)** (17 mg, 0.040 mmol, 32%) as a white solid. MS (ESI+) *m/z* calcd for $C_{22}H_{25}F_2N_6O^+$ [M + H]$^+$ 427.2, found 427.2. UPLC analysis (method D), 3.76 min, >98% purity. HRMS (ESI+) *m/z* calcd for $C_{22}H_{24}F_2N_6O^+$ [M - *e*]$^+$ 426.1980, found 426.1972. ^{1}H NMR (300 MHz, MeOD-d$_4$) δ 7.57 – 7.45 (m, 2H), 6.87 (d, *J* = 3.6 Hz, 1H), 6.57 – 6.45 (m, 2H), 6.34 (d, *J* = 3.5 Hz, 1H), 4.70 – 4.55 (m, 1H), 4.50 – 4.38 (m, 1H), 3.92 – 3.86 (m, 4H), 3.51 – 3.43 (m, 4H), 2.37 (s, 3H), 2.35 – 2.17 (m, 1H), 1.68 – 1.55 (m, 1H), 1.48 – 1.30 (m, 1H). *Pyrrolo and aniline N-H protons unresolved.* ^{13}C NMR (75 MHz, MeOD-d$_4$) δ 163.9, 157.8, 155.7, 148.7, 134.0, 122.3, 120.9, 115.2 (t, *J* = 281.9 Hz), 113.7, 99.7, 99.6, 66.7, 63.8 (d, *J* = 5.9 Hz), 63.5, 45.5, 35.6, 22.7 (t, *J* = 11.1 Hz), 15.5 (t, *J* = 11.2 Hz). ^{19}F NMR (282 MHz, MeOD-d$_4$) δ –130.39 (d, *J* = 160.2 Hz), – 145.10 (d, *J* = 159.9 Hz).

<u>Table 7 Compounds</u>

6-Chloro-4-(2,2,2-trifluoroethoxy)-1-((2-(trimethylsilyl)ethoxy)methyl)-1*H*-pyrrolo[2,3-*b*]pyridine (58)

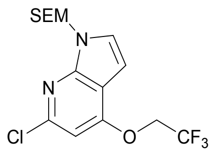

To a cooled (0 °C) and stirred suspension of sodium hydride (37.8 mg, 0.946 mmol) in DMF (1.0 mL) was added 2,2,2-trifluoroethanol (81.0 mg, 0.810 mmol) in THF (1.0 mL) and stirred for 10 min. 2,6-dichloro-9-((2-(trimethylsilyl)ethoxy)methyl)-9*H*-purine (150 mg, 0.473 mmol) in DMF (2.5 mL) was then added and the reaction mixture stirred at RT for 18 h. After this time the reaction was quenched with water and extracted with DCM:IPA (4:1), followed by EtOAc. The combined organic layers were dried (Na_2SO_4), filtered and concentrated under reduced pressure. The crude material was purified by silica gel flash column chromatography (EtOAc/petroleum ether; 0-20%) to yield 6-Chloro-4-(2,2,2-trifluoroethoxy)-1-((2-(trimethylsilyl)ethoxy)methyl)-1*H*-pyrrolo[2,3-*b*]pyridine **(58)** (70.0 mg, 0.184 mmol, 39% yield). MS (ESI+) *m/z* calcd for $C_{15}H_{21}ClF_3N_2O_2Si^+$ [M + H]$^+$ 381.1, found 381.2. UPLC analysis (method A), 3.78 min, 89% purity. ^{1}H NMR (300 MHz, $CDCl_3$) δ 7.23 (d, *J* = 3.6 Hz, 1H), 6.61 (d, *J* = 3.6 Hz, 1H), 6.56 (s, 1H), 5.61 (s, 2H), 4.54 (q, *J* = 7.9 Hz, 2H), 3.60 – 3.46 (m, 2H), 0.96 – 0.85 (m, 2H), –0.06 (s, 9H).

N-(4-(6-Methyl-3,6-diazabicyclo[3.1.1]heptan-3-yl)phenyl)-4-(2,2,2-trifluoroethoxy)-1H-pyrrolo[2,3-b]pyridin-6-amine (32)

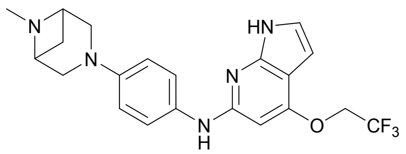

4-(6-Methyl-3,6-diazabicyclo[3.1.1]heptan-3-yl)aniline (50.0 mg, 0.246 mmol) and 6-chloro-4-(2,2,2-trifluoroethoxy)-1-((2-(trimethylsilyl)ethoxy)methyl)-1*H*-pyrrolo[2,3-*b*]pyridine **(58)** (70.0 mg, 0.184 mmol) were reacted according to general procedure B and partially purified by silica gel flash column chromatography (EtOAc/petroleum ether; 5-100%, followed by MeOH/DCM; 0-20%) to give a yellow oil. The material was taken up in 4 M HCl in 1,4-dioxane (2.0 mL) and water (0.5 mL) and stirred at 50 °C for 1.5 h. After this time the reaction mixture was cooled, quenched with sat. aq $NaHCO_3$ and extracted with EtOAc. The organic layer was dried (Na_2SO_4), filtered and concentrated under reduced pressure. The resulting residue was taken up in 7M methanolic ammonia (5.0 mL) and stirred at RT for 16 h followed by heating at 50 °C for 2 h before the reaction mixture was concentrated under reduced pressure and the crude material purified by preparatory HPLC (0.1% NH_3 in MeCN/Water; 30-70%) to yield *N*-(4-(6-methyl-3,6-diazabicyclo[3.1.1]heptan-3-yl)phenyl)-4-(2,2,2-trifluoroethoxy)-1*H*-pyrrolo[2,3-*b*]pyridin-6-amine **(32)** (15 mg, 0.036 mmol, 33% yield) as a white solid. MS (ESI+) *m/z* calcd for $C_{21}H_{23}F_3N_5O^+$ $[M + H]^+$ 418.2, found 418.2. UPLC analysis (method D), 4.42 min, >95% purity. ^1H NMR (300 MHz, DMSO-d_6) δ 11.10 (s, 1H), 8.40 (s, 1H), 7.54 (d, J = 9.0 Hz, 2H), 6.91 (dd, J = 3.4, 2.3 Hz, 1H), 6.66 (d, J = 9.1 Hz, 2H), 6.23 (dd, J = 3.4, 2.0 Hz, 1H), 6.08 (s, 1H), 4.85 (q, J = 8.8 Hz, 2H), 3.56 (d, J = 5.8 Hz, 2H), 3.41 (d, J = 10.9 Hz, 2H), 3.25 (d, J = 10.9 Hz, 2H), 2.46 – 2.36 (m, 1H), 1.98 (s, 3H), 1.54 (d, J = 8.0 Hz, 1H).

2,4-Dichloro-5-((2-(trimethylsilyl)ethoxy)methyl)-5H-pyrrolo[3,2-d]pyrimidine (59)

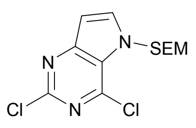

To a cooled (0 °C) and stirred solution of 2,4-dichloro-5*H*-pyrrolo[3,2-*d*]pyrimidine (250 mg, 1.33 mmol) in THF (13.3 mL) was added sodium hydride (106 mg, 2.66 mmol) and the reaction mixture stirred for 5 min until effervescence ceased. 2-(Chloromethoxyethyl)trimethyl silane (244 mg, 1.46 mmol) was then added and the reaction mixture stirred for 1 h. The reaction was quenched with the addition of water, and the aqueous layer extracted with EtOAc (×2). The combined organic layers were washed with brine, dried (Na_2SO_4), filtered and concentrated under reduced pressure to yield 2,4-dichloro-5-((2-(trimethylsilyl)ethoxy)methyl)-5*H*-pyrrolo[3,2-*d*]pyrimidine **(59)** (415 mg, 1.31 mmol,

98% yield) as a pale yellow oil. MS (ESI+) *m/z* calcd for $C_{12}H_{18}Cl_2N_3OSi^+$ $[M + H]^+$ 318.1, found 318.0. UPLC analysis (method D), 3.57 min, 97% purity. ^{1}H NMR (300 MHz, $CDCl_3$) δ 7.64 (d, *J* = 3.3 Hz, 1H), 6.71 (d, *J* = 3.3 Hz, 1H), 5.75 (s, 2H), 3.60 – 3.42 (m, 2H), 0.94 – 0.87 (m, 2H), –0.04 (s, 9H).

2-Chloro-4-((2,2-difluorocyclopropyl)methoxy)-5-((2-(trimethylsilyl)ethoxy)methyl)-5*H*-pyrrolo[3,2-*d*]pyrimidine (60)

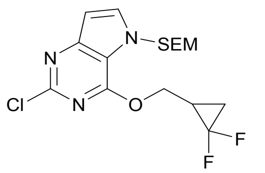

To a cooled (0 °C) and stirred solution of (2,2-difluorocyclopropyl)methanol (53.5 mg, 0.495 mmol) in THF (5.0 mL) was added sodium hydride (38.0 mg, 0.950 mmol) and the reaction mixture stirred for 5 min before 2,4-dichloro-5-((2-(trimethylsilyl)ethoxy)methyl)-5*H*-pyrrolo[3,2-*d*]pyrimidine **(59)** (150 mg, 0.471 mmol) was added and the reaction mixture stirred at RT for 2.5 h. The reaction was quenched with water and the aqueous layer extracted with EtOAc (×2). The combined organic layers were washed with brine, dried (Na_2SO_4), filtered and concentrated under reduced pressure to yield 2-chloro-4-((2,2-difluorocyclopropyl)methoxy)-5-((2-(trimethylsilyl)ethoxy)methyl)-5*H*-pyrrolo[3,2-*d*]pyrimidine **(60)** (170 mg, 0.436 mmol, 93% yield) as a pale yellow oil. Material carried forward without further purification. MS (ESI+) *m/z* calcd for $C_{16}H_{23}ClF_2N_3O_2Si^+$ $[M + H]^+$ 390.1, found 390.1. UPLC analysis (method B), 3.61 min, 79% purity. ^{1}H NMR (300 MHz, $CDCl_3$) δ 7.45 (d, *J* = 3.2 Hz, 1H), 6.61 (d, *J* = 3.2 Hz, 1H), 5.63 (s, 2H), 4.94 – 4.75 (m, 1H), 4.51 – 4.37 (m, 1H), 3.60 – 3.41 (m, 2H), 2.31 – 2.11 (m, 1H), 1.69 – 1.54 (m, 1H), 1.44 – 1.31 (m, 1H), 0.95 – 0.80 (m, 2H), –0.06 (s, 9H). ^{19}F NMR (282 MHz, $CDCl_3$) δ –129.25 (d, *J* = 160.9 Hz), –143.25 (d, *J* = 160.8 Hz).

4-((2,2-Difluorocyclopropyl)methoxy)-*N*-(4-(4-isopropylpiperazin-1-yl)phenyl)-5*H*-pyrrolo[3,2-*d*]pyrimidin-2-amine (33)

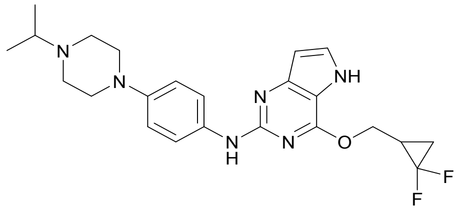

2-Chloro-4-((2,2-difluorocyclopropyl)methoxy)-5-((2-(trimethylsilyl)ethoxy)methyl)-5*H*-pyrrolo[3,2-*d*]pyrimidine **(60)** (85.0 mg, 0.218 mmol) and 4-(4-isopropylpiperazin-1-yl)aniline (57.3 mg, 0.262 mmol) were reacted according to general procedure B. The crude product was then partial

purified by silica gel flash column chromatography (MeOH/DCM; 0-20%). The resultant material was then reacted according to general procedure C and purified by preparatory HPLC (0.1% NH_3 in MeCN/Water; 30-70%) to yield 4-((2,2-difluorocyclopropyl)methoxy)-*N*-(4-(4-isopropylpiperazin-1-yl)phenyl)-5*H*-pyrrolo[3,2-*d*]pyrimidin-2-amine **(33)** (23.6 mg, 0.053 mmol, 24% yield) as an off white solid. MS (ESI+) *m/z* calcd for $C_{23}H_{29}F_2N_6O^+$ [M + H]$^+$ 443.2, found 443.3. UPLC analysis (method D), 2.77 min, >98% purity. ^{1}H NMR (300 MHz, DMSO-d$_6$) δ 11.55 (s, 1H), 8.66 (s, 1H), 7.66 (d, *J* = 8.5 Hz, 2H), 7.56 – 7.38 (m, 1H), 6.85 (d, *J* = 8.6 Hz, 2H), 6.23 (s, 1H), 4.83 – 4.66 (m, 1H), 4.49 – 4.26 (m, 1H), 3.14 – 2.93 (m, 4H), 2.77 – 2.65 (m, 1H), 2.65 – 2.54 (m, 4H), 2.44 – 2.32 (m, 1H), 1.90 – 1.67 (m, 1H), 1.69 – 1.51 (m, 1H), 1.01 (d, *J* = 6.5 Hz, 6H).

8-Bromo-6-chloroimidazo[1,2-*b*]pyridazine (61)

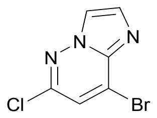

Chloroacetaldehyde (0.24 mL, 1.92 mmol) (50% wt in H2O) was added dropwise to a suspension of 4-bromo-6-chloropyridazin-3-amine (100 mg, 0.480 mmol) in ethanol (4.8 mL) and heated at 85 °C for 2 h. The reaction mixture was poured into ice water, basified with sat. aq $NaHCO_3$ and extracted with EtOAc (×2). The combined organic layers were washed with brine, dried (Na_2SO_4), filtered and concentrated under reduced pressure. The crude material was purified by silica gel flash column chromatography (EtOAc/petroleum ether; 0-40%) to yield 8-bromo-6-chloroimidazo[1,2-*b*]pyridazine **(61)** (93.7 mg, 0.403 mmol, 84% yield) as an off-white solid. MS (ESI+) *m/z* calcd for $C_6H_4BrClN_3^+$ [M + H]$^+$ 231.9, found 231.9. UPLC analysis (method A), 2.45 min, >80% purity. ^{1}H NMR (300 MHz, $CDCl_3$) δ 8.02 (d, *J* = 1.2 Hz, 1H), 7.85 (d, *J* = 1.2 Hz, 1H), 7.41 (s, 1H).

6-Chloro-8-((2,2-difluorocyclopropyl)methoxy)imidazo[1,2-*b*]pyridazine (62)

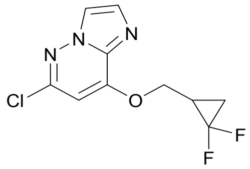

To a cooled (0 °C) and stirred suspension of sodium hydride (32.3 mg, 0.807 mmol) in THF (5.0 mL) was added (2,2-difluorocyclopropyl)methanol (38.4 mg, 0.355 mmol) and the reaction mixture stirred for 10 min until gas evolution ceased. 8-Bromo-6-chloroimidazo[1,2-*b*]pyridazine **(61)** (93.7 mg, 0.403 mmol) was then added and the reaction mixture stirred at RT for 1 h. The reaction was quenched with the addition of water, basified with sat. aq $NaHCO_3$ and extracted with EtOAc (×3). The combined

organic layers were washed with brine, dried (Na_2SO_4), filtered and concentrated under reduced pressure to give 6-chloro-8-((2,2-difluorocyclopropyl)methoxy)imidazo[1,2-*b*]pyridazine **(62)** (89 mg, 0.34 mmol, 85% yield) as an off-white solid. MS (ESI+) *m/z* calcd for $C_{10}H_9ClF_2N_3O^+$ $[M + H]^+$ 260.0, found 260.0. UPLC analysis (method A), 2.72 min, >98% purity. ^{1}H NMR (300 MHz, MeOD-d_4) δ 8.05 (d, *J* = 1.5 Hz, 1H), 7.65 (d, *J* = 1.5 Hz, 1H), 6.88 (s, 1H), 4.58 – 4.47 (m, 1H), 4.43 – 4.33 (m, 1H), 2.46 – 2.26 (m, 1H), 1.85 – 1.66 (m, 1H), 1.60 – 1.45 (m, 1H).

8-((2,2-Difluorocyclopropyl)methoxy)-*N*-(4-(4-isopropylpiperazin-1-yl)phenyl)imidazo[1,2-*b*]pyridazin-6-amine (34)

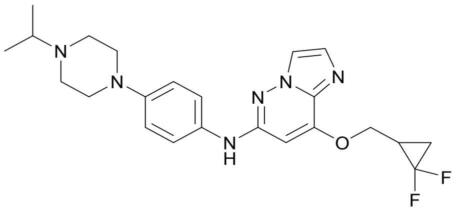

6-Chloro-8-((2,2-difluorocyclopropyl)methoxy)imidazo[1,2-*b*]pyridazine **(62)** (10 mg, 0.039 mmol) and 4-(4-isopropylpiperazin-1-yl)aniline (10.1 mg, 0.046 mmol) were reacted together according to general procedure B and heated thermally at 110 °C for 4 h. The reaction mixture was then cooled, placed on an SCX-II cartridge, washed with DCM and MeOH and eluted with methanolic ammonia. The basic fraction was concentrated under reduced pressure and purified by preparatory HPLC (0.1% NH_3 in MeCN/Water; 30-70%) to yield 8-((2,2-difluorocyclopropyl)methoxy)-*N*-(4-(4-isopropyl-piperazin-1-yl)phenyl)imidazo[1,2-*b*]pyridazin-6-amine **(34)** (3.8 mg, 0.009 mmol, 22%) as an off-white solid. MS (ESI+) *m/z* calcd for $C_{23}H_{29}F_2N_6O^+$ $[M + H]^+$ 443.2, found 443.3. UPLC analysis (method D), 2.76 min, 96% purity. 1H NMR (300 MHz, MeOD-d_4) δ 7.75 (d, *J* = 1.2 Hz, 1H), 7.62 – 7.50 (m, 2H), 7.38 (d, *J* = 1.2 Hz, 1H), 7.00 (d, *J* = 8.8 Hz, 2H), 6.25 – 6.18 (m, 1H), 4.31 (dt, *J* = 18.8, 8.8 Hz, 2H), 3.22 – 3.14 (m, 4H), 2.81 – 2.64 (m, 5H), 2.44 – 2.22 (m, 1H), 1.83 – 1.65 (m, 1H), 1.60 – 1.42 (m, 1H), 1.15 (d, *J* = 6.5 Hz, 6H). *Anilino N-H proton exchanged with MeOD.* 19F NMR (282 MHz, MeOD-d_4) δ –130.88 (d, *J* = 160.7 Hz), –144.96 (d, *J* = 160.9 Hz).

6-Chloro-4-((2,2-difluorocyclopropyl)methoxy)-1*H*-pyrazolo[3,4-*d*]pyrimidine (63)

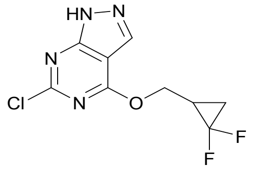

To a cooled (0 °C) and stirred solution of (2,2-difluorocyclopropyl)methanol (75.5 mg, 0.698 mmol) in THF (2.0 mL) was added sodium hydride (50.8 mg, 1.27 mmol) and the reaction mixture stirred for 5 min until the effervescence ceased. 4,6-Dichloro-1*H*-pyrazolo[3,4-*d*]pyrimidine (120 mg, 0.635 mmol) was then added and the reaction mixture stirred at RT for 16 h. After this time the reaction was quenched with the addition of water and extracted with EtOAc (×2). The organic layer was washed with brine, dried (Na_2SO_4), filtered and concentrated under reduced pressure. The crude product was purified by silica gel flash column chromatography (EtOAc/petroleum ether; 5-100%) to yield 6-chloro-4-((2,2-difluorocyclopropyl)methoxy)-1*H*-pyrazolo[3,4-*d*]pyrimidine **(63)** (90 mg, 0.345 mmol, 54% yield) as a white solid. MS (ESI+) *m/z* calcd for $C_9H_8ClF_2N_4O^+$ $[M + H]^+$ 261.0, found 261.0. UPLC analysis (method D), 2.73 min, 86% purity. ^{1}H NMR (300 MHz, $CDCl_3$) δ 8.15 (s, 1H), 4.82 – 4.68 (m, 1H), 4.68 – 4.53 (m, 1H), 2.32 – 2.13 (m, 1H), 1.72 – 1.56 (m, 1H), 1.49 – 1.30 (m, 1H). *N-H proton unresolved.* ^{19}F NMR (282 MHz, $CDCl_3$) δ –129.23 (d, J = 161.4 Hz), –142.96 (d, J = 161.4 Hz).

4-((2,2-Difluorocyclopropyl)methoxy)-*N*-(4-(4-isopropylpiperazin-1-yl)phenyl)-1*H*-pyrazolo[3,4-*d*]pyrimidin-6-amine (35)

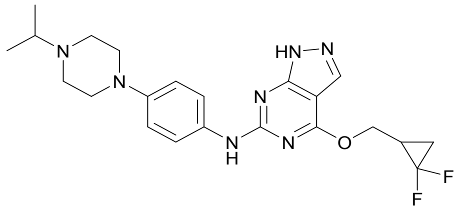

6-Chloro-4-((2,2-difluorocyclopropyl)methoxy)-1*H*-pyrazolo[3,4-*d*]pyrimidine **(63)** (45.0 mg, 0.170 mmol), 4-(4-isopropylpiperazin-1-yl)aniline (45.0 mg, 0.210 mmol), *p*-toluenesulfonic acid monohydrate (66.0 mg, 0.350 mmol) were taken up in 2-butanol (2.0 mL) and heated at 100 °C under µW irradiation for 3 h. The reaction mixture was quenched with sat. aq $NaHCO_3$ and extracted with EtOAc (×2). The organic layer was washed with brine, dried (Na_2SO_4), filtered and concentrated under reduced pressure. The crude material was partially purified by silica gel flash column chromatography (EtOAc/petroleum ether; 5-100%, followed by MeOH/DCM; 0-20%), followed by further purification by preparatory HPLC (0.1% NH_3 in MeCN/Water; 30-70%) to yield 4-((2,2-difluorocyclopropyl) methoxy)-*N*-(4-(4-isopropylpiperazin-1-yl)phenyl)-1*H*-pyrazolo[3,4-*d*]pyrimidin-6-amine **(35)** (28.8 mg, 0.065 mmol, 38% yield) as a white solid. MS (ESI+) *m/z* calcd for $C_{22}H_{28}F_2N_7O^+$ $[M + H]^+$

443.2, found 443.3. UPLC analysis (method D), 2.77 min, >98% purity. ^{1}H NMR (300 MHz, DMSO-d_6) δ 13.17 (s, 1H), 9.31 (s, 1H), 7.91 (s, 1H), 7.61 (d, *J* = 8.9 Hz, 2H), 6.88 (d, *J* = 9.1 Hz, 2H), 4.73 – 4.59 (m, 1H), 4.51 – 4.37 (m, 1H), 3.11 – 2.96 (m, 4H), 2.66 (p, *J* = 6.5 Hz, 1H), 2.61 – 2.54 (m, 4H), 2.46 – 2.29 (m, 1H), 1.85 – 1.68 (m, 1H), 1.65 – 1.50 (m, 1H), 1.00 (d, *J* = 6.5 Hz, 6H). ^{19}F NMR (282 MHz, DMSO-d_6) δ –127.47 (d, *J* = 156.4 Hz), –141.40 (d, *J* = 156.3 Hz).

NMR spectra and LC-MS traces of selected compounds

Compound 14

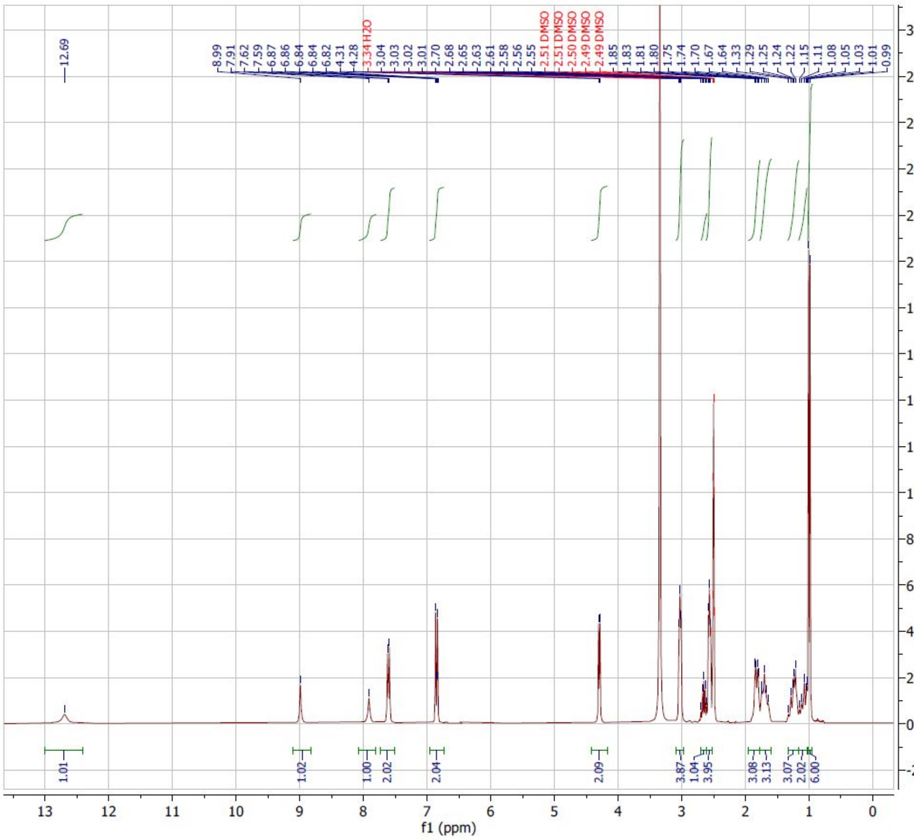

	Parameter	Value
1	Title	GGA5-031-2.10.fid
2	Origin	Bruker BioSpin GmbH
3	Solvent	DMSO
4	Temperature	295.8
5	Pulse Sequence	zg30
6	Experiment	1D
7	Probe	5 mm PABBO BB-1H/ D Z-GRD Z104275/ 0242
8	Number of Scans	16
9	Receiver Gain	191.9
10	Relaxation Delay	1.0000
11	Pulse Width	14.0000
12	Presaturation Frequency	
13	Modification Date	2020-10-30T12:18:36
14	Spectrometer Frequency	300.13
15	Spectral Width	6009.6
16	Lowest Frequency	-1154.0
17	Nucleus	1H
18	Acquired Size	32768
19	Spectral Size	65536
20	Digital Resolution	0.09

^{1}H NMR (300 MHz, DMSO) δ 12.69 (s, 1H), 8.99 (s, 1H), 7.91 (s, 1H), 7.60 (d, *J* = 8.6 Hz, 2H), 6.85 (d, *J* = 9.1 Hz, 2H), 4.30 (d, *J* = 6.3 Hz, 2H), 3.09 – 2.97 (m, 4H), 2.65 (p, *J* = 6.5 Hz, 1H), 2.60 – 2.53 (m, 4H), 1.96 – 1.77 (m, 3H), 1.77 – 1.59 (m, 3H), 1.36 – 1.16 (m, 3H), 1.16 – 1.03 (m, 2H), 1.00 (d, *J* = 6.5 Hz, 6H).

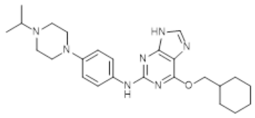

Column Name ACQUITY UPLC® HSS C18 1.8µm

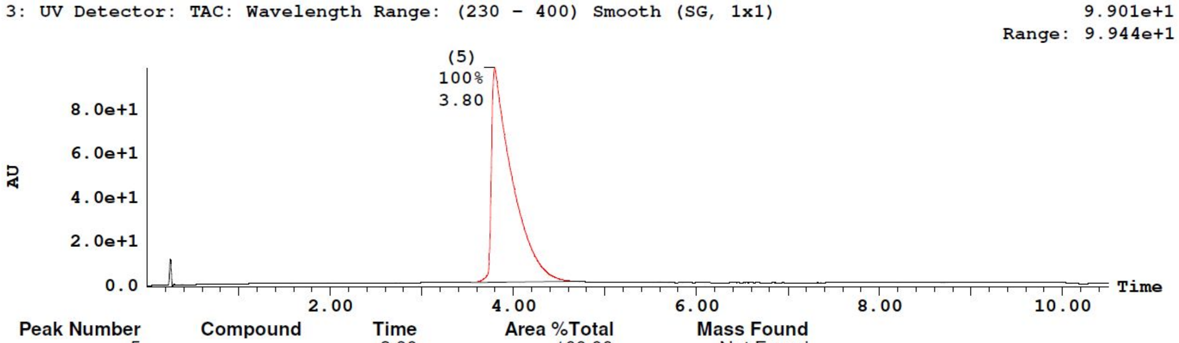

Peak Number	Compound	Time	Area %Total	Mass Found
5		3.80	100.00	Not Found

Peak ID	Compound	Time	Mass Found
5		3.78	Not Found

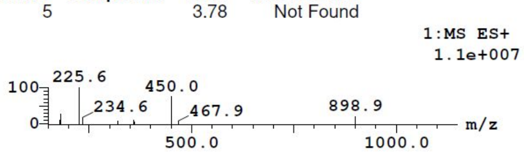

Peak ID	Compound	Time	Mass Found
5		3.78	Not Found

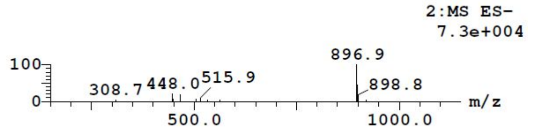

Compound 19

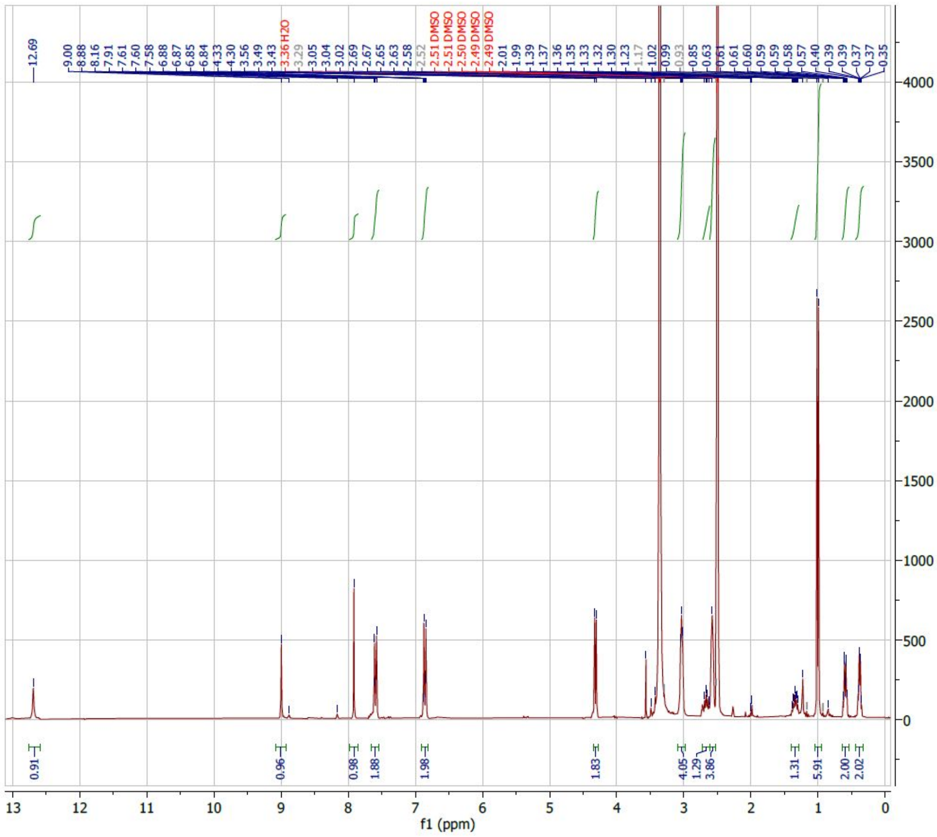

Parameter	Value
1 Title	GGA6-007-6.10.fid
2 Origin	Bruker BioSpin GmbH
3 Solvent	DMSO
4 Temperature	294.5
5 Pulse Sequence	zg30
6 Experiment	1D
7 Probe	5 mm PABBO BB-1H/ D Z-GRD Z104275/ 0242
8 Number of Scans	64
9 Receiver Gain	191.9
10 Relaxation Delay	1.0000
11 Pulse Width	14.0000
12 Presaturation Frequency	
13 Modification Date	2021-05-21T11:13:44
14 Spectrometer Frequency	300.13
15 Spectral Width	6009.6
16 Lowest Frequency	-1154.1
17 Nucleus	1H
18 Acquired Size	32768
19 Spectral Size	65536
20 Digital Resolution	

^{1}H NMR (300 MHz, DMSO) δ 12.69 (s, 1H), 9.00 (s, 1H), 7.91 (s, 1H), 7.59 (d, J = 9.0 Hz, 2H), 6.86 (d, J = 9.0 Hz, 2H), 4.32 (d, J = 7.3 Hz, 2H), 3.10 – 2.98 (m, 4H), 2.72 – 2.61 (m, 1H), 2.61 – 2.53 (m, 4H), 1.41 – 1.29 (m, 1H), 1.01 (d, J = 6.5 Hz, 6H), 0.64 – 0.54 (m, 2H), 0.44 – 0.32 (m, 2H).

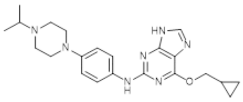

Column Name ACQUITY UPLC® HSS C18 1.8μm

3: UV Detector: TAC: Wavelength Range: (210 - 400) Smooth (SG, 1x1) 9.721e+1

Range: 9.757e+1

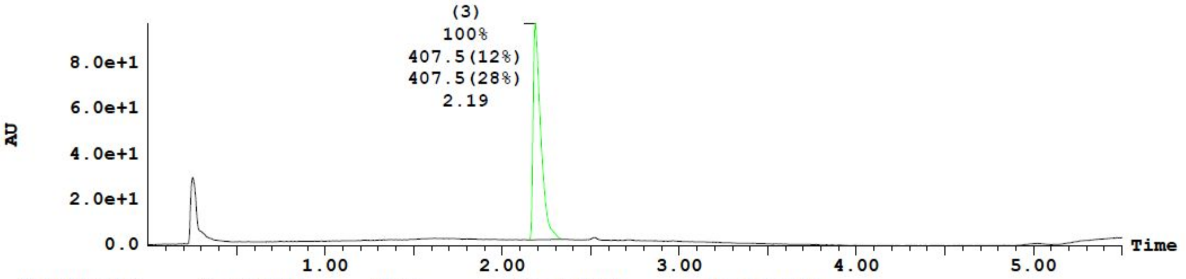

Peak Number	Compound	Time	Area %Total	Mass Found
3	Tentative	2.19	100.00	407.5000, 407.5000

Peak ID	Compound	Time	Mass Found
3	Tentative	2.19	408

1:MS ES+
2.8e+007

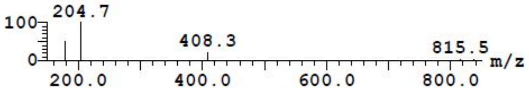

Peak ID	Compound	Time	Mass Found
3	Found	2.19	406

2:MS ES-
1.6e+004

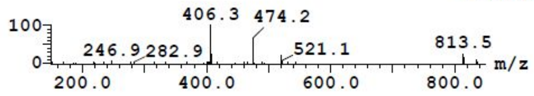

Compound 20

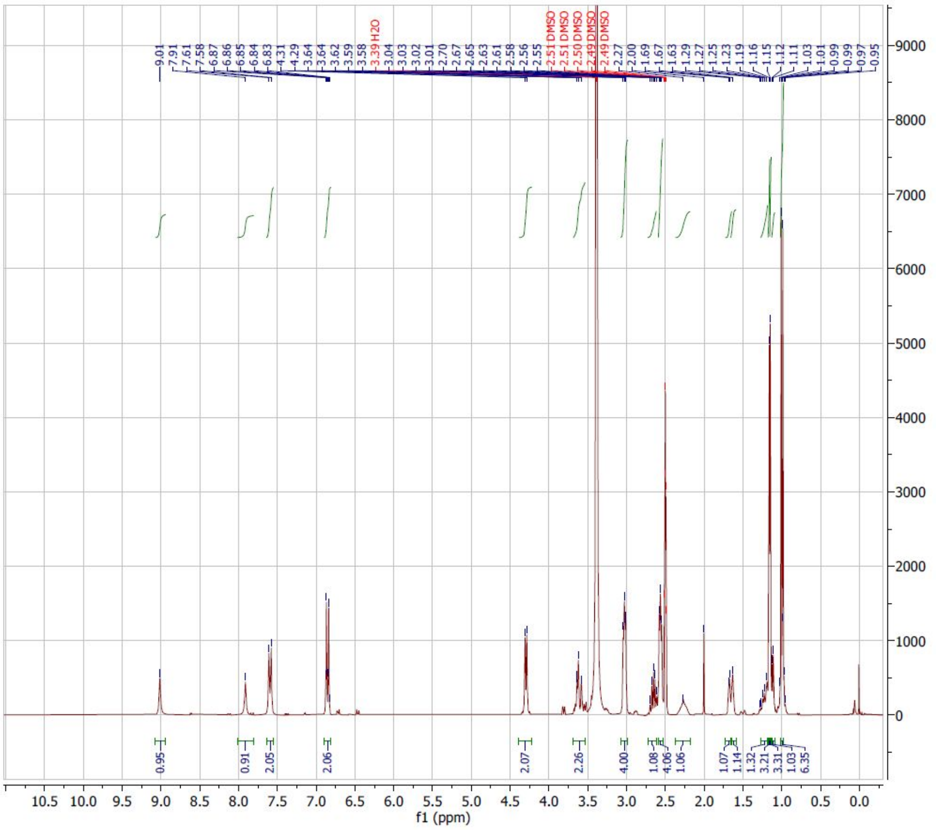

Parameter		Value
1	Title	GGA5-053-2.10.fid
2	Origin	Bruker BioSpin GmbH
3	Solvent	DMSO
4	Temperature	293.8
5	Pulse Sequence	zg30
6	Experiment	1D
7	Probe	5 mm PABBO BB-1H/ D Z-GRD Z104275/ 0242
8	Number of Scans	64
9	Receiver Gain	191.9
10	Relaxation Delay	1.0000
11	Pulse Width	14.0000
12	Presaturation Frequency	
13	Modification Date	2021-01-25T15:58:12
14	Spectrometer Frequency	300.13
15	Spectral Width	6009.6
16	Lowest Frequency	-1153.8
17	Nucleus	1H
18	Acquired Size	32768
19	Spectral Size	65536
20	Digital Resolution	

^{1}H NMR (300 MHz, DMSO) δ 9.01 (s, 1H), 7.91 (s, 1H), 7.59 (d, J = 8.6 Hz, 2H), 6.85 (d, J = 9.1 Hz, 2H), 4.30 (d, J = 6.4 Hz, 2H), 3.73 – 3.51 (m, 2H), 3.09 – 2.97 (m, 4H), 2.73 – 2.62 (m, 1H), 2.61 – 2.53 (m, 4H), 2.37 – 2.18 (m, 1H), 1.68 (s, 1H), 1.63 (s, 1H), 1.27 – 1.17 (m, 1H), 1.16 (s, 3H), 1.15 (s, 3H), 1.13 – 1.07 (m, 1H), 1.00 (d, J = 6.5 Hz, 6H).

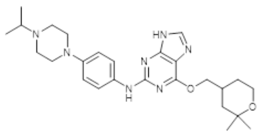

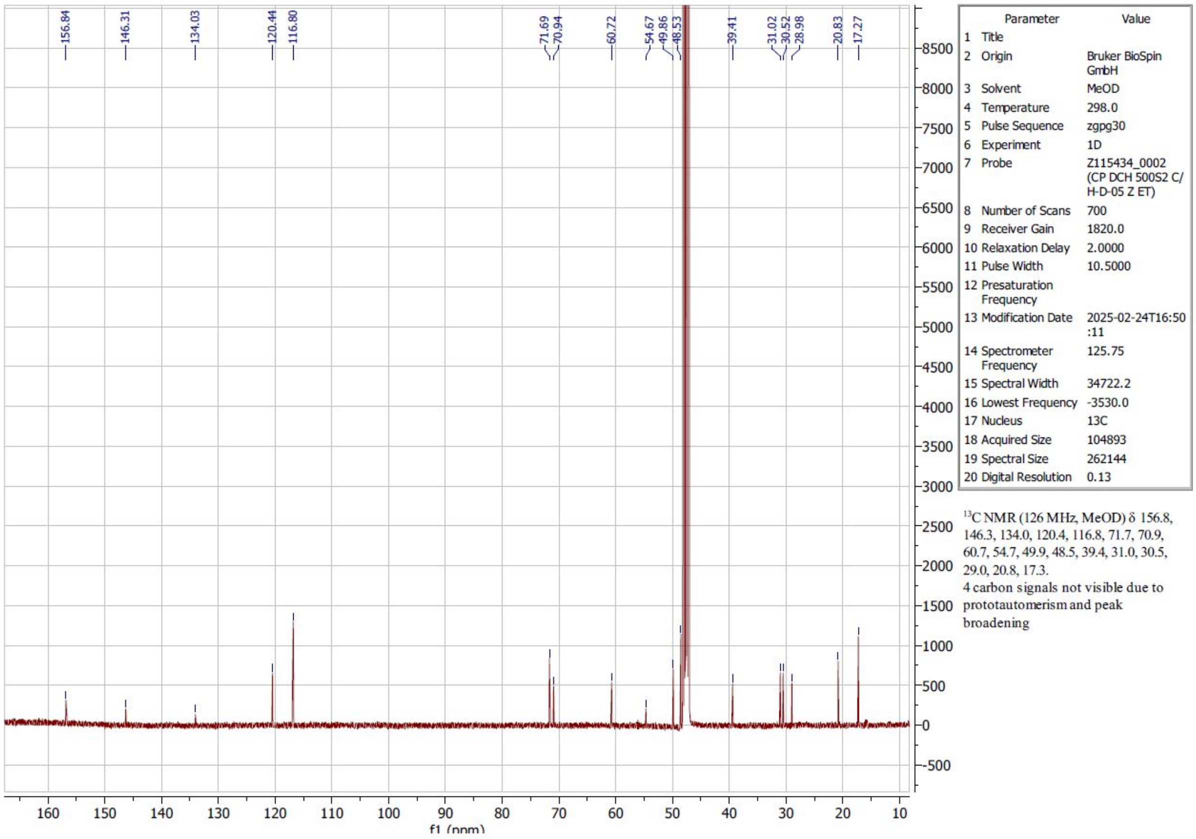

Parameter		Value
1	Title	
2	Origin	Bruker BioSpin GmbH
3	Solvent	MeOD
4	Temperature	298.0
5	Pulse Sequence	zgpg30
6	Experiment	1D
7	Probe	Z115434_0002 (CP DCH 500S2 C/ H-D-05 Z ET)
8	Number of Scans	700
9	Receiver Gain	1820.0
10	Relaxation Delay	2.0000
11	Pulse Width	10.5000
12	Presaturation Frequency	
13	Modification Date	2025-02-24T16:50:11
14	Spectrometer Frequency	125.75
15	Spectral Width	34722.2
16	Lowest Frequency	-3530.0
17	Nucleus	13C
18	Acquired Size	104893
19	Spectral Size	262144
20	Digital Resolution	0.13

^{13}C NMR (126 MHz, MeOD) δ 156.8, 146.3, 134.0, 120.4, 116.8, 71.7, 70.9, 60.7, 54.7, 49.9, 48.5, 39.4, 31.0, 30.5, 29.0, 20.8, 17.3.
4 carbon signals not visible due to prototautomerism and peak broadening

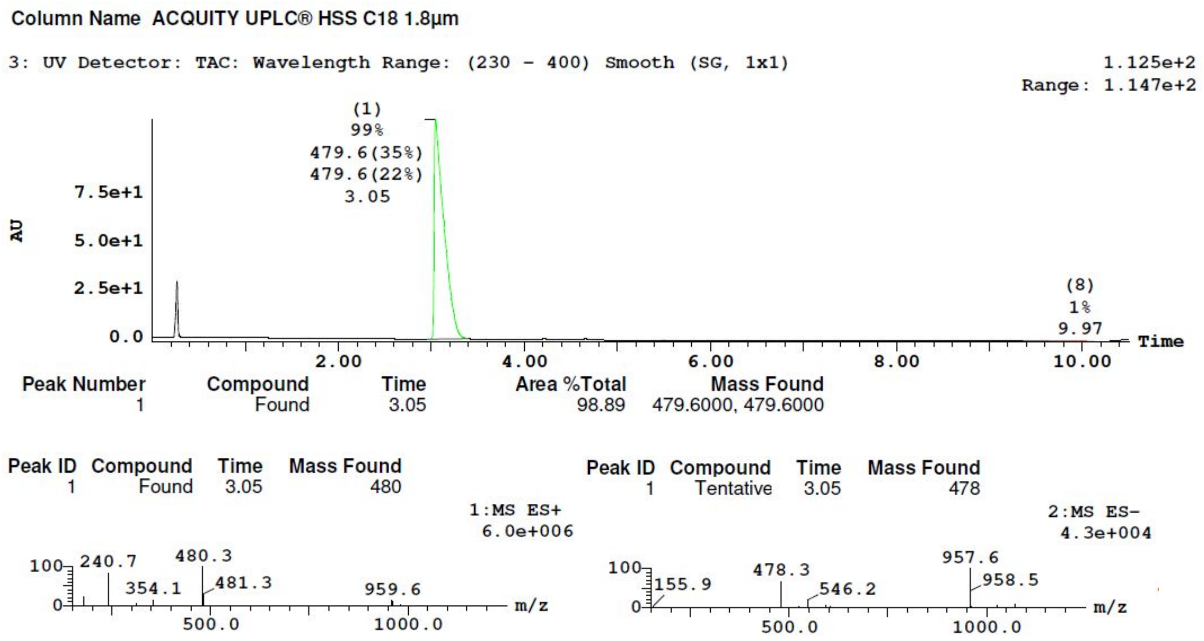

Compound 21

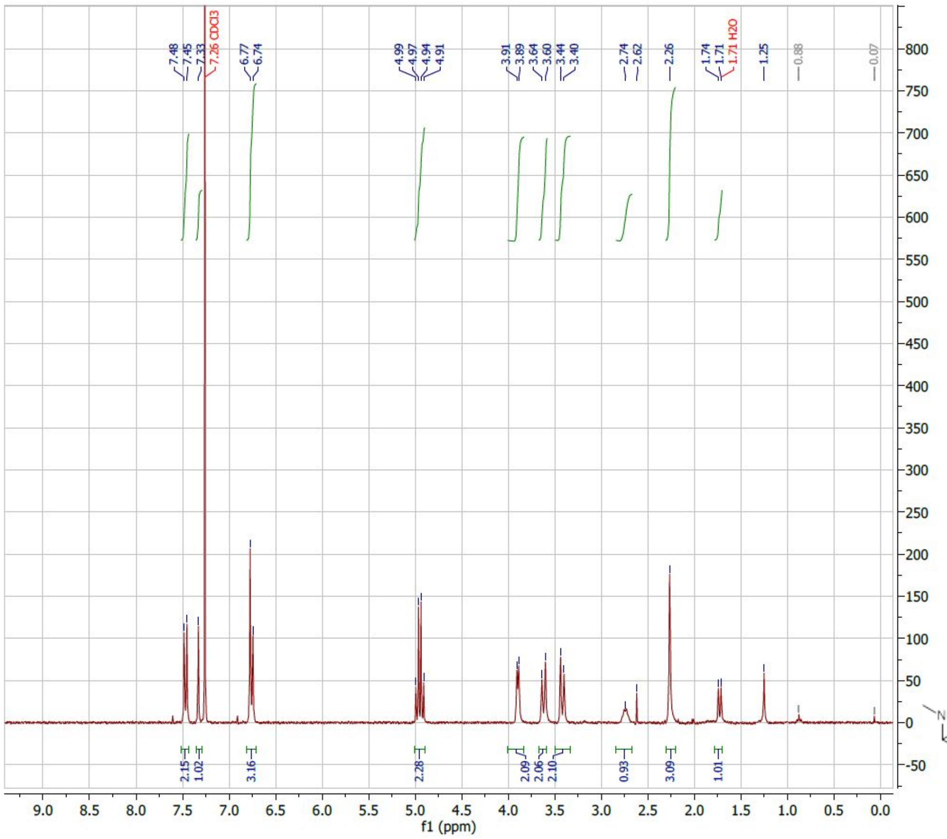

	Parameter	Value
1	Title	GGA4-040-3.10.fid
2	Origin	Bruker BioSpin GmbH
3	Solvent	CDCl3
4	Temperature	293.9
5	Pulse Sequence	zg30
6	Experiment	1D
7	Probe	5 mm PABBO BB-1H/ D Z-GRD Z104275/ 0242
8	Number of Scans	16
9	Receiver Gain	191.9
10	Relaxation Delay	1.0000
11	Pulse Width	14.0000
12	Presaturation Frequency	
13	Modification Date	2019-12-16T12:39:32
14	Spectrometer Frequency	300.13
15	Spectral Width	6009.6
16	Lowest Frequency	-1158.8
17	Nucleus	1H
18	Acquired Size	32768
19	Spectral Size	65536
20	Digital Resolution	

^{1}H NMR (300 MHz, $CDCl_3$) δ 7.47 (d, *J* = 8.9 Hz, 2H), 7.33 (s, 1H), 6.81 – 6.71 (m, 3H), 4.95 (q, *J* = 8.4 Hz, 2H), 3.90 (d, *J* = 6.0 Hz, 2H), 3.62 (d, *J* = 11.2 Hz, 2H), 3.42 (d, *J* = 11.1 Hz, 2H), 2.84 – 2.67 (m, 1H), 2.26 (s, 3H), 1.73 (d, *J* = 8.6 Hz, 1H).

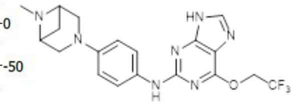

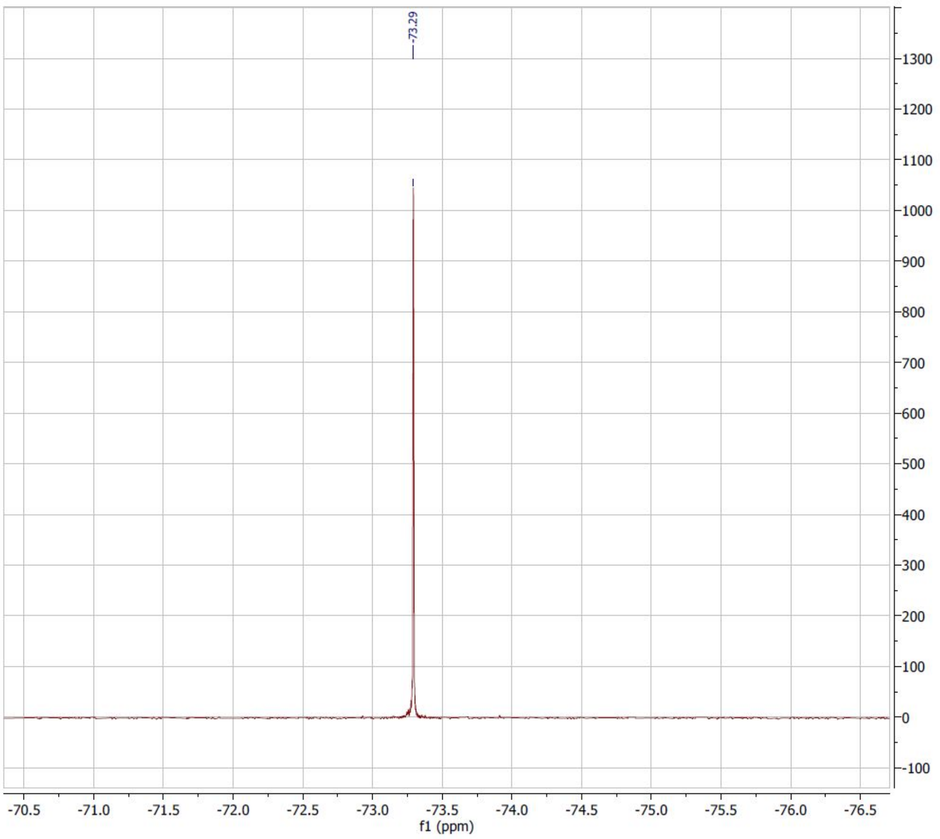

	Parameter	Value
1	Title	GGA4-040-3.11.fid
2	Origin	Bruker BioSpin GmbH
3	Solvent	CDCl3
4	Temperature	294.0
5	Pulse Sequence	zgfhigqn.2
6	Experiment	1D
7	Probe	5 mm PABBO BB-1H/ D Z-GRD Z104275/ 0242
8	Number of Scans	16
9	Receiver Gain	191.9
10	Relaxation Delay	1.0000
11	Pulse Width	15.0000
12	Presaturation Frequency	
13	Modification Date	2019-12-16T12:42:44
14	Spectrometer Frequency	282.38
15	Spectral Width	66964.3
16	Lowest Frequency	-61722.4
17	Nucleus	19F
18	Acquired Size	65536
19	Spectral Size	131072
20	Digital Resolution	0.51

^{19}F NMR (282 MHz, $CDCl_3$) δ -73.29.

Column Name ACQUITY UPLC® HSS C18 1.8µm

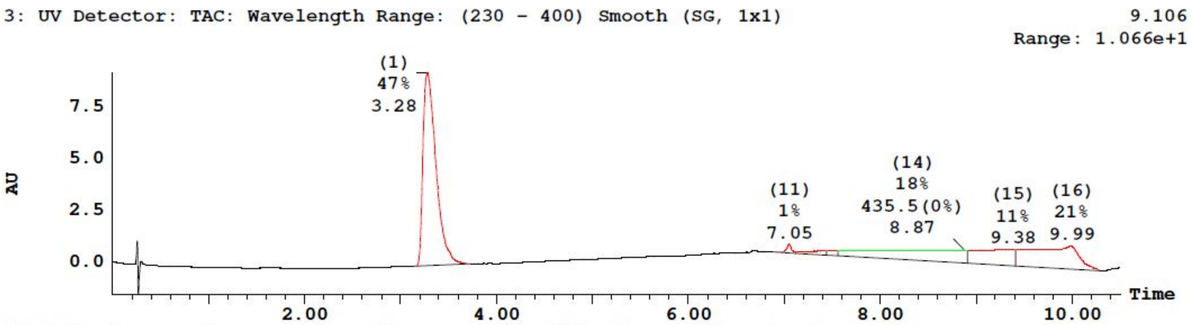

Peak Number	Compound	Time	Area %Total	Mass Found
1		3.28	46.98	Not Found

Peak ID	Compound	Time	Mass Found
1		3.28	Not Found

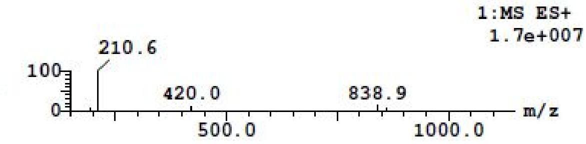

Peak ID	Compound	Time	Mass Found
1		3.28	Not Found

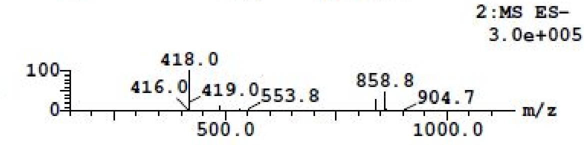

Compound 22

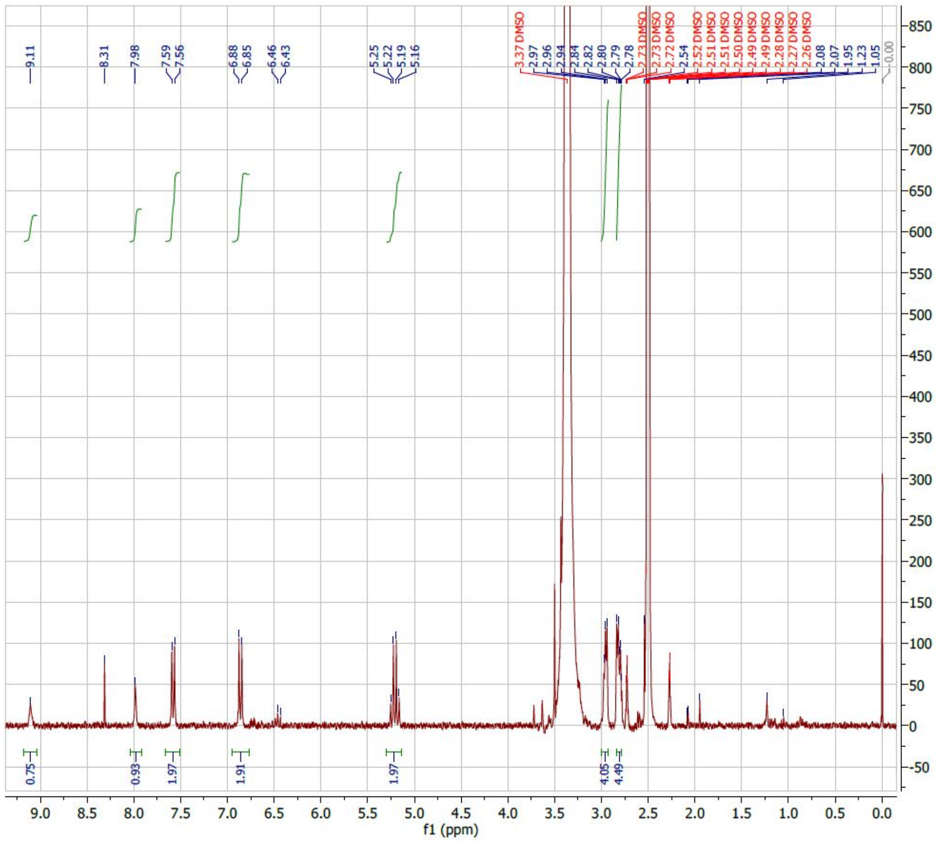

	Parameter	Value
1	Title	GGA4-024-2.10.fid
2	Origin	Bruker BioSpin GmbH
3	Solvent	DMSO
4	Temperature	293.8
5	Pulse Sequence	zg30
6	Experiment	1D
7	Probe	5 mm PABBO BB-1H/ D Z-GRD Z104275/ 0242
8	Number of Scans	128
9	Receiver Gain	191.9
10	Relaxation Delay	1.0000
11	Pulse Width	14.0000
12	Presaturation Frequency	
13	Modification Date	2019-11-15T10:59:58
14	Spectrometer Frequency	300.13
15	Spectral Width	6009.6
16	Lowest Frequency	-1153.8
17	Nucleus	1H
18	Acquired Size	32768
19	Spectral Size	65536
20	Digital Resolution	

^{1}H NMR (300 MHz, DMSO) δ 9.11 (s, 1H), 7.98 (s, 1H), 7.58 (d, J = 9.0 Hz, 2H), 6.86 (d, J = 9.0 Hz, 2H), 5.21 (q, J = 9.1 Hz, 2H), 3.00 – 2.92 (m, 4H), 2.84 – 2.78 (m, 4H).

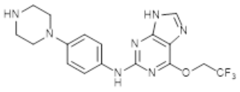

Column Name ACQUITY UPLC® HSS C18 1.8µm

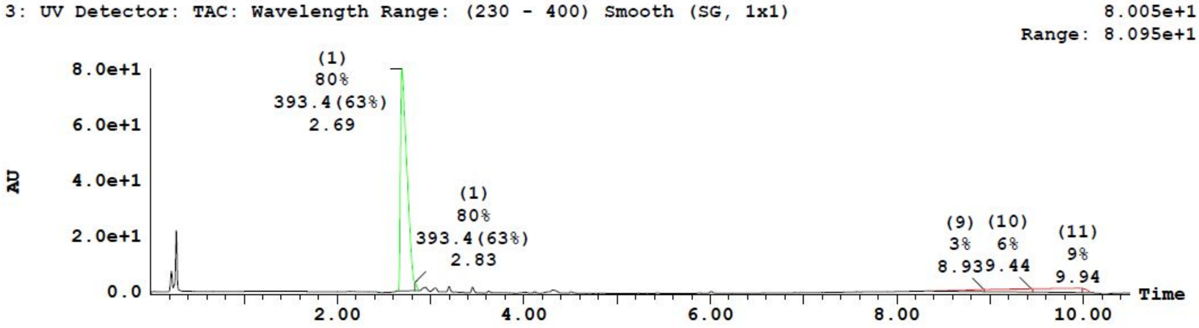

Peak ID	Compound	Time	Mass Found
1		2.82	Not Found

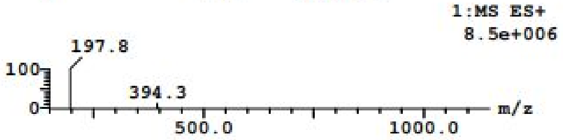

Peak ID	Compound	Time	Mass Found
1		2.69	Not Found

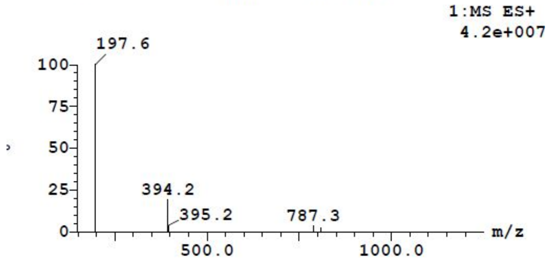

Compound 23

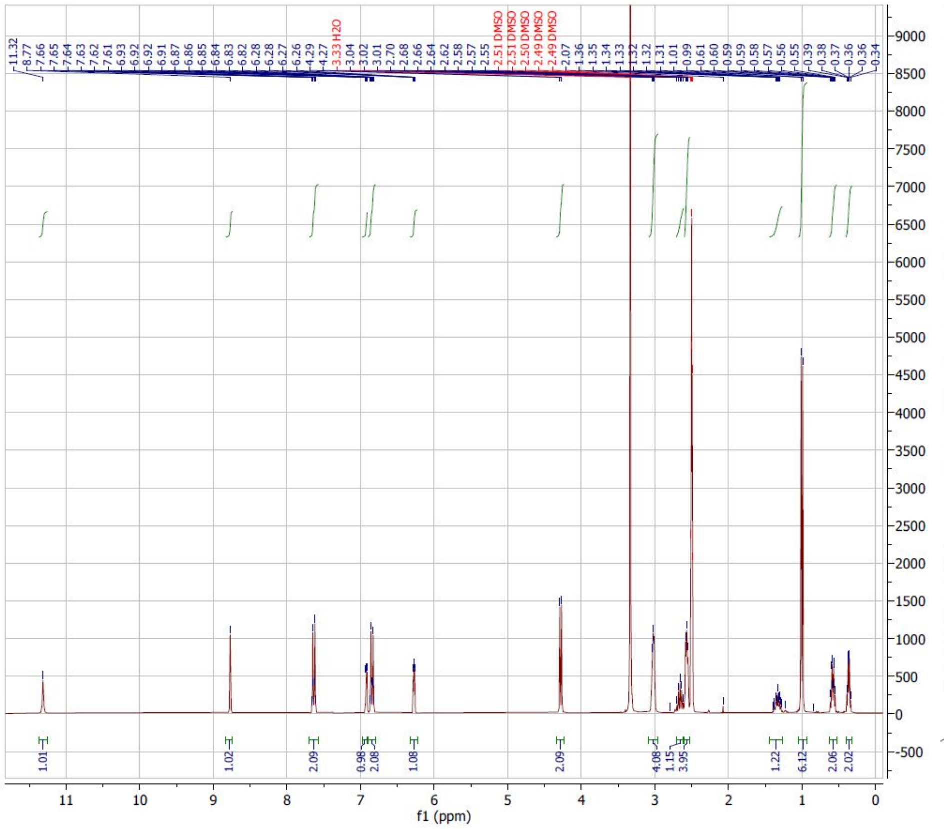

	Parameter	Value
1	Title	HKB7-078-3.10.fid
2	Origin	Bruker BioSpin GmbH
3	Solvent	DMSO
4	Temperature	296.1
5	Pulse Sequence	zg30
6	Experiment	1D
7	Probe	5 mm PABBO BB-1H/ D Z-GRD Z104275/ 0242
8	Number of Scans	64
9	Receiver Gain	191.9
10	Relaxation Delay	1.0000
11	Pulse Width	14.0000
12	Presaturation Frequency	
13	Modification Date	2020-10-19T16:55:12
14	Spectrometer Frequency	300.13
15	Spectral Width	6009.6
16	Lowest Frequency	-1154.0
17	Nucleus	1H
18	Acquired Size	32768
19	Spectral Size	65536
20	Digital Resolution	

^{1}H NMR (300 MHz, DMSO) δ 11.32 (s, 1H), 8.77 (s, 1H), 7.63 (d, *J* = 9.0 Hz, 2H), 6.92 (dd, *J* = 3.5, 2.2 Hz, 1H), 6.84 (d, *J* = 9.1 Hz, 2H), 6.27 (dd, *J* = 3.5, 1.9 Hz, 1H), 4.28 (d, *J* = 7.2 Hz, 2H), 3.08 – 2.96 (m, 4H), 2.71 – 2.61 (m, 1H), 2.60 – 2.53 (m, 4H), 1.41 – 1.27 (m, 1H), 1.00 (d, *J* = 6.5 Hz, 6H), 0.63 – 0.53 (m, 2H), 0.41 – 0.32 (m, 2H).

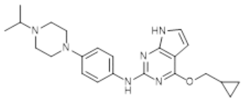

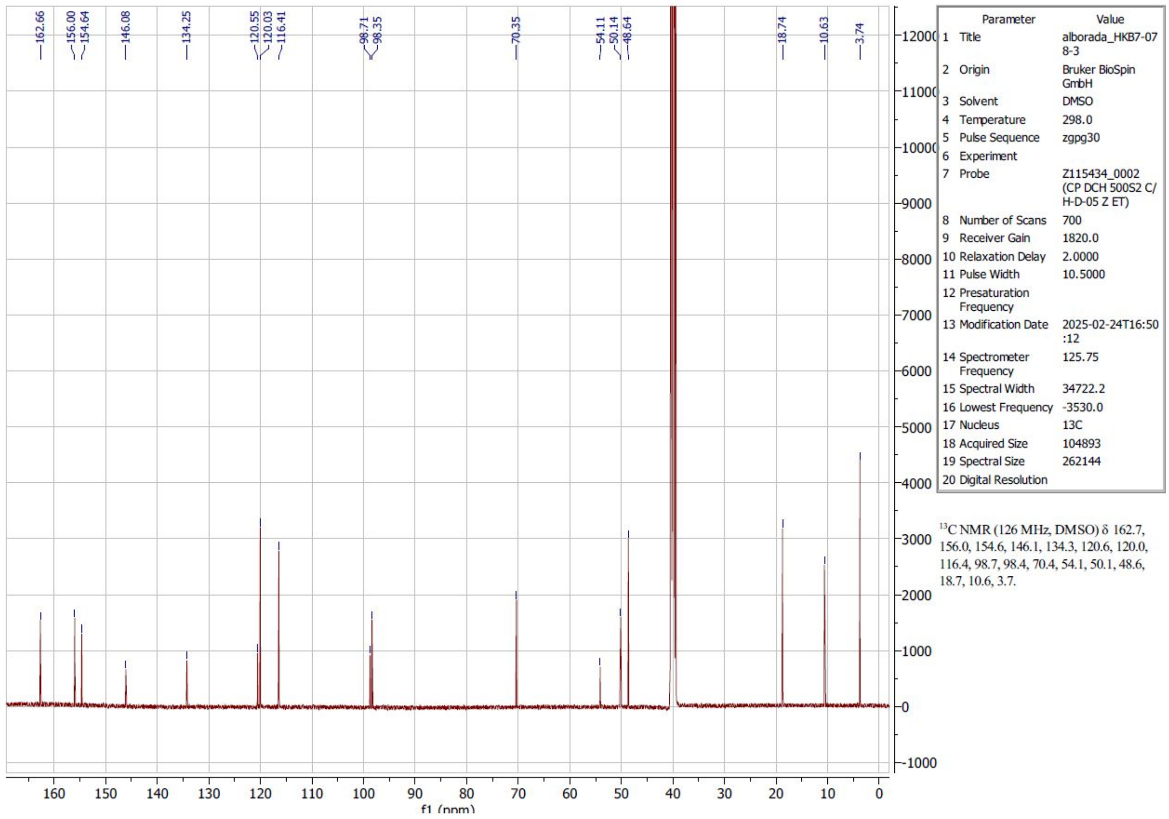

	Parameter	Value
1	Title	alborada_HKB7-078-3
2	Origin	Bruker BioSpin GmbH
3	Solvent	DMSO
4	Temperature	298.0
5	Pulse Sequence	zgpg30
6	Experiment	
7	Probe	Z115434_0002 (CP DCH 500S2 C/H-D-05 Z ET)
8	Number of Scans	700
9	Receiver Gain	1820.0
10	Relaxation Delay	2.0000
11	Pulse Width	10.5000
12	Presaturation Frequency	
13	Modification Date	2025-02-24T16:50:12
14	Spectrometer Frequency	125.75
15	Spectral Width	34722.2
16	Lowest Frequency	-3530.0
17	Nucleus	13C
18	Acquired Size	104893
19	Spectral Size	262144
20	Digital Resolution	

^{13}C NMR (126 MHz, DMSO) δ 162.7, 156.0, 154.6, 146.1, 134.3, 120.6, 120.0, 116.4, 98.7, 98.4, 70.4, 54.1, 50.1, 48.6, 18.7, 10.6, 3.7.

Column Name ACQUITY UPLC® HSS C18 1.8μm

3: UV Detector: TAC: Wavelength Range: (230 - 400) Smooth (SG, 1x1) 2.149e+1 Range: 2.27e+1

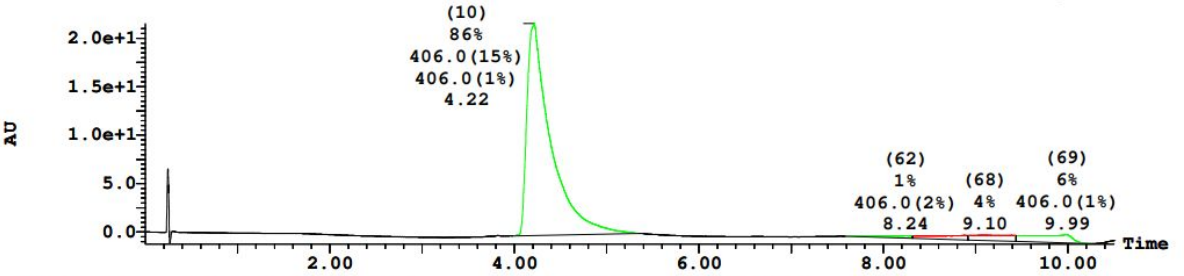

Peak Number	Compound	Time	Area %Total	Mass Found
10	Tentative	4.22	86.05	406.0000, 406.0000

Peak ID	Compound	Time	Mass Found
10	Tentative	4.23	407

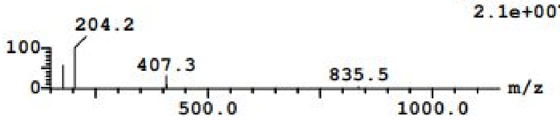

Compound 25

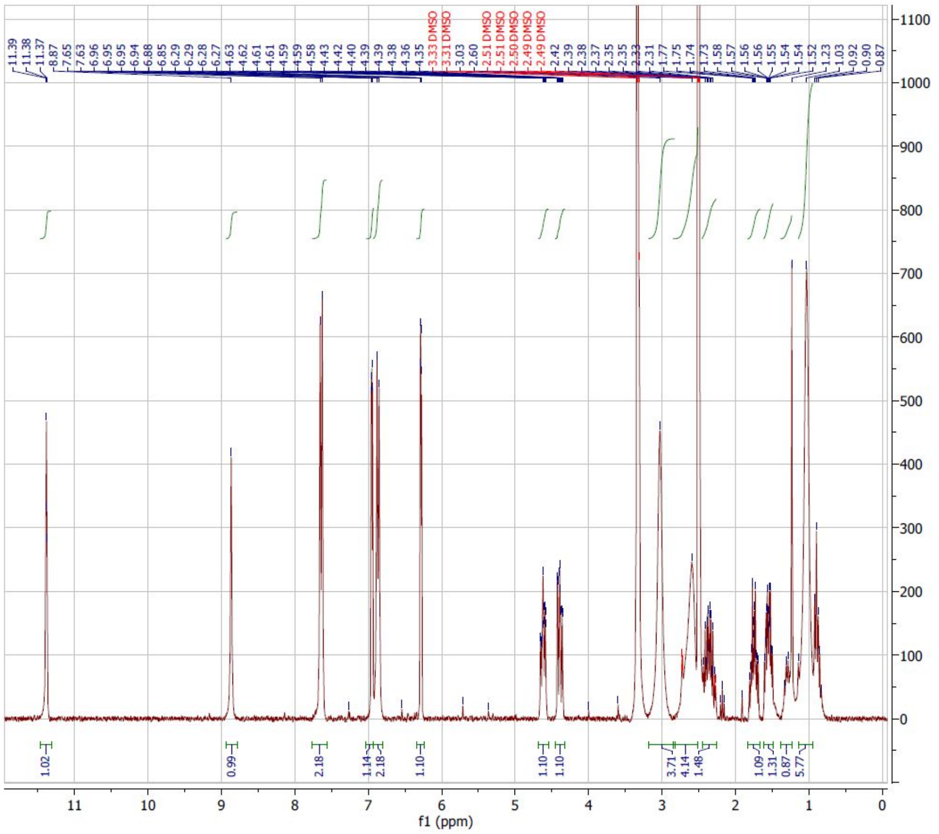

	Parameter	Value
1	Title	GGA6-034-2.10.fid
2	Origin	Bruker BioSpin GmbH
3	Solvent	DMSO
4	Temperature	295.3
5	Pulse Sequence	zg30
6	Experiment	1D
7	Probe	5 mm PABBO BB-1H/ D Z-GRD Z104275/ 0242
8	Number of Scans	128
9	Receiver Gain	191.9
10	Relaxation Delay	1.0000
11	Pulse Width	14.0000
12	Presaturation Frequency	
13	Modification Date	2021-06-09T10:53:28
14	Spectrometer Frequency	300.13
15	Spectral Width	6009.6
16	Lowest Frequency	-1154.0
17	Nucleus	1H
18	Acquired Size	32768
19	Spectral Size	65536
20	Digital Resolution	

^{1}H NMR (300 MHz, DMSO) δ 11.38 (s, 1H), 8.87 (s, 1H), 7.64 (d, J = 8.6 Hz, 2H), 6.95 (dd, J = 3.5, 2.2 Hz, 1H), 6.87 (d, J = 8.6 Hz, 2H), 6.28 (dd, J = 3.5, 1.9 Hz, 1H), 4.68 – 4.55 (m, 1H), 4.46 – 4.32 (m, 1H), 3.03 (s, 4H), 2.60 (s, 4H), 2.45 – 2.26 (m, 1H), 1.84 – 1.66 (m, 1H), 1.65 – 1.45 (m, 1H), 1.39 – 1.23 (m, 1H), 1.03 (s, 6H).

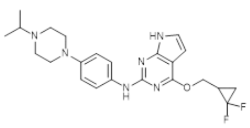

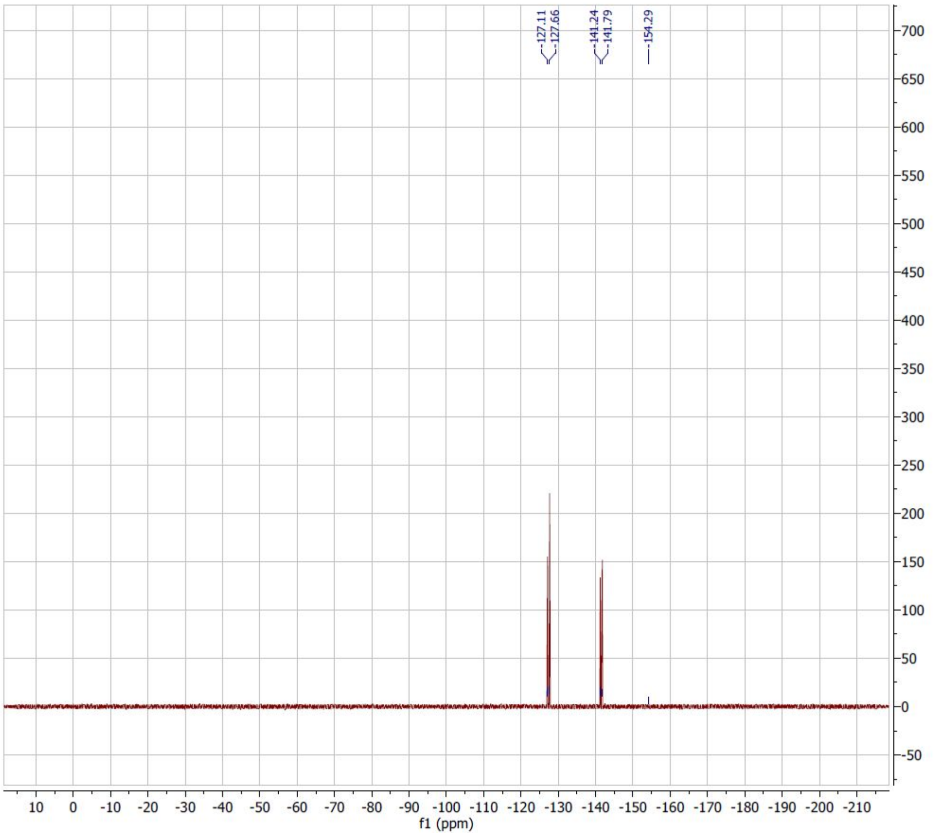

	Parameter	Value
1	Title	GGA6-034-2.11.fid
2	Origin	Bruker BioSpin GmbH
3	Solvent	DMSO
4	Temperature	295.4
5	Pulse Sequence	zgfhigqn.2
6	Experiment	1D
7	Probe	5 mm PABBO BB-1H/ D Z-GRD Z104275/ 0242
8	Number of Scans	16
9	Receiver Gain	191.9
10	Relaxation Delay	1.0000
11	Pulse Width	15.0000
12	Presaturation Frequency	
13	Modification Date	2021-06-09T10:56:26
14	Spectrometer Frequency	282.38
15	Spectral Width	66964.3
16	Lowest Frequency	-61722.4
17	Nucleus	19F
18	Acquired Size	65536
19	Spectral Size	131072
20	Digital Resolution	

^{19}F NMR (282 MHz, DMSO) δ -127.39 (d, *J* = 156.3 Hz), -141.52 (d, *J* = 156.0 Hz).

Column Name ACQUITY UPLC® HSS C18 1.8µm

3: UV Detector: TAC: Wavelength Range: (230 - 400) Smooth (SG, 1x1) 2.212e+1 Range: 2.486e+1

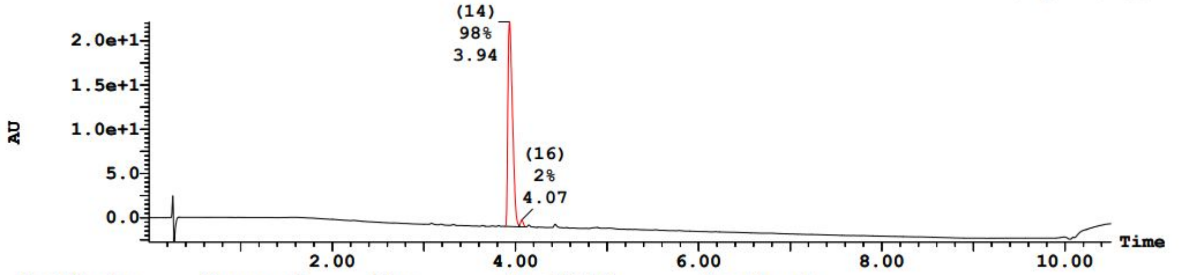

Peak Number	Compound	Time	Area %Total	Mass Found
14		3.94	98.02	Not Found
16		4.07	1.98	Not Found

Peak ID	Compound	Time	Mass Found
14		3.94	Not Found

1:MS ES+ 1.8e+007

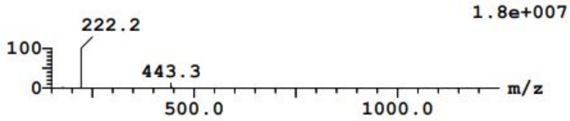

Compound 26

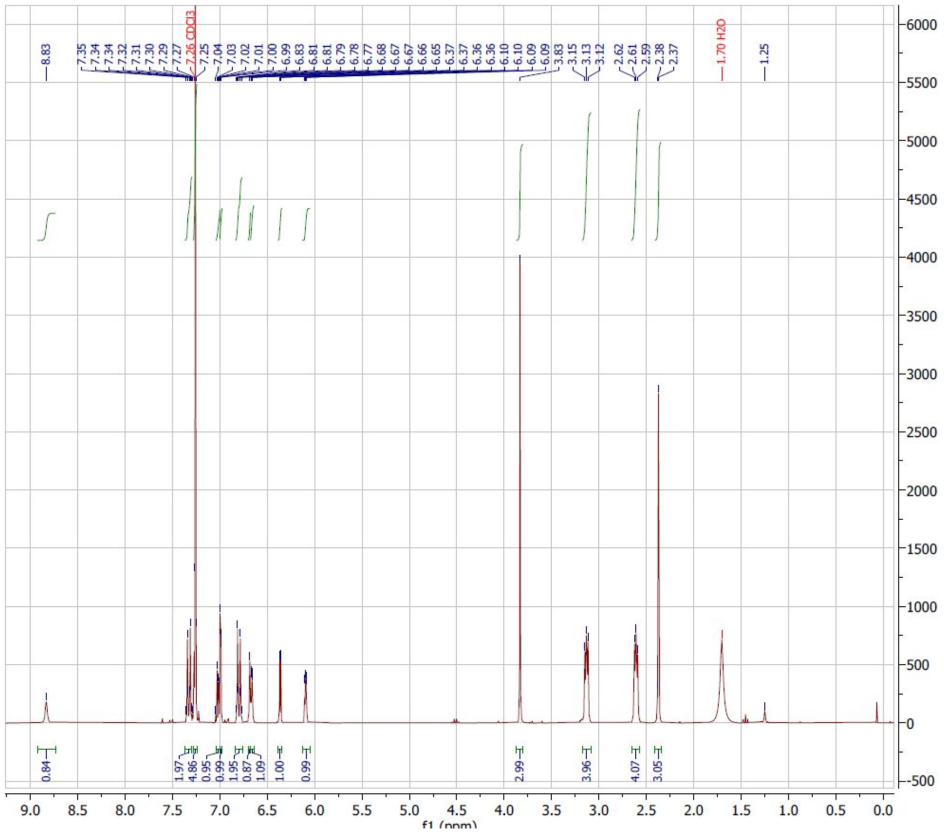

Parameter		Value
1	Title	HKB6-066-4.10.fid
2	Origin	Bruker BioSpin GmbH
3	Solvent	CDCl3
4	Temperature	295.3
5	Pulse Sequence	zg30
6	Experiment	1D
7	Probe	5 mm PABBO BB-1H/ D Z-GRD Z104275/ 0242
8	Number of Scans	64
9	Receiver Gain	191.9
10	Relaxation Delay	1.0000
11	Pulse Width	14.0000
12	Presaturation Frequency	
13	Modification Date	2025-07-03T09:42:11
14	Spectrometer Frequency	300.13
15	Spectral Width	6009.6
16	Lowest Frequency	-1158.7
17	Nucleus	1H
18	Acquired Size	32768
19	Spectral Size	65536
20	Digital Resolution	0.09

^{1}H NMR (300 MHz, $CDCl_3$) δ 8.83 (s, 1H), 7.37 – 7.30 (m, 2H), 7.28 – 7.24 (m, 2H), 7.04 – 7.00 (m, 1H), 6.99 (d, J = 3.3 Hz, 1H), 6.84 – 6.76 (m, 2H), 6.68 (s, 1H), 6.66 (dd, J = 3.6, 2.1 Hz, 1H), 6.36 (dd, J = 3.2, 0.6 Hz, 1H), 6.09 (dd, J = 3.6, 2.1 Hz, 1H), 3.83 (s, 3H), 3.17 – 3.08 (m, 4H), 2.66 – 2.57 (m, 4H), 2.37 (s, 3H).

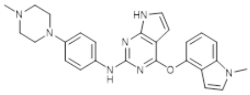

Column Name ACQUITY UPLC® HSS C18 1.8µm

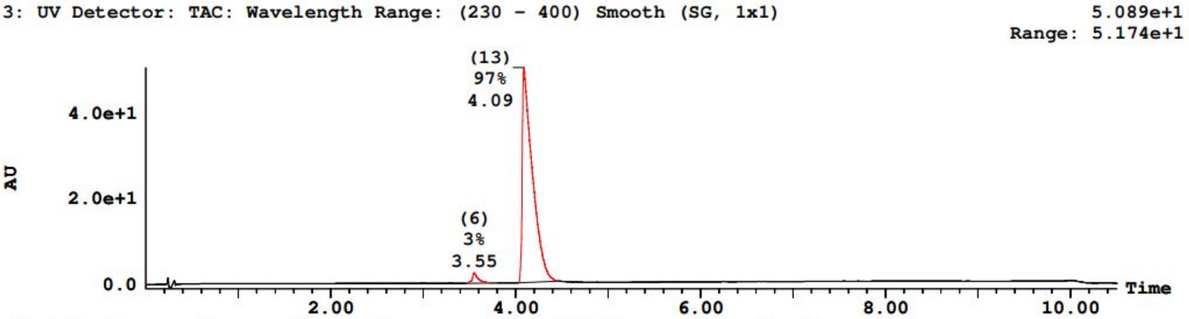

Peak Number	Compound	Time	Area %Total	Mass Found
6		3.55	2.83	Not Found
13		4.09	97.17	Not Found

Peak ID	Compound	Time	Mass Found
13		4.09	Not Found

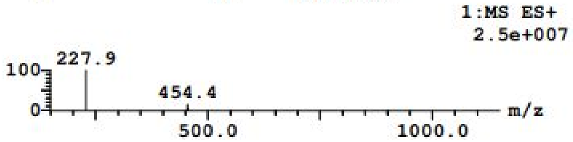

Compound 29

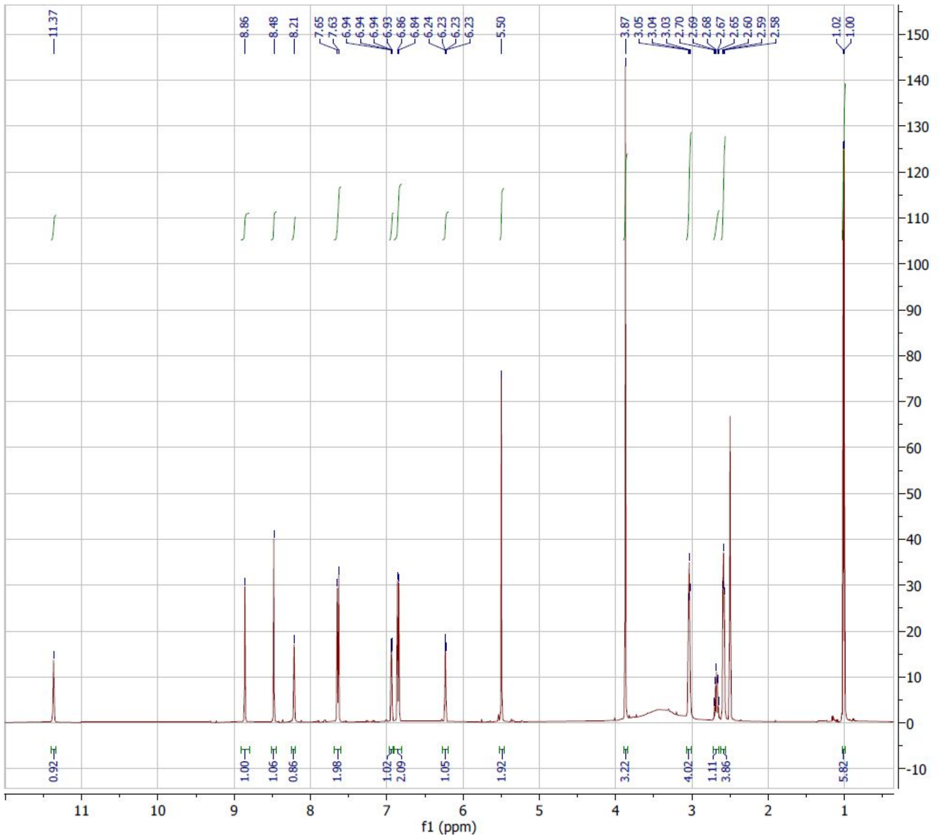

	Parameter	Value
1	Title	alborada_ATL-F-007
2	Origin	Bruker BioSpin GmbH
3	Solvent	DMSO
4	Temperature	298.0
5	Pulse Sequence	zg
6	Experiment	
7	Probe	5 mm PABBO BB-1H/ D Z-GRD Z104275/ 0242
8	Number of Scans	1
9	Receiver Gain	18.9
10	Relaxation Delay	0.1000
11	Pulse Width	10.5000
12	Presaturation Frequency	
13	Modification Date	2025-02-24T16:49:56
14	Spectrometer Frequency	500.05
15	Spectral Width	10000.0
16	Lowest Frequency	-2003.7
17	Nucleus	1H
18	Acquired Size	32768
19	Spectral Size	65536
20	Digital Resolution	

^{1}H NMR (500 MHz, DMSO) δ 11.37 (s, 1H), 8.86 (s, 1H), 8.48 (s, 1H), 8.21 (s, 1H), 7.64 (d, *J* = 9.0 Hz, 2H), 6.94 (dd, *J* = 3.4, 2.3 Hz, 1H), 6.85 (d, *J* = 9.1 Hz, 2H), 6.23 (dd, *J* = 3.4, 1.9 Hz, 1H), 5.50 (s, 2H), 3.87 (s, 3H), 3.07 – 3.01 (m, 4H), 2.68 (dt, *J* = 13.0, 6.5 Hz, 1H), 2.62 – 2.56 (m, 4H), 1.01 (d, *J* = 6.5 Hz, 6H).

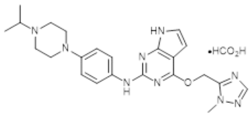

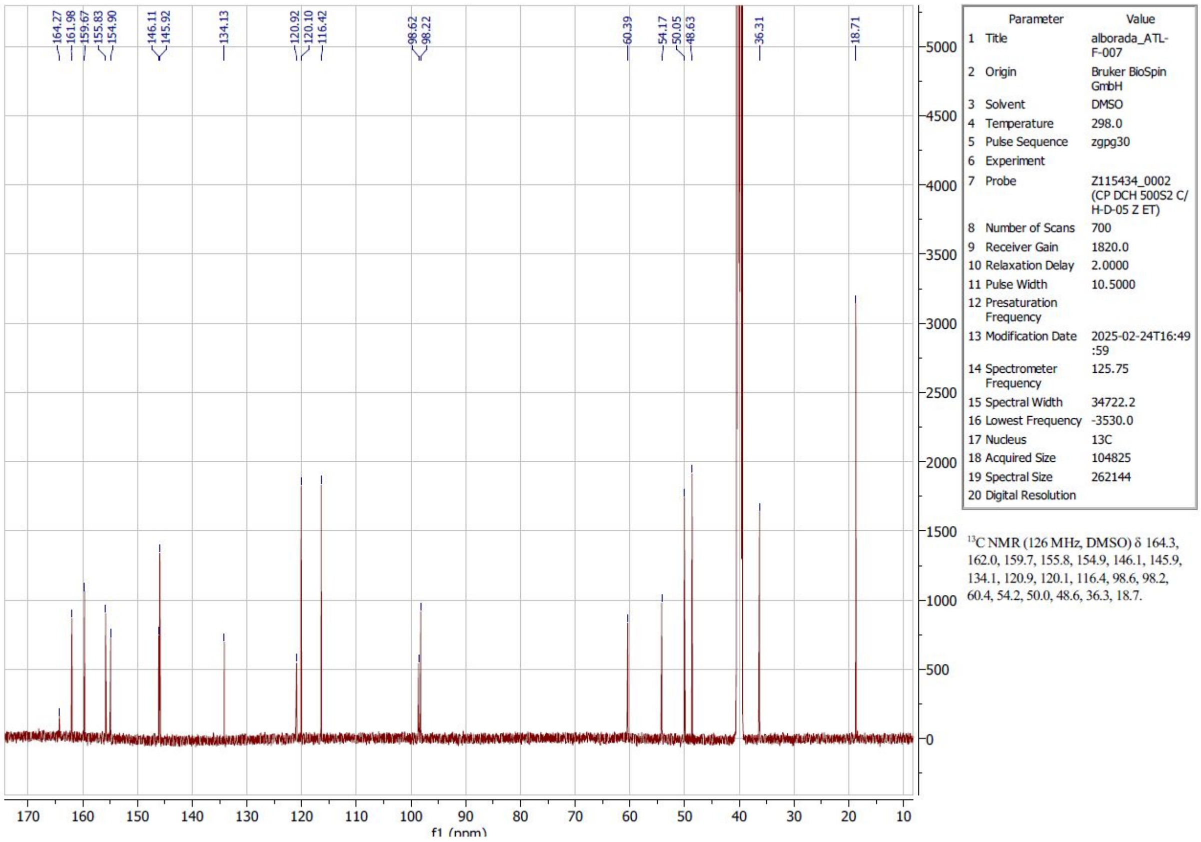

	Parameter	Value
1	Title	alborada_ATL-F-007
2	Origin	Bruker BioSpin GmbH
3	Solvent	DMSO
4	Temperature	298.0
5	Pulse Sequence	zgpg30
6	Experiment	
7	Probe	Z115434_0002 (CP DCH 500S2 C/H-D-05 Z ET)
8	Number of Scans	700
9	Receiver Gain	1820.0
10	Relaxation Delay	2.0000
11	Pulse Width	10.5000
12	Presaturation Frequency	
13	Modification Date	2025-02-24T16:49:59
14	Spectrometer Frequency	125.75
15	Spectral Width	34722.2
16	Lowest Frequency	-3530.0
17	Nucleus	13C
18	Acquired Size	104825
19	Spectral Size	262144
20	Digital Resolution	

^{13}C NMR (126 MHz, DMSO) δ 164.3, 162.0, 159.7, 155.8, 154.9, 146.1, 145.9, 134.1, 120.9, 120.1, 116.4, 98.6, 98.2, 60.4, 54.2, 50.0, 48.6, 36.3, 18.7.

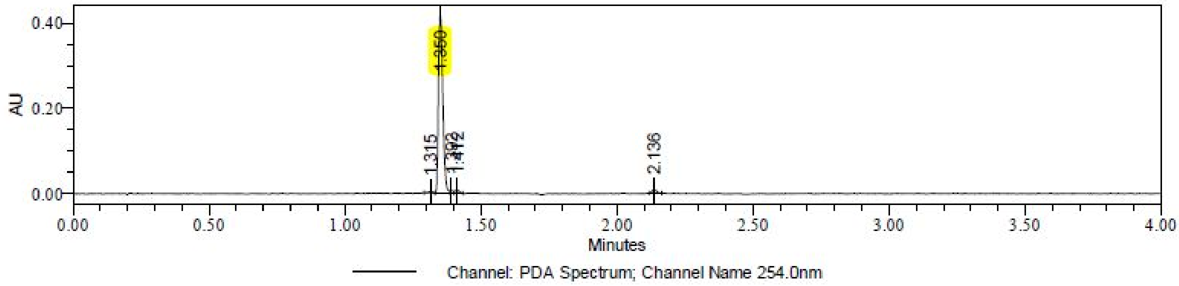

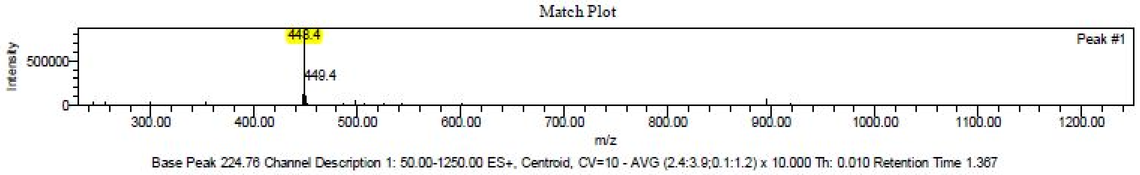

Compound 31

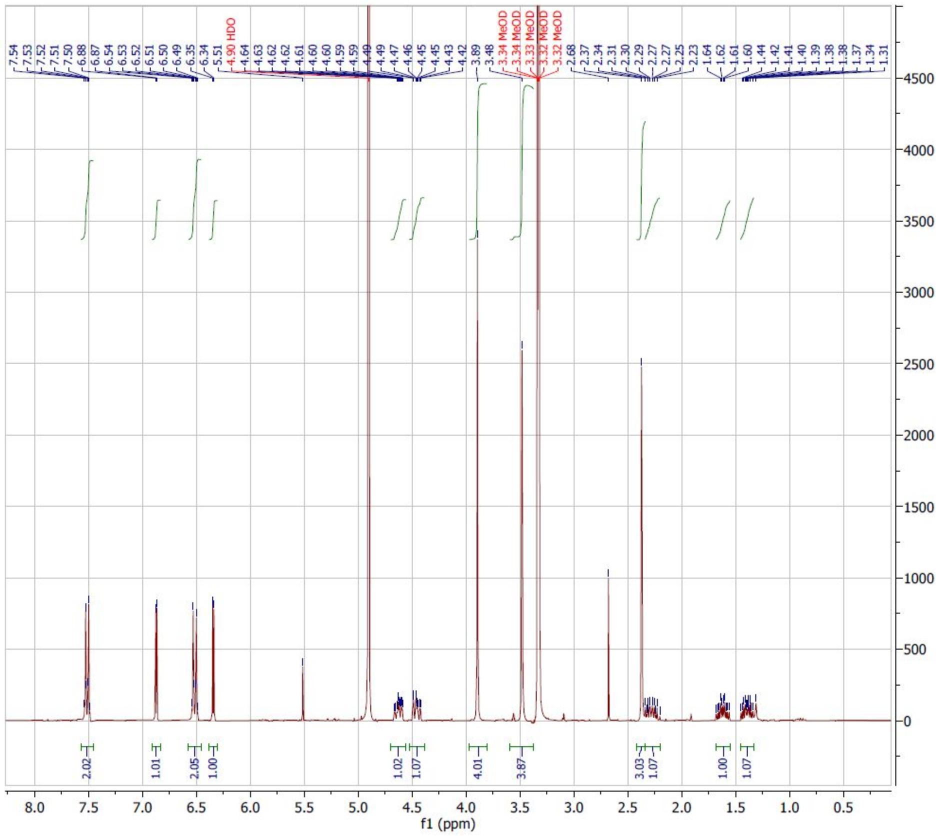

	Parameter	Value
1	Title	HKB8-079-3.10.fid
2	Origin	Bruker BioSpin GmbH
3	Solvent	MeOD
4	Temperature	295.6
5	Pulse Sequence	zg30
6	Experiment	1D
7	Probe	5 mm PABBO BB-1H/ D Z-GRD Z104275/ 0242
8	Number of Scans	128
9	Receiver Gain	191.9
10	Relaxation Delay	1.0000
11	Pulse Width	14.0000
12	Presaturation Frequency	
13	Modification Date	2021-09-16T11:40:52
14	Spectrometer Frequency	300.13
15	Spectral Width	6009.6
16	Lowest Frequency	-1151.4
17	Nucleus	1H
18	Acquired Size	32768
19	Spectral Size	65536
20	Digital Resolution	

^{1}H NMR (300 MHz, MeOD) δ 7.57 – 7.45 (m, 2H), 6.87 (d, J = 3.5 Hz, 1H), 6.57 – 6.46 (m, 2H), 6.34 (d, J = 3.5 Hz, 1H), 4.70 – 4.56 (m, 1H), 4.53 – 4.39 (m, 1H), 3.97 – 3.80 (m, 4H), 3.59 – 3.37 (m, 4H), 2.37 (s, 3H), 2.34 – 2.20 (m, 1H), 1.68 – 1.55 (m, 1H), 1.45 – 1.33 (m, 1H).

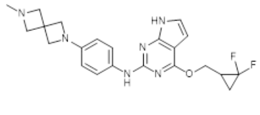

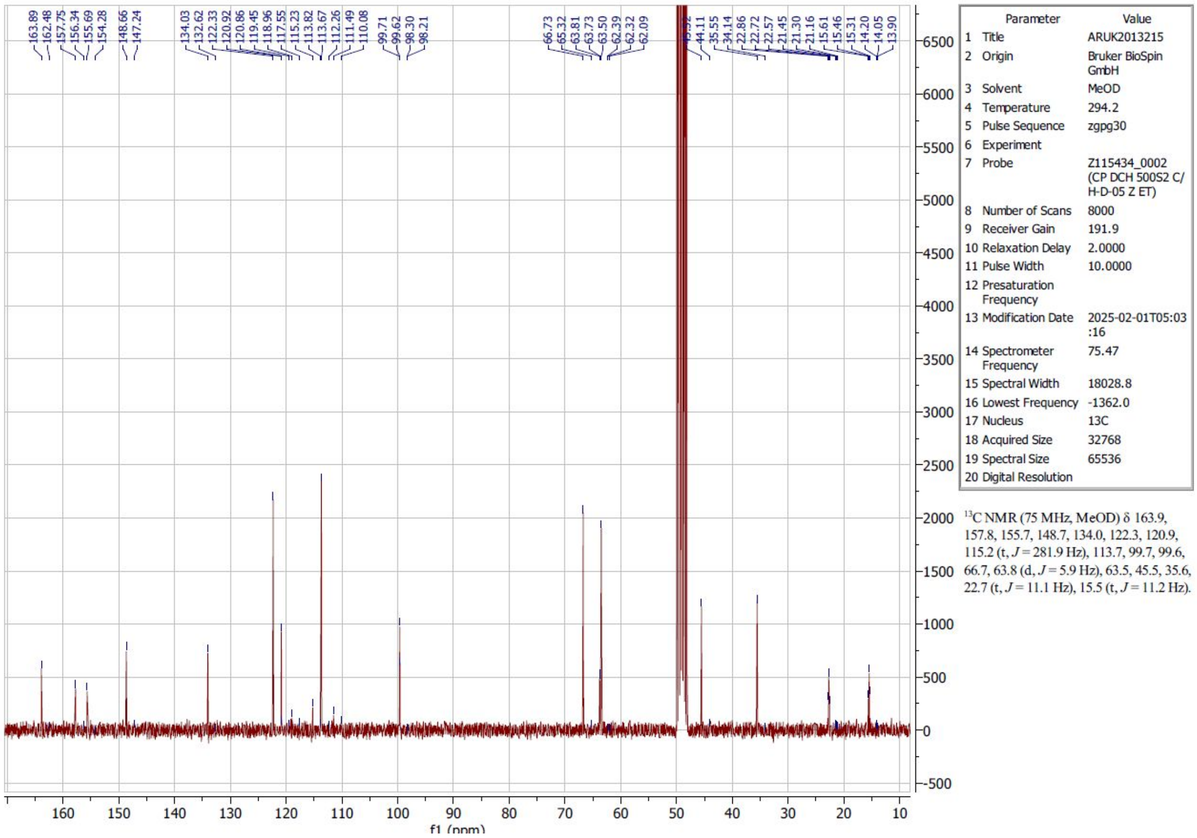

Parameter		Value
1	Title	ARUK2013215
2	Origin	Bruker BioSpin GmbH
3	Solvent	MeOD
4	Temperature	294.2
5	Pulse Sequence	zgpg30
6	Experiment	
7	Probe	Z115434_0002 (CP DCH 500S2 C/ H-D-05 Z ET)
8	Number of Scans	8000
9	Receiver Gain	191.9
10	Relaxation Delay	2.0000
11	Pulse Width	10.0000
12	Presaturation Frequency	
13	Modification Date	2025-02-01T05:03:16
14	Spectrometer Frequency	75.47
15	Spectral Width	18028.8
16	Lowest Frequency	-1362.0
17	Nucleus	13C
18	Acquired Size	32768
19	Spectral Size	65536
20	Digital Resolution	

^{13}C NMR (75 MHz, MeOD) δ 163.9, 157.8, 155.7, 148.7, 134.0, 122.3, 120.9, 115.2 (t, J = 281.9 Hz), 113.7, 99.7, 99.6, 66.7, 63.8 (d, J = 5.9 Hz), 63.5, 45.5, 35.6, 22.7 (t, J = 11.1 Hz), 15.5 (t, J = 11.2 Hz).

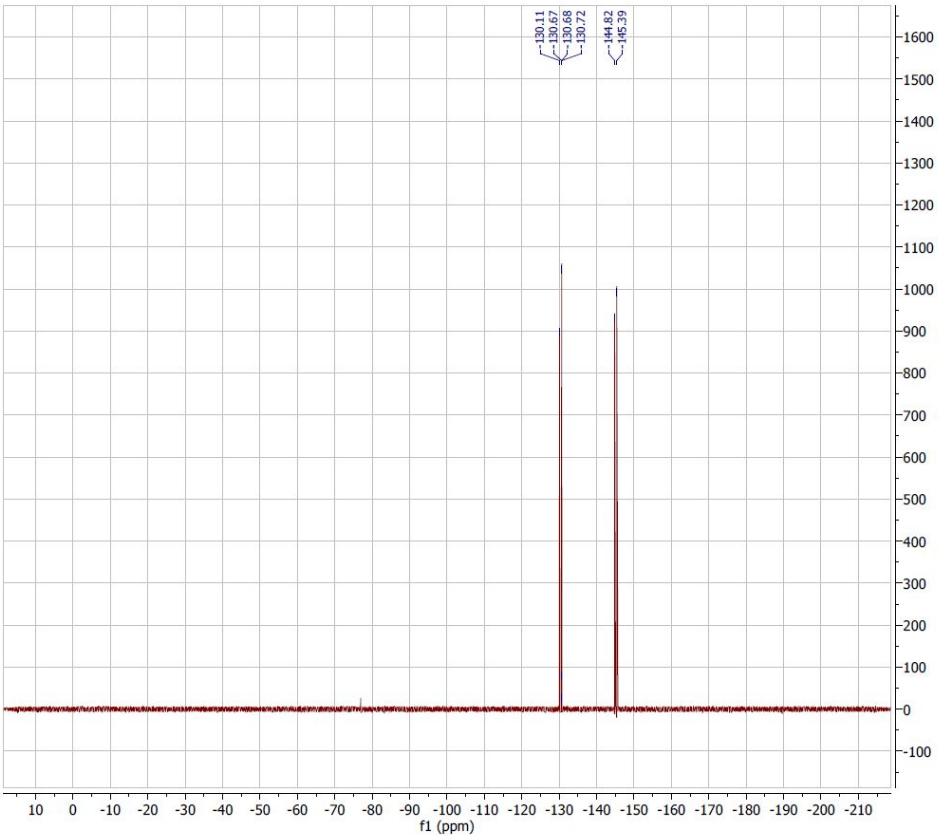

Parameter		Value
1	Title	HKB8-079-3.12.fid
2	Origin	Bruker BioSpin GmbH
3	Solvent	MeOD
4	Temperature	296.0
5	Pulse Sequence	zgfhigqn.2
6	Experiment	1D
7	Probe	5 mm PABBO BB-1H/ D Z-GRD Z104275/ 0242
8	Number of Scans	128
9	Receiver Gain	191.9
10	Relaxation Delay	1.0000
11	Pulse Width	15.0000
12	Presaturation Frequency	
13	Modification Date	2021-09-16T11:47:32
14	Spectrometer Frequency	282.38
15	Spectral Width	66964.3
16	Lowest Frequency	-61722.4
17	Nucleus	19F
18	Acquired Size	65536
19	Spectral Size	131072
20	Digital Resolution	

^{19}F NMR (282 MHz, MeOD) δ -130.39 (d, J = 160.3 Hz), -145.10 (d, J = 160.1 Hz).

Column Name ACQUITY UPLC® HSS C18 1.8µm

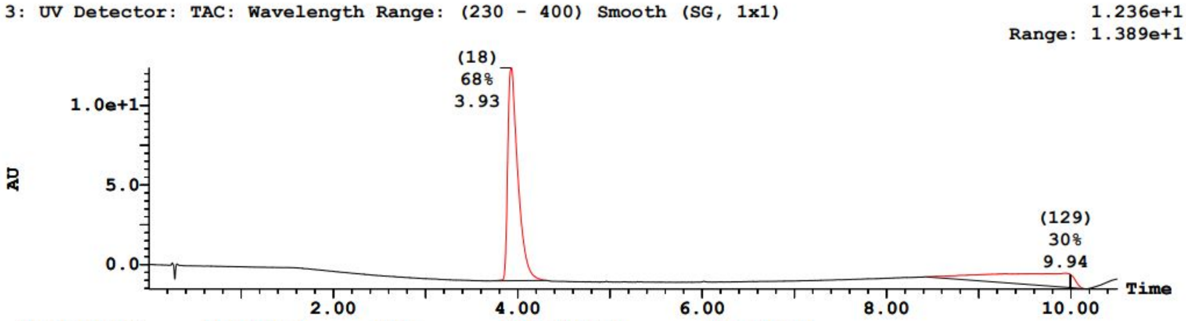

Peak Number	Compound	Time	Area %Total	Mass Found
18		3.93	67.80	Not Found

Peak ID	Compound	Time	Mass Found
18		3.92	Not Found

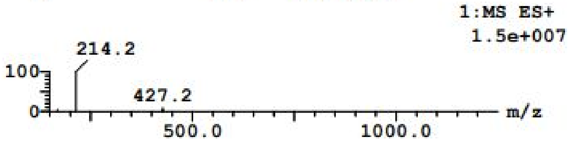

References

(1) Hardcastle, I. R.; Arris, C. E.; Bentley, J.; Boyle, F. T.; Chen, Y.; Curtin, N. J.; Endicott, J. A.; Gibson, A. E.; Golding, B. T.; Griffin, R. J.; Jewsbury, P.; Menyerol, J.; Mesguiche, V.; Newell, D. R.; Noble, M. E. M.; Pratt, D. J.; Wang, L. Z.; Whitfield, H. J. N2-Substituted O6-Cyclohexylmethylguanine Derivatives: Potent Inhibitors of Cyclin-Dependent Kinases 1 and 2. *J. Med. Chem.* **2004**, *47* (15), 3710–3722. https://doi.org/10.1021/jm0311442.

(2) Rooney, T. P. C.; Aldred, G. G.; Winpenny, D.; Scott, H.; Willems, H. M. G.; Voytyuk, I.; Clarke, J. H.; Boffey, H. K.; Andrews, S. P.; Skidmore, J. Development of the Pyrido[2,3-d]Pyrimidin-7(8H)-One Scaffold toward Potent and Selective NUAK1 Inhibitors. *ACS Med. Chem. Lett.* **2025**, *16* (2), 327-335. https://doi.org/10.1021/acsmedchemlett.4c00579.

(3) El kihel, A.; Essassi, E. M.; Bauchat, P. 1H and 13C NMR spectra of condensed benzimidazole and imidazobenzodiazepines. *Arabian Journal of Chemistry* **2012,** *5* (4) 523–526. https://doi.org/10.1016/j.arabjc.2010.09.021.

(4) Free base used for in vivo testing.
